# Supplementary material for: FOXA1 regulates alternative splicing in prostate cancer
Source: Cell Rep. 2022 Sep 27;40(13):111404. doi: 10.1016/j.celrep.2022.111404 (PMC9532847; doi:10.1016/j.celrep.2022.111404)
Supplement: Document S1. Figures S1–S6 [file mmc1.pdf]

Cell Reports, Volume *40*

Supplemental information

FOXA1 regulates alternative splicing in prostate cancer

Marco Del Giudice, John G. Foster, Serena Peirone, Alberto Rissone, Livia Caizzi, Federica Gaudino, Caterina Parlato, Francesca Anselmi, Rebecca Arkell, Simonetta Guarrera, Salvatore Oliviero, Giuseppe Basso, Prabhakar Rajan, and Matteo Cereda

Figure S1. Transcriptional regulation of SRGs in PC, related to figure 1.

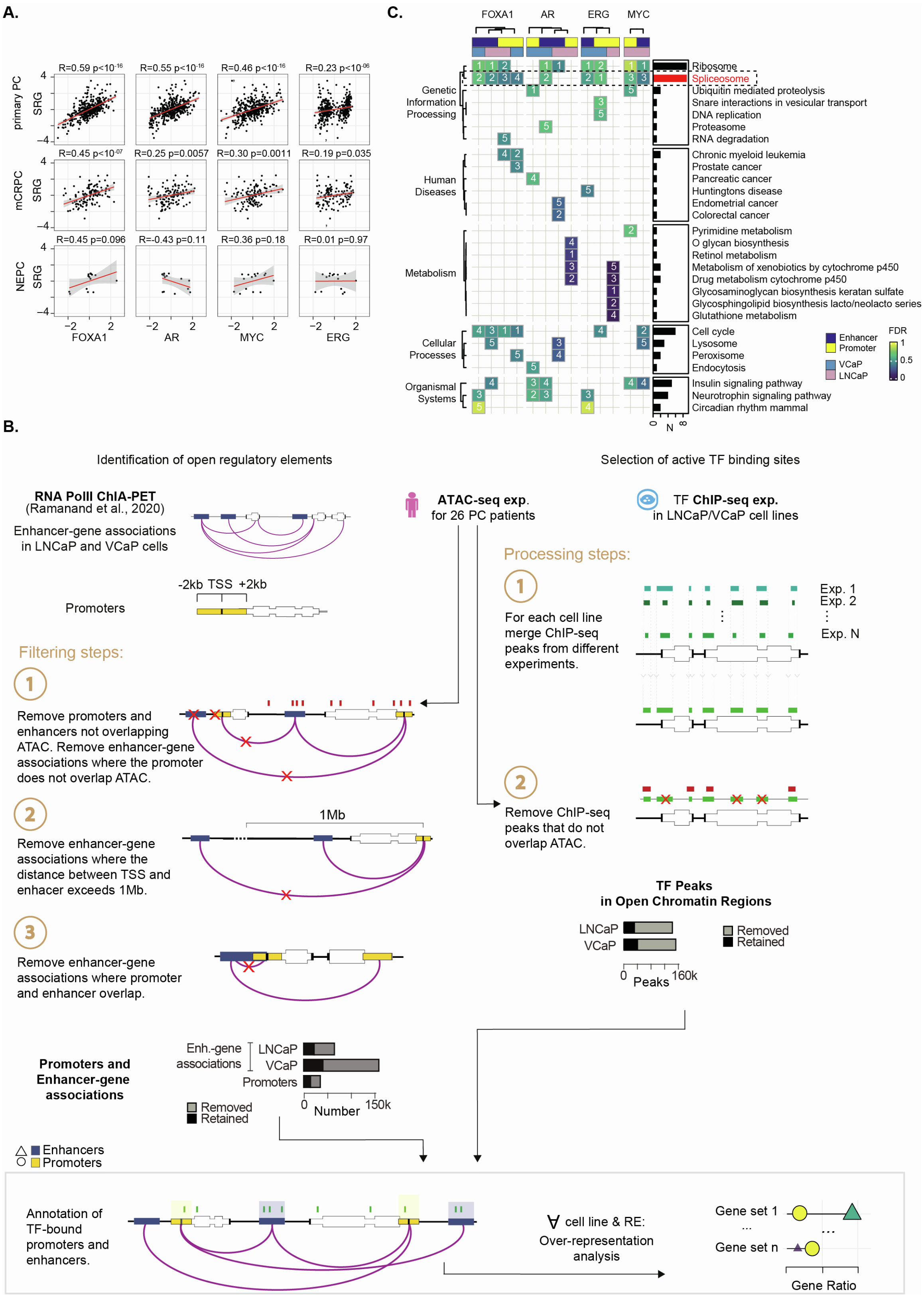

Figure S1. (A) Scatter plots between the scaled expression of each TF and the cumulative expression of SRGs in primary PCs (top), mCRPCs, (middle), and NEPCs (bottom). Pearson's correlation and corresponding p-value are shown for each plot. (B) Schematic representation of the pipeline used to identify active TF binding sites and their over-

representation in gene sets. (C) Over representation analysis performed on genes with active TF binding sites in KEGG pathways. Top five most significant gene sets are shown for each condition. Cell color indicates the statistical significance (FDR) and numbers indicate the rank of the gene set. Bar plots on the right show the number (N) of conditions in which the gene set is significantly enriched.

Figure S2. SRG differential expression analyses, related to figure 1.

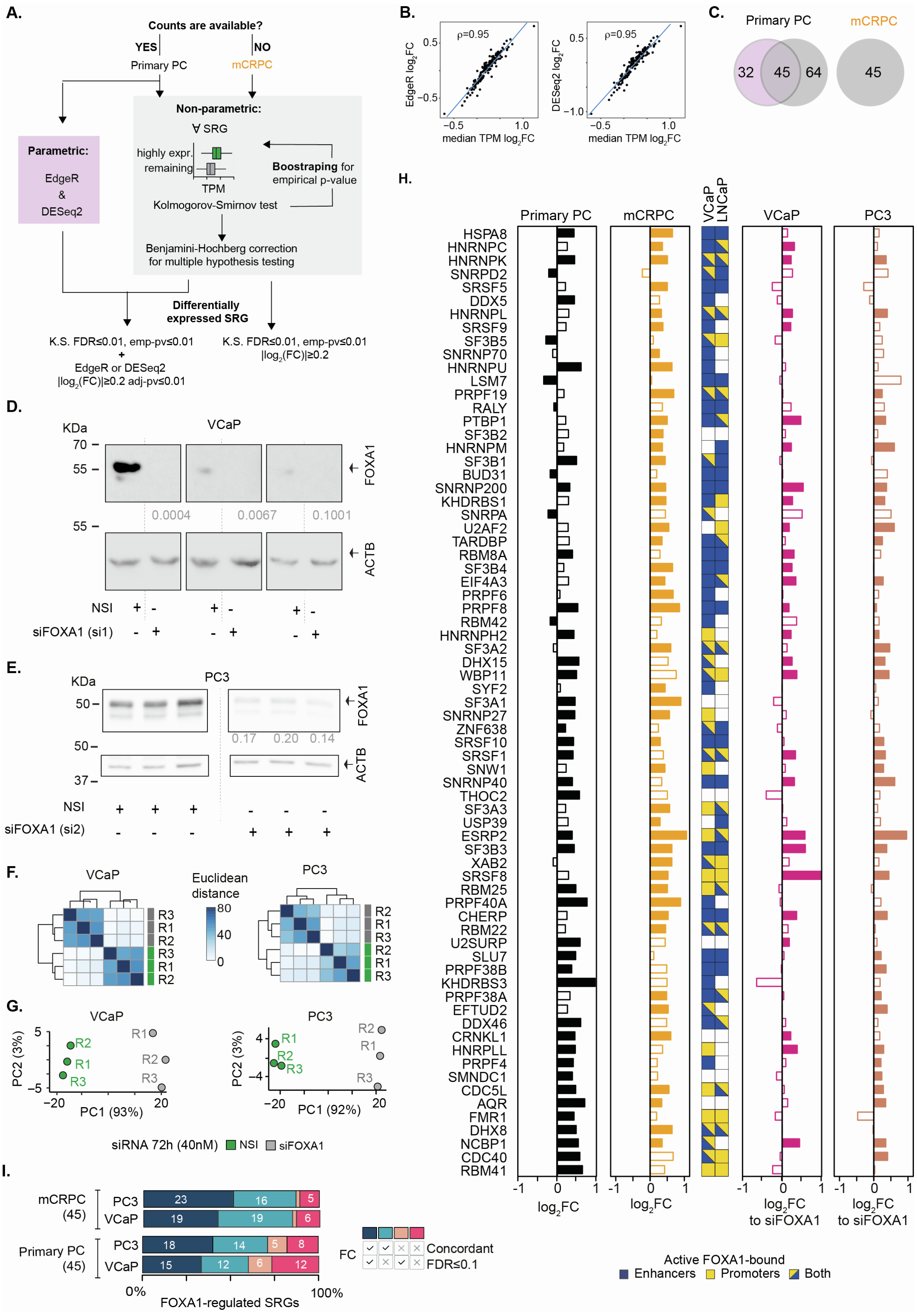

Figure S2. (A) Schematic representation of the pipeline used to detect SRGs that are differentially expressed (DE) in primary PCs and mCRPCs upon high *FOXA1* expression. (B) Scatter plots showing the correlation between median $\log_2$ Fold Change (FC) and FC measured by EdgeR (right) or DESeq2 (left) for the SRGs set. The value of Pearson's correlation coefficient (r) is reported. (C) Venn diagrams showing the number of SRGs identified as DE by the parametric (pink) and non-parametric (grey) approaches. (D,E) Representative Western blotting images of whole cell lysates from (D) VCaP and (E) PC3 cells used for RNA-seq analysis upon depletion of FOXA1 with one siRNA duplex (si1 or si2, 40nM, or 20nM, respectively for 72 hours) using antibodies to FOXA1 and ACTB. ACTB-normalised protein expression compared to control, calculated by densitometric band quantitation, are shown below the upper blot image. (F) Hierarchically clustered heatmaps of Euclidean distance between expression values for VCaP and PC3. (G) Scatter plot of the first two components of principal component analysis for VCaP and PC3 datasets. Percentage of variance explained by each component is reported on each axis. (H) FC in TPMs of 71 FOXA1-regulated SRGs between FOXA1 highly expressing and remaining primary PCs (black) and mCRPCs (orange). Filled bars indicate significant expression changes. Central heatmap annotation indicates the presence of active FOXA1 binding sites on SRG promoters and/or cognate enhancers. Bar plots on the right indicate FC in expression levels upon FOXA1 depletion (*i.e.* NSI versus siFOXA1) of each SRG in VCaP (magenta) and PC3 (brown) cells. Filled bars indicate significant expression changes. (I) Bar plots showing the fractions of FOXA1-regulated SRGs in primary PCs and mCRPCs that changed in expression upon FOXA1 depletion in VCaP and PC3 cells. Absolute number of FOXA1-regulated SRGs are reported. In total, 23 and 35 SRGs were concordantly regulated by FOXA1 in both cell lines to a similar magnitude to primary PCs and mCRPCs. Consistent with the metastatic origin of the cell lines, a higher number of differentially expressed SRGs in mCRPCs were concordantly up-regulated in the cell lines than the SRGs that were DE in primary PC. On average, 76% of the FOXA1-regulated SRGs in primary PCs and/or mCRPCs were concordantly regulated by FOXA1 in the two cell lines

Figure S3. Transcriptional architecture of HNRNPK, HNRNPL, SRSF1, related to figure 1.

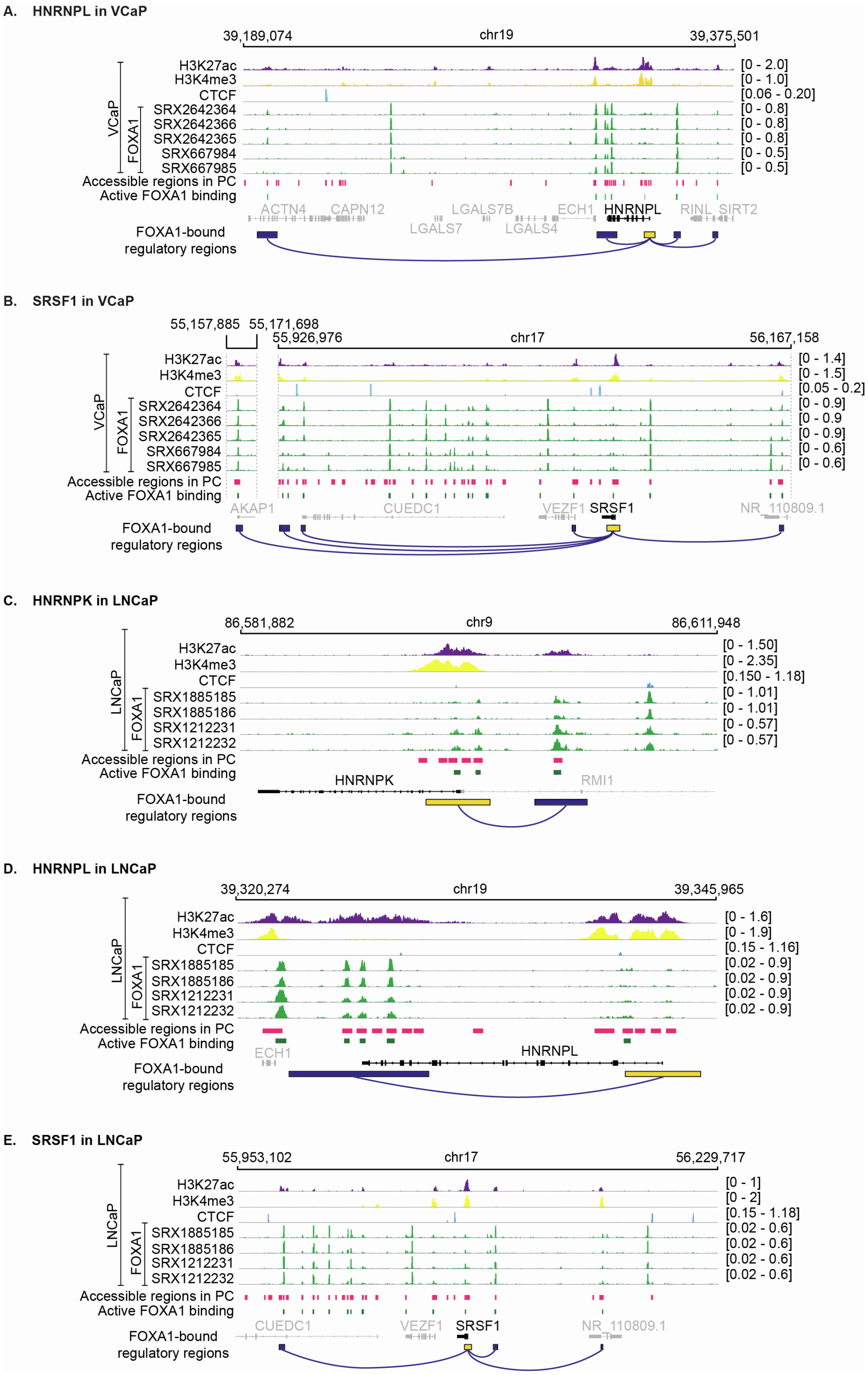

Figure S3. For each panel, ChIP-seq density read tracks of H3K27ac, H3K4me3, CTCF (2 overlayed experiments for VCaP) and FOXA1 (5 and 4 experiments for VCaP and LNCaP, respectively) are shown with recurrent accessible regions of primary PC from ATAC-seq experiments, active FOXA1 binding sites, and RNA PolII ChIA-PET-derived FOXA1-bound promoters (yellow) and cognate enhancers (blue).

Figure S4. ***In vitro* validations in PC cell lines, related to figures 1, 5 and 6.**

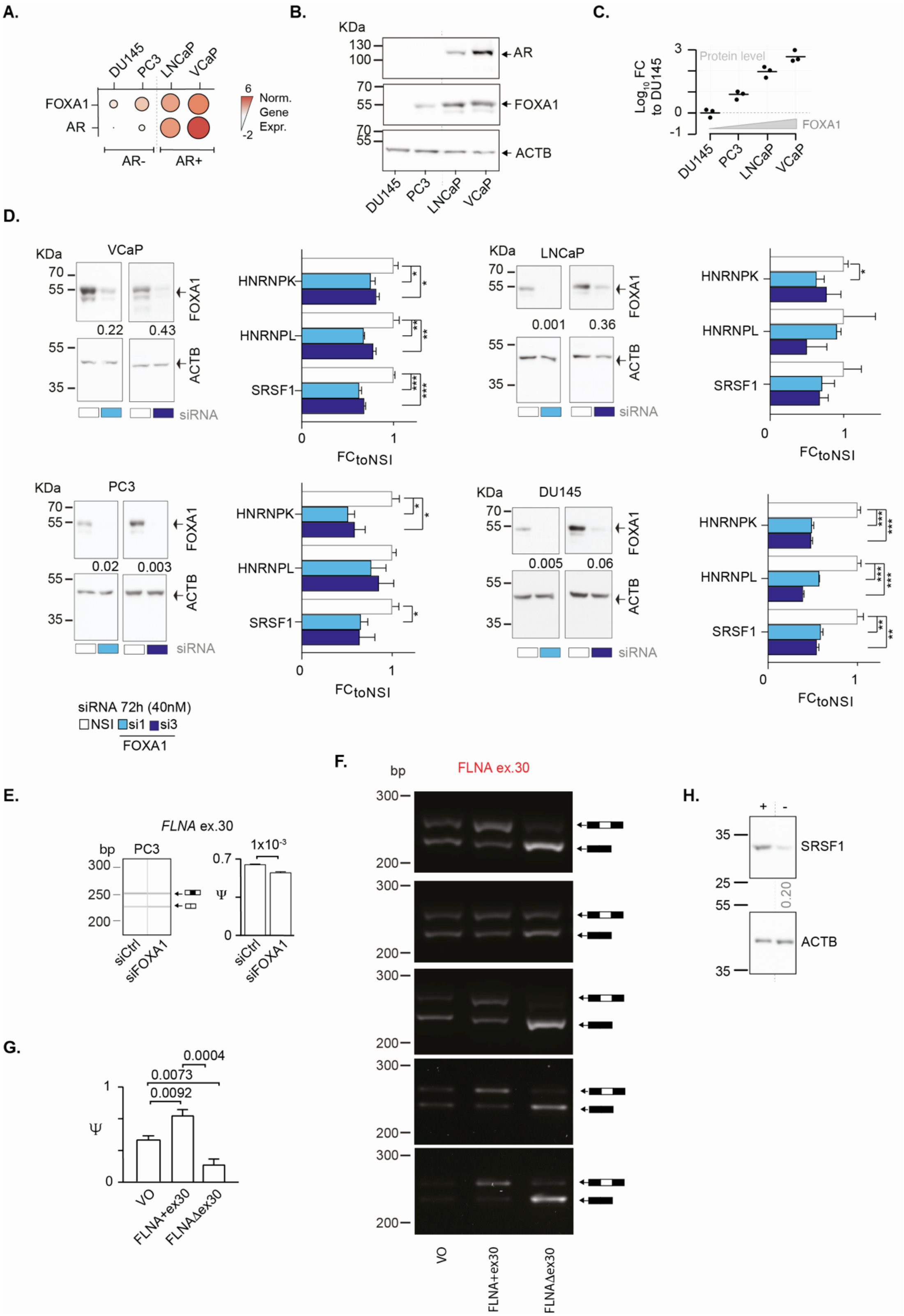

Figure S4. (A) *FOXA1* and *AR* normalized gene expression levels in four PC cell lines as measured in Cancer Cell Line Encyclopedia RNA-Seq data. (B) Representative Western blotting images of whole cell lysates from PC cell lines using

antibodies to AR, FOXA1 and ACTB. (C) Densitometric band quantitation results showing the mean log_{10} relative normalized FC in FOXA1 protein expression relative to DU145 cells (three independent experiments). Consistent with Cancer Cell Line Encyclopedia RNA-Seq data, we identified the highest and lowest levels of FOXA1 protein expression in the VCaP cells and DU145 cells, respectively. (D) Representative Western blotting images of whole cell lysates from PC cell lines (left panel) upon FOXA1 depletion with the two siRNA duplexes (si1 or si3, 40nM for 72 hours) using antibodies to FOXA1 and ACTB. We employed one siRNA duplex used for RNA-seq plus a further independent duplex to reduce artifactual hits. ACTB-normalised protein expression compared to control, calculated by densitometric band quantitation, is shown below the upper blot images. Bar plots (right panel) depict the mean (± standard error) expression changes of candidate SRGs measured by qRT-PCR upon FOXA1 depletion from biological triplicate samples. Stars *, **,*** depict p-values ≤0.05, 0.01 and 0.001, respectively. (E) *FLNA* exon 30 inclusion changes in PC3 cells were measured by endpoint PCR splicing assays upon FOXA1 depletion with one siRNA duplex (si1, 40nM for 72 hours). Two-tailed T-test was used to compare conditions. (F-G) Total (endogenous and exogenous) *FLNA* exon 30 expression in PC3 cells was measured by endpoint PCR assays following transfection with 2 μg of plasmid DNA vector encoding FLNA with or without exon 30 (*i.e.* FLNA+ex30 or FLNAΔex30, respectively, or vector only (VO) control). (F) Agarose gel electrophoresis images show two bands representing *FLNA* transcripts including or excluding exon 30 which were quantified to determine Ψ. (G) Two-tailed T-test was used to compare the five biological replicates. (H) Representative Western blotting images of whole cell lysates from PC3 cells upon SRSF1 depletion with one siRNA duplex (40nM for 72 hours) using antibodies to SRSF1 and ACTB. ACTB-normalised protein expression compared to control (NSI), calculated by densitometric band quantitation, is shown below the SRSF1 blot image.

Figure S5. Analyses of FOXA1-mediated AS regulation, related to figures 2-5.

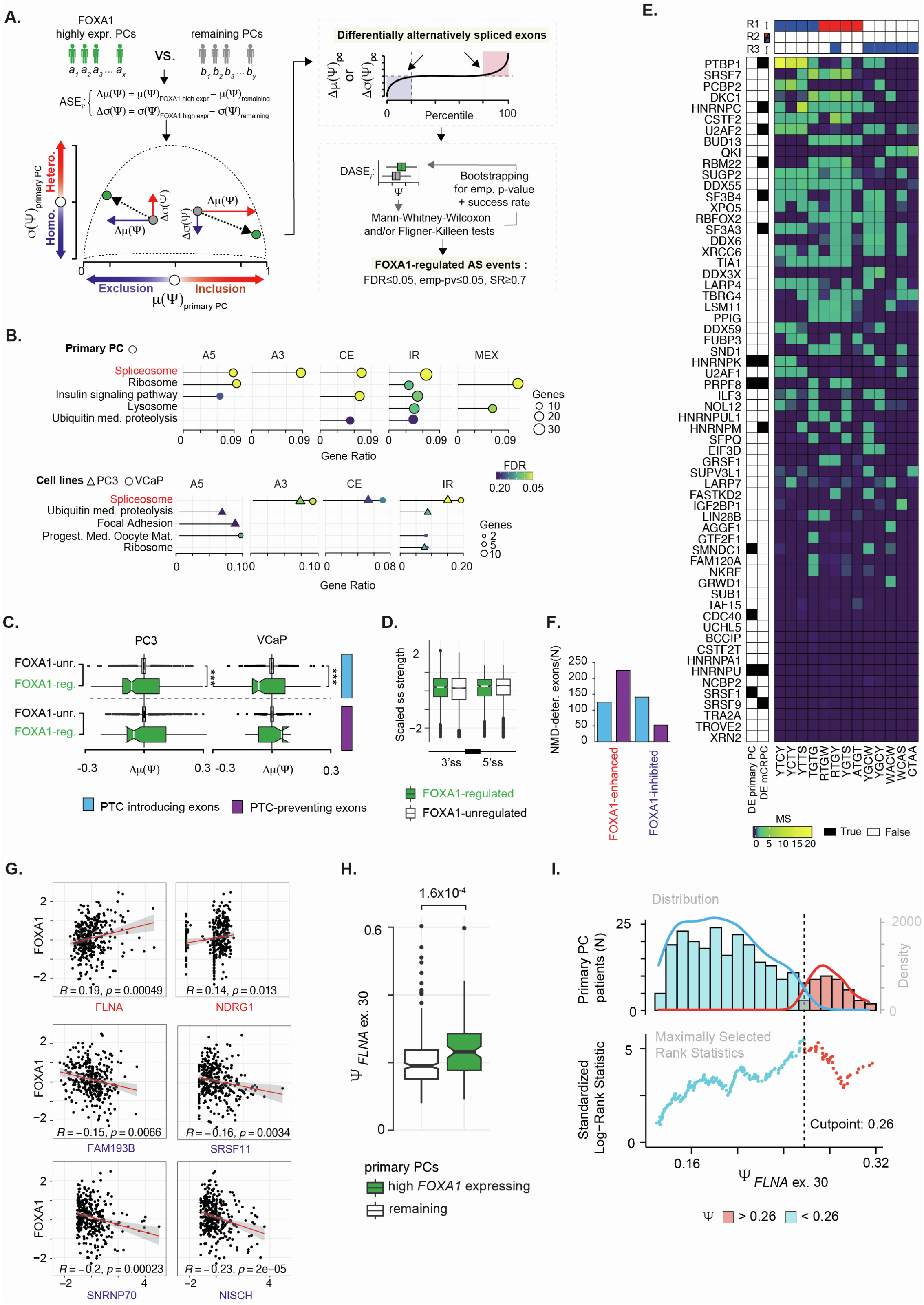

Figure S5. (A) Schematic representation of the pipeline used to identify FOXA1-regulated events in primary PC. Alternatively spliced exons (ASEs) are defined by mean (μ) and standard deviation (σ) inclusion (Ψ) changes (Δ) between

high FOXA1 expression tumors and remaining ones. Trajectory (represented as the arrow) of each ASEs is defined by $\Delta\mu(\Psi)$ and $\Delta\sigma(\Psi)$ changes (Δ) toward high FOXA1 expression. (B) Over representation analysis performed on genes harbouring FOXA1-regulated AS events, stratified by event type, in primary PCs and PC cell lines. Shape size and gene ratio indicate the number and the fraction of selected genes in each gene set, respectively. Color key represents the significance of the enrichment. The top five gene sets with FDR<0.25 are shown. CE, Cassette Exon; A3, Alternative 3' splice site; A5, Alternative 5' splice site; IR, Intron Retention; MEX, Mutually Exclusive exons. (C) Distribution of mean inclusion changes of NMD-determinant FOXA1-regulated and -unregulated exons in VCaP (upper panel) and PC3 cells (lower panel). Stars indicate statistical significance of a two-tailed Wilcoxon Rank Sum test (***, p-value<10^{-3}). (D) Box plots showing the scaled MaxEntScan strength of the 3' and 5' splice sites in FOXA1-regulated (green) and -unregulated (white) CEs. (E) Heatmap showing the association between enriched multivalent RNA motifs and cognate SRGs in terms of maximum Matching Score (MS). Top annotation heatmap shows indicates the regions at exon/intron junctions where motifs were enriched at inhibited (blue) or enhanced (red) exons. Left annotation indicates whether SRGs are DE in primary PCs or mCRPCs. (F) Number of NMD-determinant exons that are inhibited (blue) or enhanced (red) by FOXA1. (G) Scatter plots between scaled inclusion level of each of the six harmful NMD-determinant exons and scaled expression of *FOXA1* in primary PC. Pearson's correlation and corresponding p-value are shown for each plot. (H) Distributions of *FLNA* exon 30 inclusion levels (Ψ) across primary PC samples stratified on high *FOXA1* expression. The significance of a two-tailed Wilcoxon Rank Sum test comparing the two groups is shown. (I) Distribution of the standardized Log-Rank statistic as a function of the cutpoint on *FLNA* exon 30 Ψ (bottom panel). Top panel shows the distribution of the number of primary PC patients as a function of *FLNA* exon 30 Ψ. Patients are stratified according to the cutpoint. Density distributions of the two groups are superimposed.

Figure S6. FOXA1 regulates AS regardless of tumor purity constraints, related to figures 1 and 2.

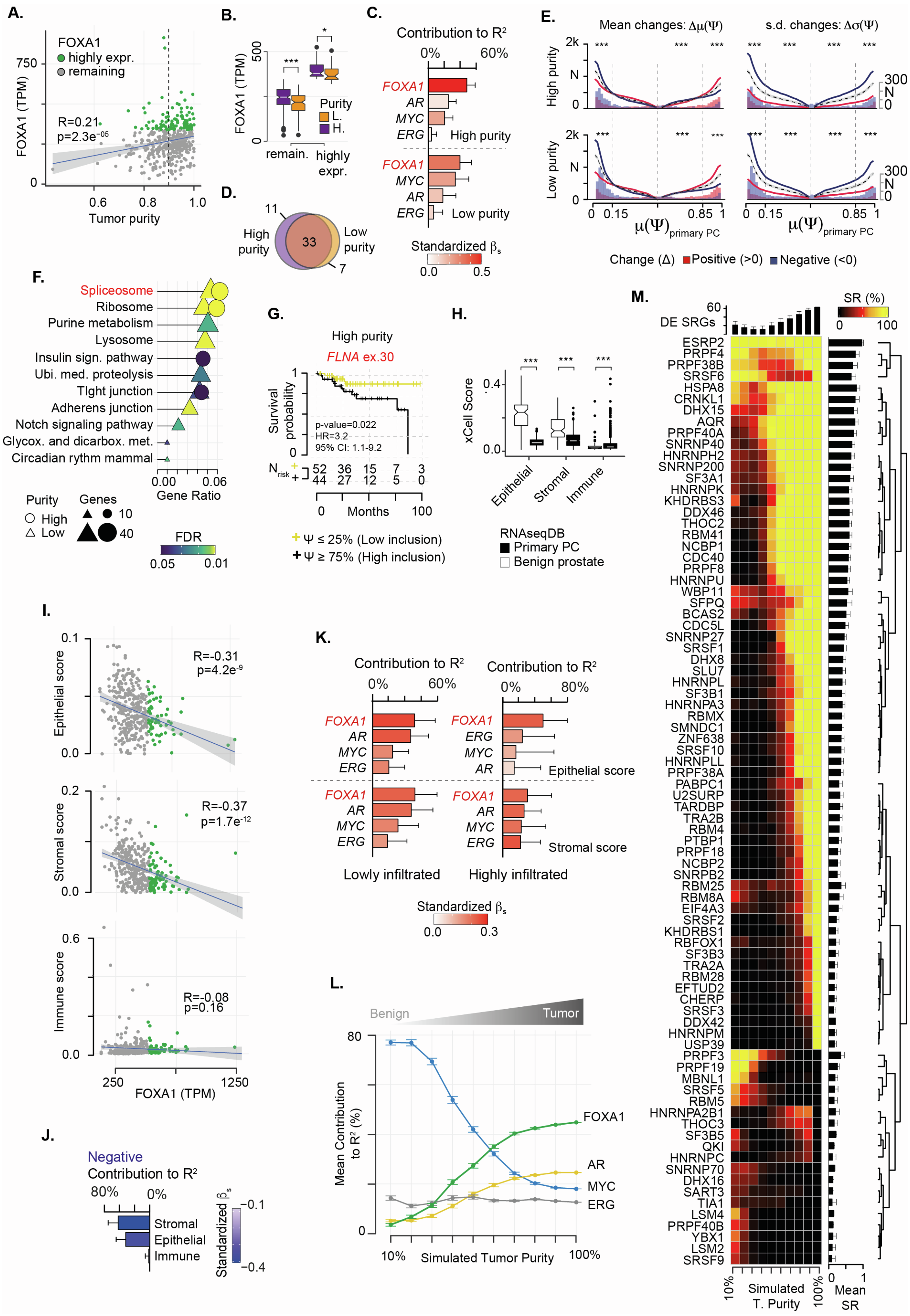

Figure S6. (A) Scatter plot of *FOXA1* expression (TPM) and tumor purity for primary PCs. Dot color indicates sample stratification according to high *FOXA1* expression (i.e. ≥75th percentile of expression distribution). Pearson's correlation coefficient and p-value are shown. 90% of samples show less than 20% of contamination (median purity = 91%) (B) Boxplots represent *FOXA1* expression levels in primary PCs with high FOXA1 expression and remaining ones stratified into "high purity" (i.e. purity ≥ 90%) and "low purity" (i.e. purity < 90%) samples. High purity groups exhibit a greater overall expression of FOXA1 than the low purity groups. This is consistent with a greater infiltration of low purity samples with normal non-cancerous cells, which express FOXA1 at lower levels than cancer cells (Parolia et al. 2019). (C) Results of multivariable covariance analysis between the cumulative expression of SRGs and the expression of TFs in primary PCs for the high purity and low purity samples. Color key indicates the standardized β coefficients of the model. FOXA1 is the strongest contributor to SRG expression regardless of purity constraints. In the high purity tumors, FOXA1 contributes to SRG expression significantly more than the second-ranked TF (two-sided Z-test p-value=0.01). (D) Venn diagram depicts 40 and 44 differentially expressed (DE) SRGs by FOXA1 in the "high" and "low purity" samples, respectively. 73% of DE SRGs were still differentially expressed by FOXA1 in both "high" and "low purity" samples (E) The cumulative distribution of the number (N) of exons with either positive (red) or negative (blue) changes are reported ranging from $\mu(\Psi)_{\text{primary PC}}$ of 0.5 (*i.e.* mixed isoforms) to the boundaries of 0 and 1 (*i.e.* dominant isoforms) model for the high purity and low purity samples. Dashed lines represent the expected mean cumulative distribution of events with inclusion changes generated by 1,000 Monte Carlo simulations. Grey area represents confidence intervals (5%-95%). Histograms of the number (N) of exons with positive (red) and negative (blue) changes are superimposed on the x-axis, respectively. Stars indicate the significance of two tailed Exact Binomial test comparing the abundances of exons with positive and negative changes against a null hypothesis of equal probability (*i.e.* 0.5) in four groups of inclusion levels (***, p-value<10^{-3}). FOXA1-mediated AS calibration towards dominant isoforms is confirmed in both cohorts. (F) Enrichment of genes affected by FOXA1-regulated AS events in the high purity (circles) and low purity (triangles) group of samples. Shape size and gene ratio indicate the number and the fraction of selected genes in each pathway, respectively. Shape size ranges from 10 to 40. Color key represents the statistical significance of the enrichment. Only significantly enriched pathways (FDR<0.1) are shown. The enrichment of AS events in spliceosomal genes is confirmed in both cohorts. (G) Kaplan-Meier plot of disease-free survival for primary PC patients in the "high purity" group with low and high inclusion of *FLNA* exon 30. The number of patients at risk (N_{risk}) is tabulated at each time point on the x-axis. Univariate hazard ratio with 95% confidence intervals (CI) and two-tailed log-rank test p-value are shown. (H) Benign epithelial, stromal and immune xCell score distribution in primary PC and benign prostate tissue samples (***, Wilcoxon Rank Sum test p-value<10^{-3}). As expected, stromal and epithelial scores are enriched in benign tissues compared to tumors, whereas immune score is greater in primary PCs relative to benign tissues (I) Scatter plots between *FOXA1* expression and epithelial (top panel), stromal (middle panel) and immune (bottom panel) xCell scores in primary PC. Pearson's correlation coefficients and p-values are shown. Stromal and epithelial scores negatively correlate with *FOXA1* expression, indicating a lower infiltration in samples with high *FOXA1* expression. FOXA1 expression is not affected by the infiltration rate of immune cells. These infiltrate-specific scores are low in tumors (*i.e.* median score = 0.044 (stromal), 0.036 (epithelial), and 0.014 (immune)), in line with the average high purity of these primary PCs. (J) Results of multivariable covariance analysis between *FOXA1* expression and the epithelial, stromal and immune xCell score. Color key indicates the standardized β coefficients of the model. (K) Results of multivariable covariance analysis between the cumulative expression of SRGs and the expression of TFs in primary PCs stratified into "highly" and "lowly infiltrated" according to high values of epithelial and stromal xCell score (*i.e.* ≥75th percentile of each distribution). Color key indicates the standardized β coefficients of the model. FOXA1 is the strongest contributor to SRG expression regardless of the level of stromal and benign epithelial cell infiltration. (L) Mean relative contribution of each TF the cumulative expression of SRGs measured by multivariable covariance analysis in primary PCs at different levels of simulated tumor purity. Error bars indicate the standard error of the mean contribution across 100 Monte Carlo simulations. Increasing tumor purity (*i.e.* reducing normal cell infiltrates) potentiates the contribution of FOXA1 to SRG expression. (M) Heatmap showing, for each SRG, the success rate (SR, *i.e.* percentage of success across 100 Monte Carlo simulations) of being differentially expressed upon high *FOXA1* expression in the different simulated purity levels. Bar plots at the top indicate the average number (N) of differentially expressed SRGs for each purity level across simulations. Bar plots on the right indicate the average SR for each SRG across simulations. For both bar plots, error bars indicate the standard deviation. The number of SRGs that were DE by FOXA1 increases with tumor purity.
