# Supplementary material for: FOXA1 regulates alternative splicing in prostate cancer
Source: Cell Rep. 2022 Sep 27;40(13):111404. doi: 10.1016/j.celrep.2022.111404 (PMC9532847; doi:10.1016/j.celrep.2022.111404)
Supplement: Document S2. Article plus supplemental information [file mmc8.pdf]

Cell Reports

Article

FOXA1 regulates alternative splicing in prostate cancer

Graphical abstract

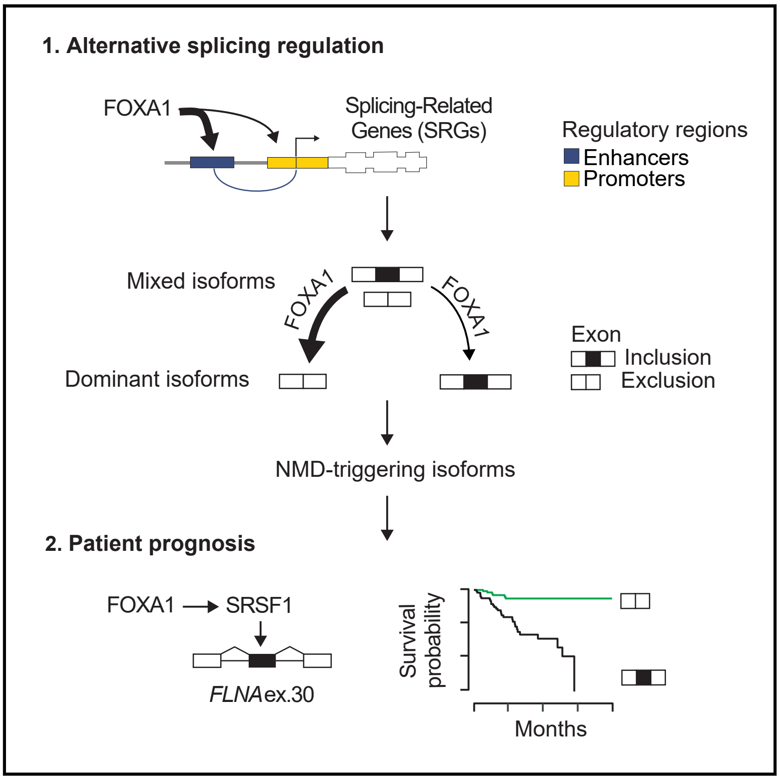

Authors

Marco Del Giudice, John G. Foster, Serena Peirone, ..., Giuseppe Basso, Prabhakar Rajan, Matteo Cereda

Correspondence

p.rajan@qmul.ac.uk (P.R.), matteo.cereda1@unimi.it (M.C.)

In brief

Del Giudice et al. identify the pioneer transcription factor FOXA1 as a master regulator of alternative splicing in prostate cancer. By controlling splicing factors, FOXA1 buffers the noise of isoform production toward a mRNA dominant product. This regulation impacts on splicing of nonsense-mediated decay-determinant exons influencing patient survival.

Highlights

- FOXA1 is a master transcriptional regulator of splicing factors in prostate cancer
- FOXA1 drives splice isoform production toward an optimal dominant mRNA product
- FOXA1 controls exons triggering NMD, influencing prostate cancer patient prognosis
- FOXA1-controlled SRSF1 enhances inclusion of FLNA exon 30, promoting disease recurrence

Del Giudice et al., 2022, Cell Reports *40*, 111404
September 27, 2022 © 2022 The Author(s).
https://doi.org/10.1016/j.celrep.2022.111404

Cell Reports

CellPress
OPEN ACCESS

Article

FOXA1 regulates alternative splicing in prostate cancer

Marco Del Giudice,[1,2,10] John G. Foster,[3,10] Serena Peirone,[1,4] Alberto Rissone,[1,2] Livia Caizzi,[1,2] Federica Gaudino,[1,2] Caterina Parlato,[1,2] Francesca Anselmi,[1,5] Rebecca Arkell,[3] Simonetta Guarrera,[1,2] Salvatore Oliviero,[1,5] Giuseppe Basso,[1,2] Prabhakar Rajan,[3,6,7,8,9,11,*] and Matteo Cereda[1,4,11,12,*]

[1]Italian Institute for Genomic Medicine, c/o IRCCS, Str. Prov. le 142, km 3.95, 10060 Candiolo (TO), Italy

[2]Candiolo Cancer Institute, FPO—IRCCS, Str. Prov. le 142, km 3.95, 10060 Candiolo (TO), Italy

[3]Centre for Cancer Cell and Molecular Biology, Barts Cancer Institute, Cancer Research UK Barts Centre, Queen Mary University of London, Charterhouse Square, London EC1M 6BQ, UK

[4]Department of Biosciences, Università degli Studi di Milano, Via Celoria 26, 20133 Milan, Italy

[5]Department of Life Science and System Biology, Università degli Studi di Torino, via Accademia Albertina 13, 10123 Turin, Italy

[6]Division of Surgery and Interventional Science, University College London, Charles Bell House, 3 Road Floor, 43–45 Foley Street, London W1W 7TS, UK

[7]The Alan Turing Institute, British Library, 96 Euston Road, London NW1 2DB, UK

[8]Department of Urology, Barts Health NHS Trust, the Royal London Hospital, Whitechapel Road, London E1 1BB, UK

[9]Department of Uro-oncology, University College London NHS Foundation Trust, 47 Wimpole Street, London W1G 8SE, UK

[10]These authors contributed equally

[11]Senior author

[12]Lead contact

*Correspondence: p.rajan@qmul.ac.uk (P.R.), matteo.cereda1@unimi.it (M.C.)

https://doi.org/10.1016/j.celrep.2022.111404

SUMMARY

Dysregulation of alternative splicing in prostate cancer is linked to transcriptional programs activated by AR, ERG, FOXA1, and MYC. Here, we show that FOXA1 functions as the primary orchestrator of alternative splicing dysregulation across 500 primary and metastatic prostate cancer transcriptomes. We demonstrate that FOXA1 binds to the regulatory regions of splicing-related genes, including *HNRNPK* and *SRSF1*. By controlling *trans*-acting factor expression, FOXA1 exploits an "exon definition" mechanism calibrating alternative splicing toward dominant isoform production. This regulation especially impacts splicing factors themselves and leads to a reduction of nonsense-mediated decay (NMD)-targeted isoforms. Inclusion of the NMD-determinant *FLNA* exon 30 by FOXA1-controlled oncogene SRSF1 promotes cell growth *in vitro* and predicts disease recurrence. Overall, we report a role for FOXA1 in rewiring the alternative splicing landscape in prostate cancer through a cascade of events from chromatin access, to splicing factor regulation, and, finally, to alternative splicing of exons influencing patient survival.

INTRODUCTION

Pre-mRNA alternative splicing (AS) is a fundamental genetic process underpinning eukaryotic proteome diversity. AS is the selective inclusion of exons or introns into mature transcripts. Catalyzed by the macromolecular spliceosome complex comprising core spliceosomal factors, AS is finely regulated by auxiliary RNA-binding proteins (RBPs), which bind to sequence-specific nucleotide motifs to promote or repress a given splicing event (Cereda et al., 2014; Van Nostrand et al., 2020a). Genomic studies have also shown that somatic cells exploit RBP-mRNA interactions to promote tumor onset and progression (Pereira et al., 2017; Wang et al., 2018).

AS can be affected by somatic alterations leading to dysregulated expression of splicing-related genes (SRGs) (Sebestyén et al., 2016; Seiler et al., 2018). These alterations have uncovered novel cancer therapeutic targets (Lee and Abdel-Wahab, 2016). Small-molecule compounds targeting RBP-mRNA perturbations have entered clinical trials (Bonnal et al., 2020). For instance, pladienolide B derivatives inhibiting the SF3b splicing commitment complex have efficacy for blood and solid cancers (Zhang et al., 2020; Zhou et al., 2020). Similarly, antisense decoy oligonucleotides targeting RBPs have proven effective in preventing the activation of RBP-driven oncogenic programs (Denichenko et al., 2019). Finally, dysregulated AS has the potential to generate neo-epitopes to a greater extent than point mutations, thus potentially expanding the indications for immunotherapies (Frankiw et al., 2019; Kahles et al., 2018).

The commonest cause of male-specific cancer death is prostate cancer (PC) (Rebello et al., 2021). Despite advances in the diagnosis and treatment of early disease, there are few therapeutic options for end-stage metastatic castration-resistant PC (mCRPC) (Rebello et al., 2021). The disease is difficult to tackle in part due to considerable phenotypic heterogeneity, underpinned

This is an open access article under the CC BY license (http://creativecommons.org/licenses/by/4.0/).

by genomic alterations within different oncogenes or tumor suppressors. These impact on transcriptional and translational programs that are fundamental for the cell in complex ways (Rebello et al., 2021).

Interestingly, aberrant splicing can contribute to the heterogeneous phenotypes of PC (Paschalis et al., 2018; Rajan et al., 2009). The dysregulation of this mechanism increases with disease aggressiveness toward metastatic disease, with most SRGs being transcriptionally dysregulated throughout PC progression (Zhang et al., 2020). Consequently, the AS landscape fingerprints the spectrum of PC disease states, with many aberrant events associated with oncogenic signals driven by transcription factors (TFs), such as MYC and AR (Phillips et al., 2020; Shah et al., 2020). Consistently, novel therapeutic targeting of highly expressed SRGs (specifically members of the SF3 splicing commitment complex) has been shown to have antiproliferative effects in PC models (Kawamura et al., 2019; Zhang et al., 2020).

In the heterogeneous genetic landscape of PC, the only recurrent activating alterations occur within key oncogenic TFs: *AR*, *ERG*, *FOXA1*, and *MYC* (Rebello et al., 2021). Ligand-dependent activation of AR controls a tumorigenic cistrome of androgen-sensitive genes (Pomerantz et al., 2015). FOXA1 is a pioneer TF that reprograms the AR cistrome to drive PC initiation and progression to metastasis (Parolia et al., 2019). In the aggressive neuroendocrine PC (NEPC) subtype, where AR transcription is absent, FOXA1 is essential for proliferation (Baca et al., 2021). Similarly, overexpression of ERG redirects AR and FOXA1 binding to drive invasive PC, illustrating the cooperation between these TFs (Chen et al., 2013; Kron et al., 2017). Finally, aggressive PC is characterized by amplification of *MYC*, which is the most frequent genomic alteration in NEPCs (Rebello et al., 2021). MYC antagonizes AR transcriptional programs pioneered by FOXA1, underscoring the interdependence of PC on this handful of TFs (Hawksworth et al., 2010; Qiu et al., 2021).

Of these four TFs, all but FOXA1, have each been implicated in controlling splicing outcomes in PC by modulating SRG expression or influencing inclusion levels of functionally relevant exons (Phillips et al., 2020; Saulnier et al., 2021; Shah et al., 2020). These studies highlight the involvement of distinct TFs in the dysregulation of AS during PC progression. Nevertheless, in the context of PC transcriptional reprogramming cooperatively driven by these TFs, the magnitude of influence exerted by each individual TF to aberrant AS remains to be elucidated. Here, we systematically assess the impact of the four TFs on AS in primary PC and mCRPC patients.

RESULTS

FOXA1 drives SRG dysregulation in PC by directly binding cognate regulatory regions

To assess the influence individually exerted by AR, ERG, FOXA1, and MYC to the dysregulation of AS in PC, we measured the contribution of their expression to the overall transcription of 148 SRGs. We used available RNA sequencing (RNA-seq) data of 409 primary PCs (Network Cancer, 2015), 118 mCRPCs (Robinson et al., 2015), and 15 NEPCs (Beltran et al., 2016). For our quantitative analysis, we implemented a multivariable covariance approach (1) fitting SRG cumulative expression as a function of TF expression levels using a generalized linear regression and (2) measuring their relative contribution in the model (see STAR Methods). We found that, of the four, *FOXA1* was the strongest positive predictor of SRG cumulative expression in all datasets (Figures 1A and S1A), suggesting that splicing regulation in PC involves a pioneer TF.

We next sought to systematically investigate the three-dimensional architectural features of transcriptional control by FOXA1 in PC in the context of the other TFs (Figure S1B). To do so, we integrated information on chromatin interactions in PC cell lines and accessibility in primary PCs. Firstly, we identified TF binding sites in VCaP and LNCaP cell lines from chromatin immunoprecipitation sequencing (ChIP-seq) experiments. We merged peak calls by cell line to define the cell-line-specific TF binding regions. Secondly, we exploited results of chromatin interaction analysis by paired-end tag sequencing experiments in the same cell lines and identified proximal enhancer-gene associations (i.e., $\leq$1 mega base pair [Mbp]). We then selected TF binding sites and enhancer-gene associations that were present in actively transcribed regions of primary PC from 26 Assay for Transposase-Accessible Chromatin using sequencing experiments. Finally, we defined gene promoters (i.e., ±2,000 bp) and cognate proximal enhancer regions with TF-specific binding sites as active TF-bound regulatory regions of PC.

To identify the biological processes under the direct transcriptional control of each TF, we assessed the over-representation of genes with active TF-bound regions in a list of 186 KEGG canonical pathways. Overall, the spliceosome pathway had the highest enrichment of genes with active TF-bound promoter and enhancer regions across VCaP- and LNCaP-based datasets (Figure S1C). Of the TFs, we found the most prevalent contribution of FOXA1 on regulatory regions of spliceosome genes across conditions, with the strongest involvement on proximal enhancers (Figure 1B). These results corroborate the known contribution of AR, ERG, and MYC in AS regulation, while importantly revealing the broadest influence of FOXA1 on the transcriptional control of spliceosome genes compared with the other TFs.

To identify physiologically relevant candidate SRGs controlled by FOXA1, we stratified primary PC and mCRPC RNA-seq data according to *FOXA1* expression and performed differential gene expression analyses (Figures S2A–S2C). We identified 71 SRGs that were differentially expressed by *FOXA1* in either dataset (Figure 1C). Of these, 90% harbored active FOXA1 binding sites in regulatory regions, demonstrating a direct transcriptional control by this pioneer factor. Consistent with a known tendency to occupy distal regulatory elements (Ramanand et al., 2020), we found that FOXA1 preferentially bound enhancer, over promoter, regions of differentially expressed SRGs (Figure 1C). To further investigate this regulation, we performed RNA-seq on FOXA1 siRNA-treated and control samples from AR-dependent (AR^+) VCaP and AR-independent (AR^-) PC3 cell lines (Figures S2D–S2G). We found that 18 FOXA1-regulated and -bound SRGs were differentially expressed by FOXA1 in both cell lines regardless of AR status (Figures 1C, 1D, S2H, and S2I). We refer to these AR-independent FOXA1-regulated SRGs, hereafter FOXA1-controlled SRGs.

CellPress
OPEN ACCESS

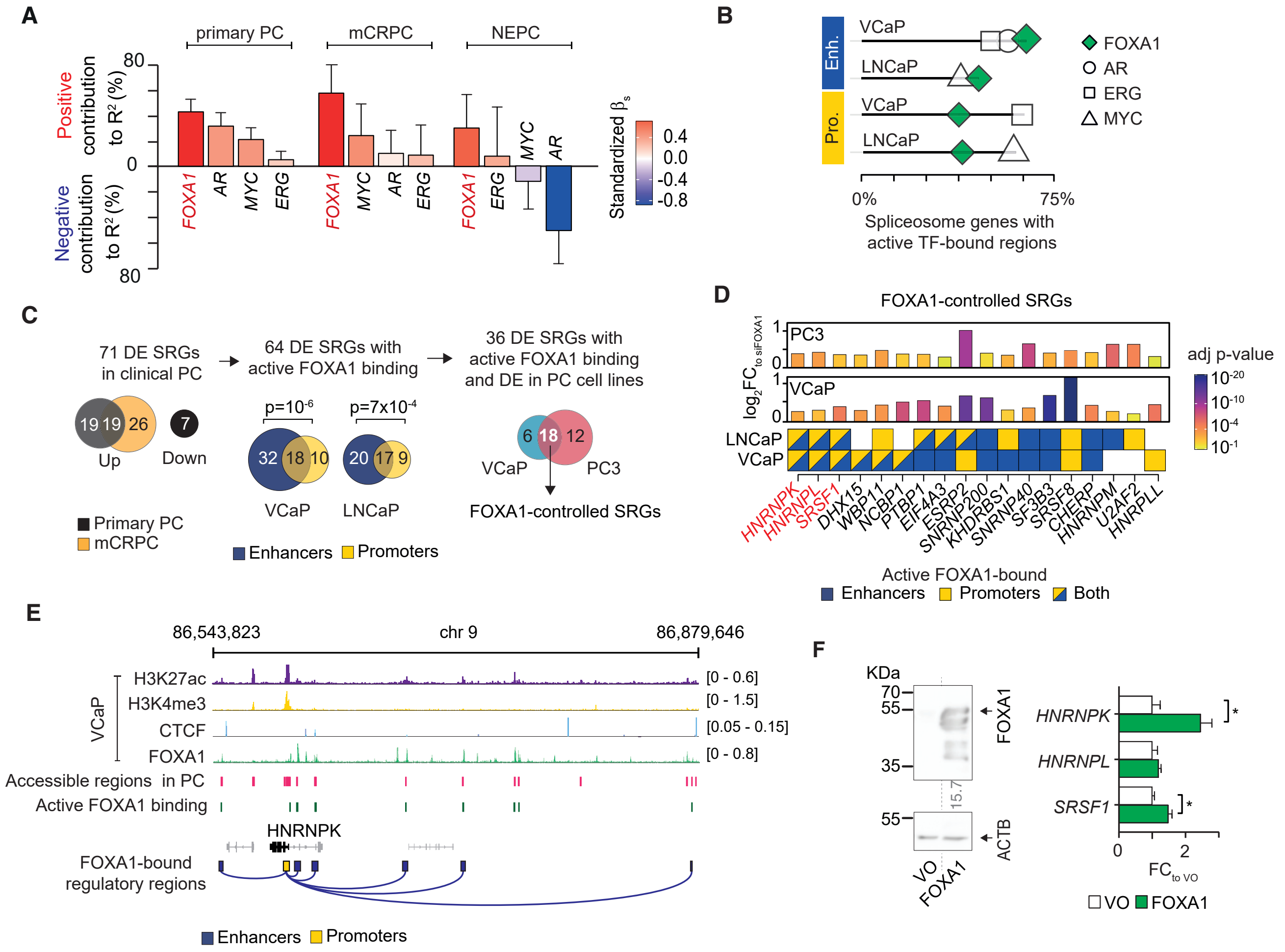

Figure 1. FOXA1 transcriptionally controls splicing-related genes in PC

(A) Results of multivariable covariance analysis between the cumulative expression of SRGs and the expression of TFs in primary PCs, mCRPC, and NEPC. Color key indicates the standardized β coefficients of the model.

(B) Enrichment of spliceosome genes with active TF binding sites within chromatin-accessible promoters (yellow) and enhancers (blue) for the VCaP- and LNCaP-based architectural datasets. The fraction of spliceosome genes with active TF-bound regions for each TF is shown.

(C) Framework used to select FOXA1-controlled SRGs. p values refer to a two-tailed test of equal proportion comparing the proportion of active FOXA1 binding sites on SRG promoters (yellow) and enhancers (blue). DE, differentially expressed.

(D) Bar plots indicate fold change (FC) in expression levels of FOXA1-controlled SRGs upon FOXA1 depletion in VCaP and PC3 cells. Color code indicates DEseq2 adjusted p value. Bottom annotations depict the active FOXA1-bound regulatory regions for each SRG.

(E) ChIP-seq density read tracks of H3K27ac, H3K4me3, CTCF (two overlayed experiments) and FOXA1 (five overlayed experiments) in VCaP cells are shown together with recurrent accessible regions of primary PC from assay for transposase-accessible chromatin using sequencing experiments, active FOXA1 binding sites and RNA PolII chromatin interaction analysis by paired-end tag sequencing-derived FOXA1-bound regulatory regions.

(F) Representative western blotting images (left panel) of whole-cell lysates from PC3 cells transfected with 2 μg of plasmid DNA vectors encoding FOXA1 or vector only (VO) control using antibodies to FOXA1 and ACTB. ACTB-normalized mean fold change in protein expression compared with control are shown below the upper blot image. Bar plots (right panel) depict the mean fold change in expression of candidate SRGs measured by qRT-PCR upon FOXA1 overexpression (biological triplicates). Error bars correspond to standard error of the mean. Two-tailed t test was used to compare conditions (*p ≤ 0.05).

Of these, *HNRNPK*, *HNRNPL*, and *SRSF1* particularly drew our interest as they harbor active FOXA1 binding sites in both promoter and interacting enhancer regions in both VCaP- and LNCaP-based datasets (Figure 1D). To probe the transcriptional architecture of these SRGs, we included ChIP-seq data for H3K27ac (marker of active enhancer), H3K4me3 (marker of active promoter), and CTCF (marker of topologically associating domain boundary element) in the corresponding PC cells. We found that FOXA1 binds to the promoter (marked by H3K4me3) and cognate active enhancers (marked by H3K27ac), within chromatin loops (delimited by CTCF sites) of *HNRNPK* (Figure 1E) and the other two SRGs (Figure S3).

To test the robustness of our results, we profiled the expression of these three FOXA1-controlled SRGs by qRT-PCR on FOXA1 siRNA-treated and control samples from VCaP, PC3, LNCaP, and DU145 cell lines. We observed a significantly

reduced expression of *HNRNPK* and *SRSF1* in the majority of PC cell lines upon FOXA1 depletion (Figures S4A–S4D). Consistently, ectopic expression of FOXA1 protein in PC3 cells resulted in a significant increase in *HNRNPK* and *SRSF1* expression compared with the control (Figure 1F).

Overall, these results clearly demonstrate that FOXA1 directly drives SRG expression, particularly *HNRNPK* and *SRSF1*, by preferentially binding cognate chromatin-accessible active enhancers. The direct transcriptional control of FOXA1 primarily impacts on splicing factors.

FOXA1 calibrates AS in PC, predominantly within SRGs

As we found that FOXA1 primarily controls expression of splicing factors, we next sought to determine its impact on the downstream AS landscape of PC. To do this, we explored the inclusion level of 60,699 alternatively spliced exons in their corresponding transcripts (i.e., percent spliced in [psi or Ψ]) across 384 primary tumors (Kahles et al., 2018). We sought to assess the impact of FOXA1 on AS by quantifying exon inclusion changes, in terms of mean and standard deviation, between tumors with high *FOXA1* expression ($\geq 75^{th}$ percentile of expression distribution) and the remaining ones. To select exons with a significant splicing association with high *FOXA1* expression, we employed two non-parametric statistical tests followed by bootstrapping simulations to control for sample size differences and estimate empirical significance levels (see STAR Methods and Figure S5A). We identified 7,121 AS exons that had significant inclusion changes between tumors with high *FOXA1* expression and the remaining ones (i.e., FOXA1-regulated exons). Whereas, 23,318 exons had non-significant inclusion changes upon FOXA1 high expression (i.e., FOXA1-unregulated exons).

Exons can be concomitantly included and excluded in different transcripts from the same gene leading to populations of mixed isoforms (Ψ = 0.5) or dominant isoforms (Ψ = 0 or 1) (Agirre et al., 2021). To gain insights into rewiring of the AS landscape by FOXA1 in this light, we examined the trajectory of inclusion changes driven by high *FOXA1* expression in terms of their mean and standard deviation across primary PCs (i.e., $\Delta\mu(\Psi)$ and $\Delta\sigma(\Psi)$, respectively, Figure 2A). To do so, we measured the cumulative distributions of positive and negative splicing changes (i.e., $\Delta\mu(\Psi)$ and $\Delta\sigma(\Psi)$) starting from the mean inclusion level of 0.5 (i.e., mixed isoform population) to the boundaries of 0 and 1 (i.e., dominant isoform population). As a reference, we calculated the empirical distribution of the expected number of exons with splicing changes ranging from mixed to dominant isoform populations based on the assumption of an equal probability of positive and negative changes (see STAR Methods). Lowly included events were inhibited across tumors with high *FOXA1* expression compared with remaining ones, whereas highly included events were enhanced by FOXA1 (Figure 2B, left panel). Concomitantly, exons were more uniformly spliced across tumors with high *FOXA1* expression than remaining ones (Figure 2B, right panel). For a quantitative analysis of this phenomenon, we stratified FOXA1-regulated events into four groups according to three inclusion cutoffs (i.e., $\mu(\Psi)_{primary\ PC}$ = 0.15, 0.50, and 0.85). For each group, we compared the proportion of events with positive and negative $\Delta\mu(\Psi)$ and $\Delta\sigma(\Psi)$. Lowly included exons ($\mu(\Psi)_{primary\ PC}$ < 0.15) were significantly FOXA1 inhibited, whereas mid or highly included exons ($\mu(\Psi)_{primary\ PC}$>0.5) were significantly enhanced by FOXA1 (Figure 2B, left panel). Furthermore, exons were significantly uniformly spliced across tumors with high *FOXA1* expression (blue bars) regardless of their inclusion levels (two-tailed exact binomial test $p < 10^{-3}$; Figure 2B, right panel). Together, these results indicate that FOXA1 lessens the noise of isoform production toward a precise equilibrium, in a consistent way across primary tumors, thereby promoting the assembly of dominant isoforms in PC.

To test this finding, we identified differentially alternatively spliced exons by FOXA1 from our RNA-seq data in VCaP and PC3 cells. We stratified these exons into the four groups of exon inclusion (see above) and compared the proportion of FOXA1-inhibited and -enhanced exons in each group. We found that lowly included exons were significantly FOXA1 inhibited in both cell lines (Figures 2C and 2D). These data confirm the calibrating effect of FOXA1 on the AS equilibrium of PC, with a prominent role for FOXA1 in inhibiting lowly included AS events.

Finally, to characterize the impact of FOXA1-mediated AS regulation on fundamental biological processes, we performed over-representation analysis of genes harboring FOXA1-regulated AS events in primary tumors and cell lines. Out of 186 canonical KEGG pathways, the spliceosome gene set was the top-ranked affected process in all datasets (Figure 2E) and by AS event category (Figure S5B). These results suggest that FOXA1 significantly impacts on AS of splicing factors and not just their expression.

Overall, our comprehensive analysis demonstrates that FOXA1 calibrates AS toward an equilibrium further promoting the assembly of dominant isoforms in PC. This phenomenon is particularly evident for splicing factors.

FOXA1 controls the inclusion of NMD-determinant exons

Splicing factors can regulate their own mRNAs by controlling the inclusion of nonsense mediated decay (NMD)-determinant exons (Kurosaki et al., 2019). By selectively including premature termination codon (PTC)-introducing and PTC-preventing exons, these transcripts can be targeted for degradation by NMD (Figure 3A). Therefore, we sought to assess the regulation of NMD-determinant exons by FOXA1. Using a list of 15,518 NMD-determinant cassette exons (CEs) (Pervouchine et al., 2019), we found a significant enrichment of this class of exons among FOXA1-regulated exons (Figure 3B). By inspecting the distribution of mean inclusion changes in tumors with high *FOXA1* expression compared with remaining ones, we found that FOXA1-regulated PTC-introducing CEs were significantly inhibited, whereas PTC-preventing events were significantly enhanced compared with controls (Figure 3C). These results suggest that FOXA1 predominantly calibrates AS toward dominant isoforms that escape NMD.

By employing RNA-seq data from FOXA1-depleted VCaP and PC3 cells, we confirmed that PTC-introducing exons were significantly inhibited by FOXA1 relative to controls in both cell lines (Figure S5C). This was especially true for NMD-determinant exons in SRGs. Specifically, PTC-introducing exons were enriched for FOXA1-inhibited exons (Figure 3D, bottom-left

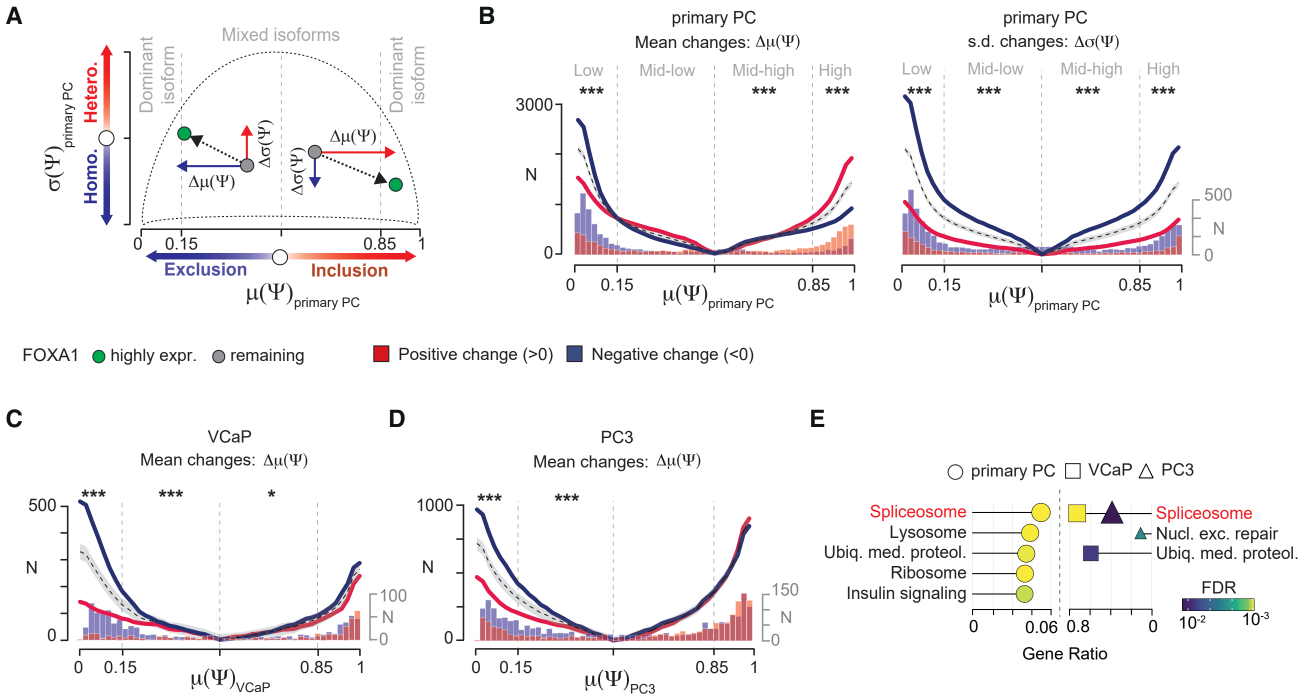

Figure 2. FOXA1 calibrates the alternative splicing equilibrium of PC by enhancing the production of dominant isoforms

(A) Overview of alternatively spliced exon trajectories in the space defined by mean and standard deviation (SD) of exon inclusion levels (Ψs). Color codes indicate positive (red) and negative (blue) changes of mean and SD of Ψs between FOXA1 highly expressing tumors and remaining ones.

(B) Cumulative distribution plots depict the number (N) of exons with either positive (red) or negative (blue) changes ranging from $\mu(\Psi)_{\text{primary PC}}$ of 0.5 (i.e., mixed isoforms) to the boundaries of 0 and 1 (i.e., dominant isoforms). Dashed lines represent the expected mean cumulative distribution of events with inclusion changes generated by 1,000 Monte Carlo simulations. Gray area represents confidence intervals (5%–95%). Histograms of the number of exons with positive and negative changes are superimposed on the x axis. On left panel, a preponderance of blue over red indicates that FOXA1 mostly inhibits exon inclusion, whereas the dominance of red compared with blue indicates a major enhancement of exon inclusion by FOXA1. On right panel, a preponderance of blue over red indicates that exons were more uniformly spliced across tumors by FOXA1, whereas the dominance of red compared with blue indicates more heterogeneous inclusion upon high *FOXA1* expression.

(C and D) Cumulative distribution plots depict differentially alternatively spliced events (N) with positive (red) and negative (blue) mean inclusion changes upon FOXA1 depletion in VCaP (C) and PC3 (D) cells ranging from mixed (i.e., $\mu(\Psi) = 0.5$) to dominant (i.e., $\mu(\Psi) = \{0,1\}$) isoform population. Histograms of the number of exons with positive and negative changes are superimposed on the x axis. A preponderance of blue over red indicates that FOXA1 mostly inhibits exon inclusion.

(E) Over representation analysis performed on genes harboring FOXA1-regulated AS events in primary PCs and cell lines. Shape size and gene ratio indicate the number (from 12 to 59) and the fraction of selected genes in each pathway, respectively. Color key represents the statistical significance (FDR) of the enrichment. Only top 5 enriched pathways (FDR < 0.1), if any, are shown and sorted by statistical significance. For (B–D), stars indicate the significance of two-tailed exact binomial tests comparing the abundances of exons with positive and negative changes against a null hypothesis with probability = 0.5 in four groups of Ψs. $^{**}p < 10^{-2}$ and $^{***}p < 10^{-3}$.

quadrant), whereas PTC-preventing exons were predominantly FOXA1-enhanced exons (Figure 3D, top-right quadrant).

Overall, these results indicate that the enhancement of dominant isoform production by FOXA1 includes those that escape NMD, particularly in splicing factors.

FOXA1 mediates exon silencing by controlling *trans*-acting factors within an "exon definition" mechanism

Alternatively spliced CEs have weaker splice sites (ss), are strongly conserved during evolution, and are usually shorter with longer flanking introns (Keren et al., 2010; Mazin et al., 2021). Therefore, we sought to delineate the features of FOXA1-mediated exon definition in primary PC. By performing conventional ss strength analysis, we did not find any significant difference in ss scores between FOXA1-regulated and -unregulated exons (Figure S5D). However, compared with FOXA1-unregulated events, FOXA1-regulated exons were (1) significantly shorter with longer flanking introns (Figure 4A) and (2) more conserved across 100 species, especially within 100 nt of the exon/intron junctions (Figure 4B). The stronger evolutionary constraint on FOXA1-regulated exons suggests functionality. These results indicate that FOXA1-mediated exon definition depends on exon length and conservation, demonstrating a model in which FOXA1 controls exons in *trans*.

Furthermore, splicing is a co-transcriptional process in which chromatin modifications can impact on recruitment of splicing factors to the pre-mRNA of a minority of exons to enhance their definition (Agirre et al., 2021). To investigate chromatin involvement in FOXA1-mediated exon definition, we collected 876 CEs marked by combinations of histone modifications and measured their over-representation within FOXA1-regulated exons relative to unregulated events. We found that a minority

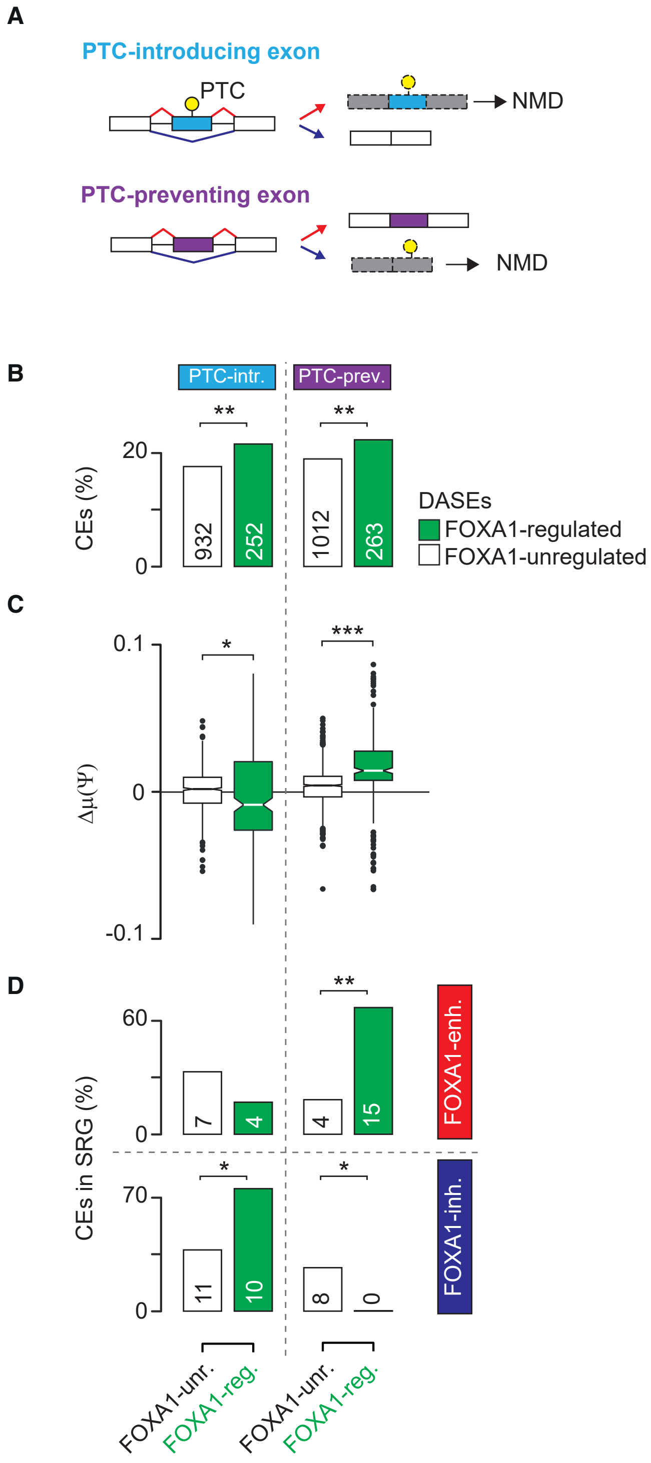

Figure 3. FOXA1 controls nonsense-mediated decay determinant exons

(A) Overview of selective inclusion of premature termination codon (PTC) introducing, or preventing, CEs triggering NMD.

(B) Bar plots show the proportion of PTC-introducing and PTC-preventing exons among FOXA1-regulated and FOXA1-unregulated exons. Numbers of exons in each category are indicated.

(C) Distribution of mean inclusion changes of NMD-determinant FOXA1-regulated and FOXA1-unregulated exons.

(D) Bar plots show the proportion of PTC-introducing and PTC-preventing exons among FOXA1-regulated and FOXA1-unregulated exons. Exons are stratified according to their positive (red) and negative (blue) mean inclusion change upon high expression of *FOXA1*. The number of exons in each category is indicated. Stars indicate statistical significance of two-tailed Fisher's exact test (B and D) and Wilcoxon rank-sum test (C). *$p < 0.05$, **$p < 10^{-2}$, ***$p < 10^{-3}$.

of FOXA1-regulated exons were significantly enriched for splicing-associated chromatin signatures (SACS; Figure 4C) compared with FOXA1-unregulated events, particularly for SACS marking generally excluded exons (i.e., SACS 4, 5, and 7; Figure 4C, two-tailed Fisher's exact test, $p = 7.6 \times 10^{-6}$). These findings suggest that chromatin modifications may also contribute to FOXA1-mediated exon regulation for a subset of events.

To gain insights into *trans*-acting regulation of FOXA1-mediated AS, we performed a position-dependent analysis of *cis*-acting sequences, which define splicing regulation by *trans*-acting factors. To do so, we integrated our conventional RNA motifs analysis (Cereda et al., 2014) with RBP binding data and associated *cis*-acting sequences to cognate *trans*-acting factors (see STAR Methods). In brief, we searched for clusters of tetramers that were enriched at specific positions around FOXA1-regulated exons compared with unregulated events. Next, in light of the reproducibility of splicing factor binding positions across cell types (Van Nostrand et al., 2020b), we searched for RBP crosslinking sites from eCLIP experiments in HepG2 cells at FOXA1-regulated exons with tetramer instances. Finally, we associated tetramers to cognate RBPs on similarity of (1) their sequence with canonical RBP consensus motifs and (2) position-dependent representation of their occurrences (i.e., splicing maps) with those of RBP crosslinking sites at exon-intron junctions.

We identified 13 tetramers enriched at FOXA1-regulated exons (Figure 4D) and associated with 10 FOXA1-regulated SRGs (Figure 4E). The majority of tetramers (77%) were enriched at FOXA1-inhibited exons, corroborating the propensity for an extensive FOXA1-mediated exon silencing. In particular, T-rich tetramers were strongly enriched at the 3' ss of FOXA1-inhibited exons (Figure 4D). These motifs were associated with RBPs that canonically bind within the upstream intron, predominantly FOXA1-controlled proteins PTBP1, U2AF2, HNRNPC, and HNRNPK (Figure 4E).

Together, our data describe the FOXA1-mediated splicing code in primary PC where different *trans*-acting splicing factors control exon inclusion. In particular, FOXA1-mediated exon silencing appears to preferentially rely on splicing repressors acting at the 3' ss, which are directly controlled by FOXA1.

FOXA1-regulated NMD-determinant exons impact on PC patient survival

In light of recent evidence implicating PTC-introducing exons in lung cancer disease-free survival (Thomas et al., 2020), we sought to investigate whether the subset of FOXA1-regulated NMD-determinant exons could impact PC patient prognosis.

CellPress
OPEN ACCESS

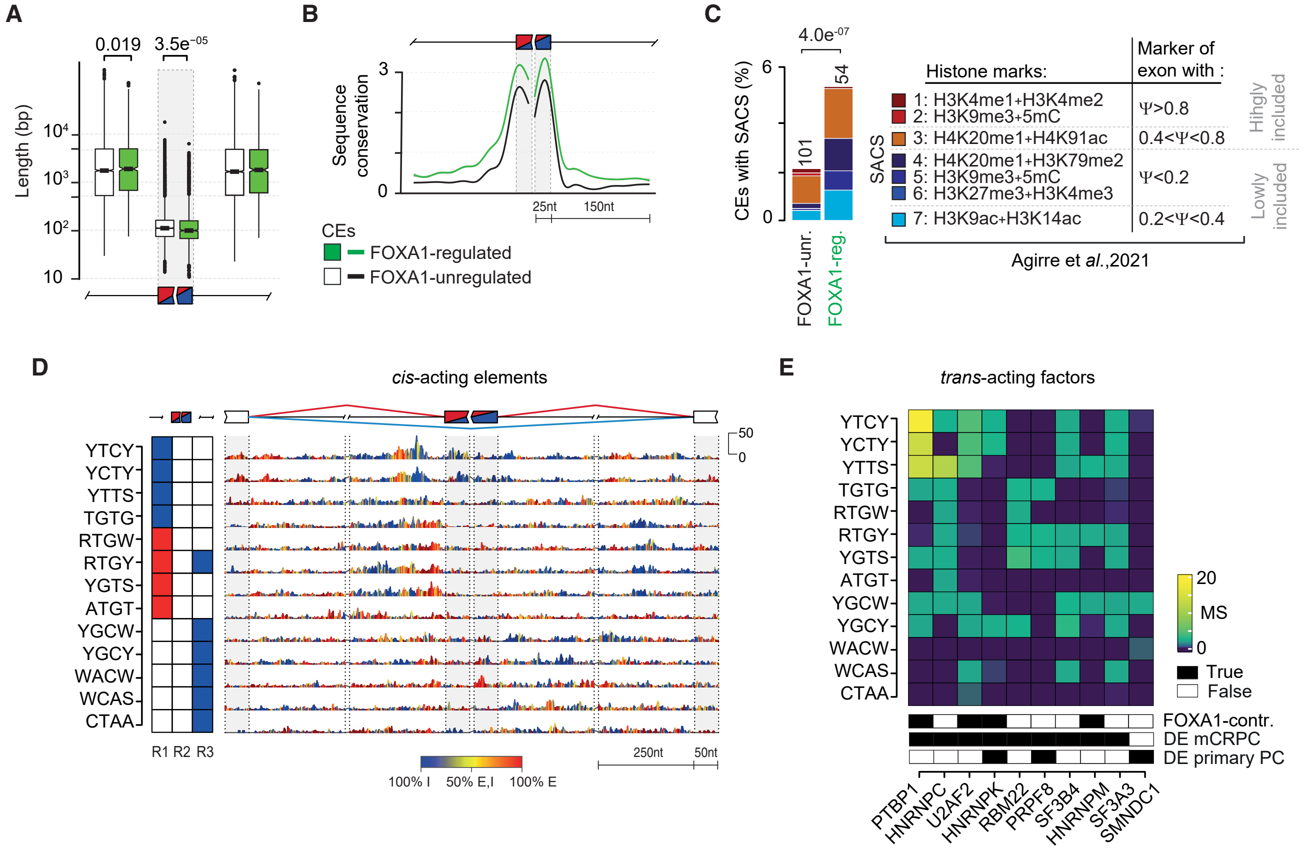

Figure 4. FOXA1 mediates exon silencing by controlling *trans*-acting factors within an exon definition mechanism

(A) Length distributions of exon and flanking introns for FOXA1-regulated and -unregulated cassette exons. p values of two-tailed Wilcoxon rank-sum test are reported if significant.

(B) Distribution of smoothed conservation scores (PhyloP, 100 vertebrates) of FOXA1-regulated and -unregulated exons in exonic and flanking intronic regions.

(C) Bar plots show the fraction of SACS marked exons in FOXA1-regulated and FOXA1-unregulated exons (left panel). Color indicates SACS type. Corresponding histone modifications and categories of marked exons are reported as described in Agirre et al. (2021).

(D) RNA splicing map of multivalent RNA motifs enriched at FOXA1-regulated exons. Left color-coded panel indicates the regions at exon/intron junctions where motifs were enriched at inhibited (blue) or enhanced (red) exons. The right panel depicts the nucleotide-resolution RNA splicing map of each motif at the FOXA1-regulated exons, and their flanking exons. The color key indicates whether the position-specific contribution originates from enhanced (E) (red), inhibited (I) (blue), or both (yellow) sets. Maximum RNA motifs enrichment score of the top tetramer, which is used for all tetramers, is reported on the right. nt, nucleotides.

(E) Heatmap shows the association between enriched multivalent RNA motifs and cognate SRGs that were differentially expressed in primary PCs or mCRPCs in terms of matching score (MS).

To do so, we firstly divided FOXA1-regulated NMD-determinant CEs into four groups based on $\Delta\mu(\Psi)$ (Figure S5F). We then stratified 332 primary PC patients with available clinical data according to low and high cumulative event inclusion of each group (see STAR Methods). Of these groups, univariate Cox proportional hazard models revealed that a low cumulative inclusion of FOXA1-inhibited PTC-introducing exons was significantly associated with a longer patient survival relative to high inclusion (Figure 5A, upper left panel). Similarly, a high cumulative inclusion of FOXA1-enhanced PTC-preventing exons was significantly associated with a better prognosis than low cumulative inclusion (Figure 5A, bottom right panel).

Secondly, to determine the impact of each individual NMD-determinant exon on patient survival, we again used a univariate Cox proportional hazard model to calculate the hazard ratio (HR) associated with exon inclusion. Overall, 85 exons were associated with survival (i.e., two-tailed log rank test $p < 0.05$). Most of the exons associated with poor prognosis (62%, n = 24, HR > 1, i.e., "harmful") were FOXA1-inhibited PTC-introducing CEs (Figure 5B, top quadrants). Conversely, exons associated with favorable prognosis (HR < 1, i.e., "favorable") were mostly FOXA1-enhanced PTC-preventing exons (61%, n = 28; Figure 5B, bottom quadrants). Together, these results suggest that FOXA1-mediated AS of NMD-determinant exons predominantly results in a positive patient survival by silencing harmful PTC-introducing exons and enhancing the inclusion of favorable PTC-preventing ones.

However, of eight most prognostic exons, six were harmful (i.e., FDR < 0.05; Figure 5C). Four exons were inhibited by FOXA1, whereas exons in *FLNA* and *NDGR1* were enhanced. To evaluate which of these exons exhibited the greatest link with *FOXA1* expression, we employed our multivariable

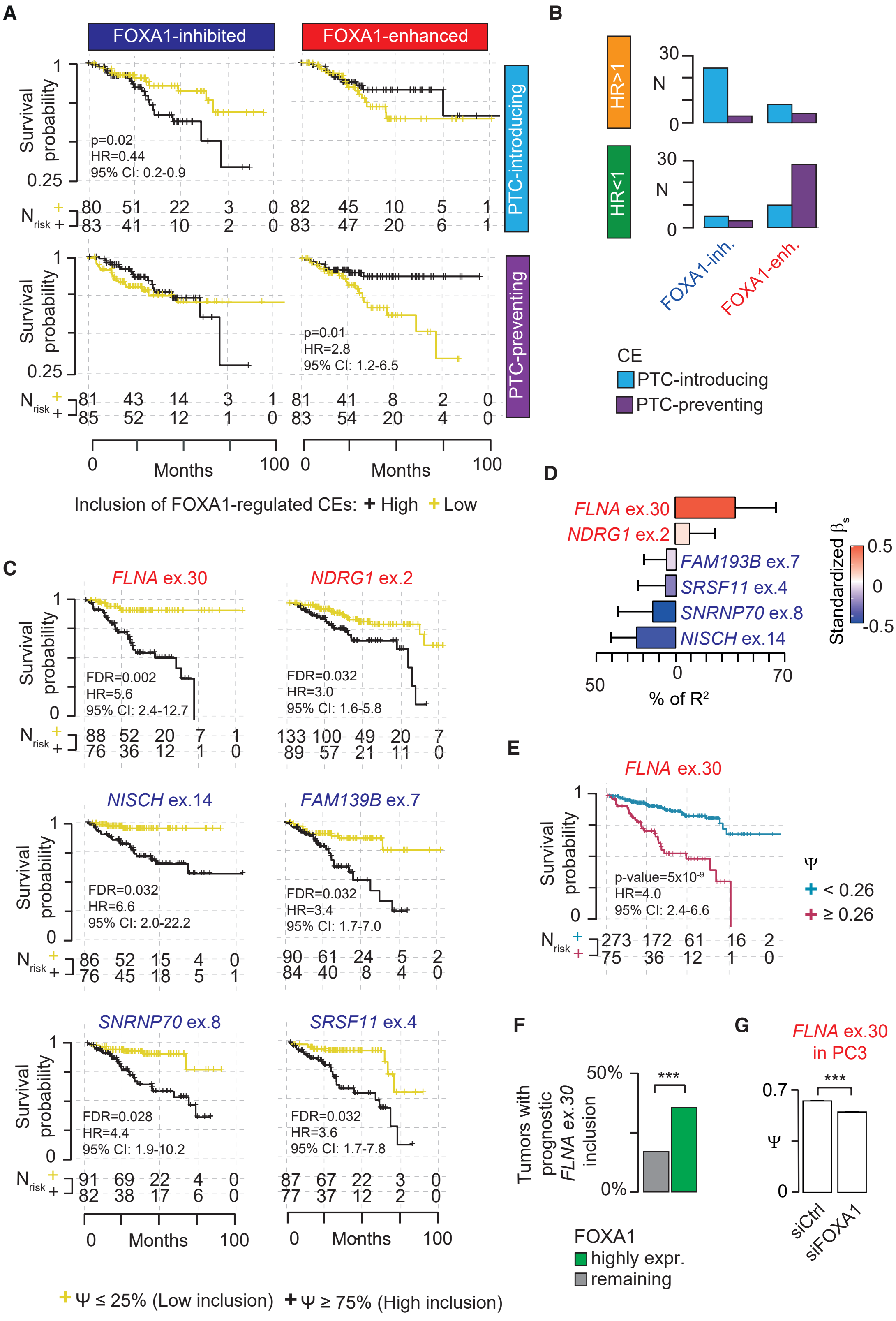

(legend on next page)

covariance analysis (see STAR Methods). Among all events, *FLNA* exon 30 inclusion levels showed the strongest positive contribution to the overall correlation with *FOXA1* expression (Figures 5D and S5G). Indeed, *FLNA* exon 30 inclusion was significantly higher in tumors with high FOXA1 expression than remaining ones (Figure S5H).

Therefore, we sought to determine whether primary PCs with a prognostic inclusion level of *FLNA* exon 30 also exhibit high *FOXA1* expression. Using the maximally selected rank statistics approach (Lauria et al., 2020; Lausen and Schumacher, 1992), we identified a *FLNA* exon 30 ($\Psi = 0.26$) as the optimal cutpoint defining primary PC patient prognosis (Figures 5E and S5I). By stratifying patients on this cutpoint, we observed a larger proportion of high *FOXA1*-expressing tumors with prognostic inclusion level of *FLNA* exon 30 than remaining ones (Figure 5F). This result corroborates the link between high *FOXA1* expression and high *FLNA* exon 30 inclusion.

Finally, we validated *FLNA* exon 30 inclusion in PC3 cells upon FOXA1 depletion by digital droplet PCR (ddPCR) and endpoint PCR splicing assays and confirmed the dependence of this exon on FOXA1 (Figures 5G and S4E).

Overall, these results reveal that the AS of FOXA1-regulated NMD-determinant exons has a clinically relevant impact on PC recurrence. FOXA1-mediated AS inhibits the majority (75%) of harmful PTC-introducing exons and enhances almost all (90%) favorable PTC-preventing exons. However, FOXA1 also enhances a small subset of NMD-determinant exons, such as *FLNA* exon 30, which predicts disease recurrence, and therefore may drive a more aggressive cancer phenotype.

FLNA exon 30 promotes PC cell growth and is controlled by the FOXA1 target SRSF1

Being the most harmful NMD-determinant exon associated with FOXA1 expression, we sought to investigate the impact of *FLNA* exon 30 on PC cell phenotypes. To do so, we transfected AR^{-} PC3 cells with ectopic expression vectors with and without exon 30 (i.e., FLNA+ex30 and FLNAΔex30, respectively), and confirmed exon 30 expression levels by endpoint PCR splicing assays (Figures S4F and S4G). Using cell viability MTT and survival clonogenic assays, we observed a significant increase in growth and survival, respectively, of cells overexpressing FLNA+ex30 compared with the case for FLNAΔex30 (Figure 6A).

To determine putative regulators of *FLNA* exon 30 inclusion associated with *FOXA1* expression, we performed our multivariable covariance analysis between *FLNA* exon 30 inclusion and the expression levels of ten FOXA1-controlled SRGs (see STAR Methods). *SRSF1*, followed by *HNRNPK*, expression was the strongest positive contributor to the correlation with *FLNA* exon 30 inclusion, while *HNRNPLL* expression showed the greatest association with exon 30 skipping (Figure 6B).

To further evaluate the contribution of *SRSF1* to *FLNA* exon 30 inclusion, in the context of FOXA1, we stratified primary PC samples according to high and low expression of these genes (i.e., 75th and 25th percentile of expression distributions, respectively). We found a significantly higher inclusion of *FLNA* exon 30 in samples with high expression of both *FOXA1* and *SRSF1* compared with other groups of samples (Figure 6C).

We next assessed SRSF1 binding around *FLNA* exon 30 using eCLIP-derived crosslinking information in HepG2 cells. We observed strong binding of SRSF1 in the surrounding exons, consistent with a predominant role of FOXA1-controlled SRSF1 in exon 30 incorporation (Figure 6D). To test this, we performed siRNA-mediated depletion of SRSF1 in PC3 cells (Figure S4H). Using endpoint PCR and ddPCR splicing assays, we measured a significant decrease in *FLNA* exon 30 inclusion in siRNA conditions compared with controls (Figures 6E and 6F).

Taken together, these findings demonstrate that *FLNA* exon 30 inclusion is regulated by SRSF1, which is directly controlled by FOXA1. Increased expression of *FLNA* exon 30 confers a growth advantage to PC cells, which may drive poorer patient prognosis.

DISCUSSION

In this study, by analysis of transcriptomics, protein-mRNA interactions, epigenomics, and chromosome conformation, we reveal that the pioneer TF FOXA1 orchestrates AS regulation in PC impacting on patient survival.

Collectively, our results indicate that *FOXA1* expression is a predominant hallmark of the transcriptional dysregulation of SRGs. As a pioneer factor, FOXA1 opens up nucleosomal domains for DNA binding by distinct TFs (Fei et al., 2019; Lupien et al., 2008). This pliant mechanism (Ramanand et al., 2020) may explain why FOXA1 hallmarks the global SRG dysregulation

Figure 5. FOXA1-regulated NMD-determinant exons predict PC patient prognosis

(A) Kaplan-Meier plots of disease-free survival for primary PC patients stratified according to the 25th and 75th percentile of the cumulative inclusion levels of NMD-determinant exons that are inhibited or enhanced by high *FOXA1* expression. Numbers of patients at risk (N_{risk}) are reported at each time point on the x axis. Univariate HRs with 95% confidence intervals (CI) and two-tailed log rank test p values are shown where statistically significant.

(B) Bar plots show the number of FOXA1-inhibited or -enhanced NMD-determinant exons with a significant harmful (HR > 1, top panel) or favorable (HR < 1, bottom panel) impact on patient disease-free survival (two-tailed log rank test $p < 0.05$).

(C) Kaplan-Meier plots of disease-free survival for primary PC patients with low and high inclusion of the six most prognostic harmful exons (FDR < 0.05). Number of patients at risk (N_{risk}) are reported at each time point on the x axis. Univariate HRs with 95% CI and two-tailed log rank test FDR are shown.

(D) Results of multivariable covariance analysis between *FOXA1* expression and the inclusion levels of the six most prognostic harmful exons. Color key indicates the standardized β coefficients of the model.

(E) Kaplan-Meier plots of disease-free survival for primary PC patients stratified on the optimal *FLNA* exon 30 inclusion level (i.e., $\Psi \geq 0.258$, maximally selected rank statistics = 5.35). Number of patients at risk (N_{risk}) are reported at each time point on the x axis. Univariate HRs with 95% CI and two-tailed log rank test FDR are shown.

(F) Bar plots show the proportions of high FOXA1 expressing and remaining tumors with *FLNA* exon 30 $\Psi \geq 0.258$.

(G) Bar plots show Ψs of *FLNA* exon 30 in PC3 cells measured by ddPCR upon FOXA1 depletion with one siRNA duplex (si1, 40 nM for 72 h). For (F) and (G), two-tailed t test was used to compare conditions: ***$p < 0.001$.

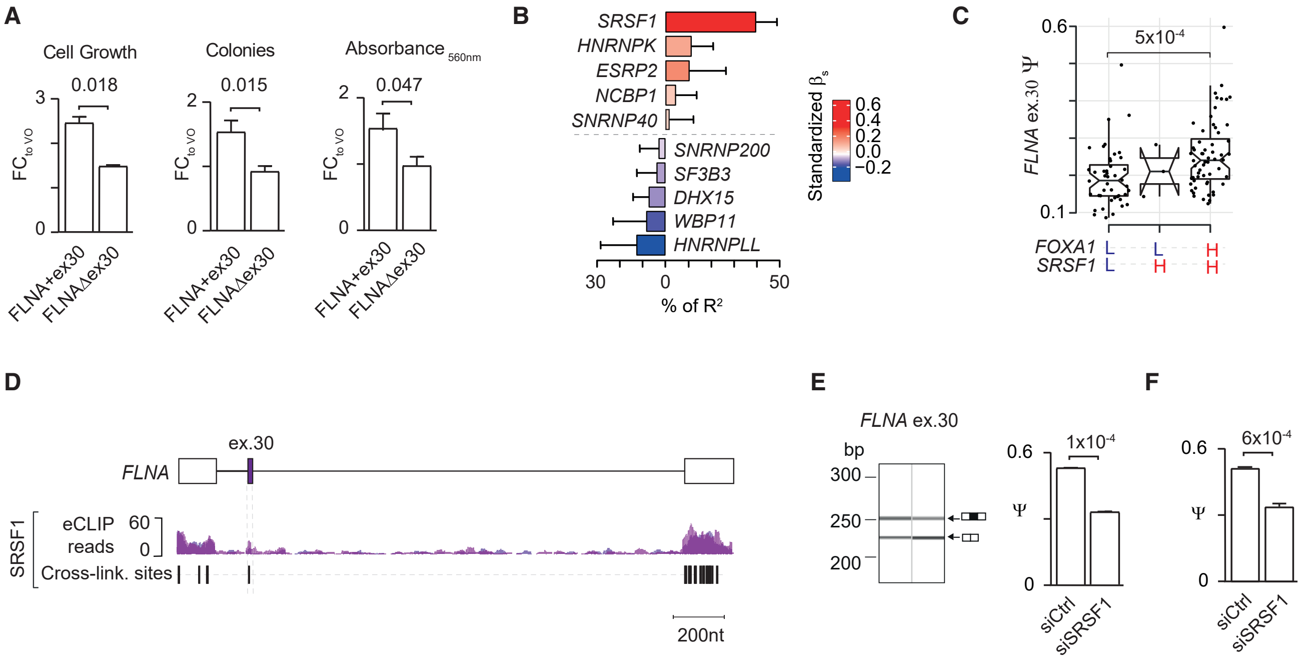

Figure 6. *FLNA* exon 30 inclusion promotes PC cell growth and is controlled by SRSF1

(A) Bar plot shows mean fold change in PC3 cell growth (left panel) measured by MTT assay following transfection with 100 ng of plasmid DNA vector encoding *FLNA* with or without exon 30 (i.e., FLNA+ex30 or FLNAΔex30, respectively, or VO control, biological triplicates). Bar plot shows mean fold change in PC3 clonogenic potential (middle and right panels) measured by crystal violet assays following transfection with 2 μg of plasmid DNA vector encoding *FLNA* with or without exon 30 (i.e., FLNA+ex30 or FLNAΔex30, respectively, or VO control). Both colony number (middle panel) and staining intensity (right panel) are shown (five biological replicates). Two-tailed t test was used to compare conditions.

(B) Results of multivariable covariance analysis between *FLNA* exon 30 inclusion levels and SRG expression levels. Color key indicates the standardized β coefficients of the model.

(C) Distribution of *FLNA* exon 30 inclusion levels in primary PC patients stratified by high or low expression ($\geq 75^{th}$ and $\leq 25^{th}$ percentile, respectively) of *FOXA1* and *SRSF1*. Two-tailed Wilcoxon rank-sum test was used to compare conditions. Only significant results are reported.

(D) SRSF1 eCLIP density read distribution in HepG2 cells in the alternatively spliced region of *FLNA* exon 30. Significant crosslinked sites detected by iCounts for SRSF1 are shown in black.

(E and F) Bar plots show Ψs of *FLNA* exon 30 in PC3 cells upon depletion of *SRSF1* with one siRNA duplex (40 nM for 72 h) in PC3 cells quantified by (E) endpoint PCR splicing assays using the QIAxcel capillary electrophoresis device and (F) by ddPCR. Representative capillary gel electrophoretogram (QIAxcel) shows two bands representing *FLNA* transcripts including or excluding exon 30 which were quantified to determine Ψ (E) (left panel). Two-tailed t test was used to compare biological triplicates of the different conditions.

to a greater extent than the non-pioneer TFs, of which AR and MYC are documented to impact splicing regulation in PC (Phillips et al., 2020; Shah et al., 2020). Therefore, FOXA1 may open multiple channels to transmit transcriptional signals to SRG loci as exemplified by a common pioneer function for AR- and MYC-driven PC transcriptional programs (Barfeld et al., 2017).

By assessing AS changes in primary PC and cell lines, we demonstrate that FOXA1 calibrates the landscape of exon utilization toward an equilibrium that solidifies the production of dominant isoforms. This phenomenon is largely achieved by silencing lowly included exons in a consistent manner across tumors, but crucially also by enhancing highly included ones. Therefore, FOXA1 ultimately limits protein diversity toward isoforms that are functional for cells. We show that exons responding to FOXA1 are alternatively spliced by an "exon definition" mechanism, being shorter with longer flanking introns, strongly conserved across species, and, for a small fraction, marked by chromatin modifications (Agirre et al., 2021; Keren et al., 2010). A smaller exon size and higher intronic sequence conservation have been associated with a greater exon silencing, under evolutionary constraints, to control relative isoform frequencies (Baek and Green, 2005). By integrating analyses of *cis*-acting elements and *trans*-acting factors, we demonstrate that FOXA1 calibrates AS by enlisting splicing factors under its transcriptional control, including binding of PTBP1, U2AF2, and HNRNPC at 3′ ss (König et al., 2010; Sutandy et al., 2018; Xue et al., 2009), and HNRNPK at upstream intron-exon boundary and within downstream introns, respectively (Van Nostrand et al., 2020a, 2020b). It is fascinating that FOXA1 increases the inclusion of exons that are already highly included while reducing lowly included exons. This latter group indicates that FOXA1 is a genuine regulator of AS and not just an enhancer of splicing efficiency per se.

It is likely significant that FOXA1-mediated AS preferentially impacts on SRGs themselves, suggesting that FOXA1 may be involved in a known regulatory feedback loop exploited by splicing factors to modulate their own protein expression levels (Lareau et al., 2007). Interestingly, our results indicate that high *FOXA1* expression in PC mostly inhibits the inclusion of

CellPress
OPEN ACCESS

NMD-determinant PTC-introducing ''poison'' exons. We hypothesize, therefore, that FOXA1-mediated AS restricts proteome diversity by influencing isoform degradation, particularly in SRGs. Recently, MYC has been implicated as a regulator of AS-coupled NMD in PC (Nasif et al., 2018; Pervouchine et al., 2019; Phillips et al., 2020). It is tempting to speculate that FOXA1, as a pliant regulator, may pioneer MYC to control transcription of specific SRGs and fine-tune AS in PC. Further functional studies are necessary to determine whether FOXA1 cooperates with specific TFs, chromatin modifiers, and RNA polymerase II, to rewire the AS landscape of PC.

Clearly the systems-wide impact on AS mediated by FOXA1 is likely to have a profound effect on cancer severity. From a clinical perspective, we found that FOXA1 enhanced the inclusion of two NMD-determinant exons that are strong biomarkers of disease recurrence. Of these, we established a role for the FOXA1-enhanced PTC-preventing exon 30 in the cancer gene *FLNA* as a promoter of PC cell growth. We demonstrate that the inclusion of *FLNA* exon 30 is controlled primarily by SRSF1, which was the first proto-oncogenic splicing factor enacting some of the oncogenic functions of MYC (Das et al., 2012).

In summary, we reveal a novel role for the pioneer TF FOXA1 in orchestrating AS regulation in PC at different stages of gene expression. By transcriptionally regulating *trans*-acting factors, FOXA1 exploits an exon definition model to control relative isoform expression thereby fine-tuning proteome diversity. This splicing equilibrium favors the production of dominant isoforms, especially including those that escape NMD. FOXA1-mediated splicing regulation affects clinically relevant coding regions of the genome underlying PC patient survival.

Limitations of the study

Our characterization of AS regulation in PC is limited to the contribution of four key oncogenic TFs with recurrent activating alterations across PC patients. In light of a long tail of oncogenic drivers underpinning a heterogeneous disease, we cannot exclude the influence of other transcriptional regulators. The analysis of FOXA1-mediated AS regulation was limited to primary PCs as splicing data for mCRPCs were not available. Although we recapitulated our results on metastatic PC cells, the generalizability of our findings to other clinical PC disease states remains to be elucidated.

Our work is based on novel computational analyses that provide unique insights into AS regulation by FOXA1, including the involvement of candidate SRGs and, to a minor extent, chromatin regulators. However, the mechanistic details as to how FOXA1 modulates SRG expression, cooperates with epi-transcriptional regulators, and affects AS decisions remain questions to address in future studies. Although we highlighted candidate prognostic AS events that could be exploited as biomarkers and therapeutic targets, further studies are required to determine their value in the context of FOXA1. Furthermore, a lack of preclinical phenotyping in our study limits the immediate clinical translation of our findings.

A potential confounder in the analysis of PC transcriptomes from bulk sequencing experiments is the contamination in low purity samples arising from benign prostatic epithelial, stromal, or immune cells. However, we performed computational validations showing that FOXA1 orchestrates AS regulation regardless of purity constraints (Figure S6; STAR Methods).

STAR★METHODS

Detailed methods are provided in the online version of this paper and include the following:

- KEY RESOURCES TABLE
- RESOURCE AVAILABILITY
  - Lead contact
  - Materials availability
  - Data and code availability
- EXPERIMENTAL MODEL AND SUBJECT DETAILS
  - Cell lines
- METHOD DETAILS
  - RNA-seq patient datasets
  - Selection of splicing-related genes
  - Multivariable covariance analysis
  - Architectural features of TF transcriptional control in prostate cancer
  - Over-Representation Analysis
  - Differentially expressed splicing-related genes
  - Cell transfections
  - Antibodies, plasmids, and oligonucleotides
  - SDS-PAGE and Western blotting
  - Generation of RNA-seq libraries
  - Gene expression analyses of RNA-seq data
  - Quantitative reverse transcription PCR
  - Alternative splicing analysis of primary PC
  - Alternative splicing analysis of cell lines
  - Nonsense-mediated decay determinant exons
  - Splicing-associated chromatin signatures
  - Splicing code analysis
  - Survival analysis
  - Splicing assays
  - Cell viability and colony formation assays
  - Assessment of tumor purity constraints
- QUANTIFICATION AND STATISTICAL ANALYSIS

SUPPLEMENTAL INFORMATION

Supplemental information can be found online at https://doi.org/10.1016/j.celrep.2022.111404.

ACKNOWLEDGMENTS

The following reagents were gifted: VCaP cells (Yong-Jie Lu, Barts Cancer Institute, UK), pcDNA3-FOXA1 (Jason Carroll, Cancer Research UK Cambridge Institute, UK), pcDNA3-myc-Flna WT (John Blenis, Beth Israel Deaconess Medical Center, USA). We thank Jernej Ule (Francis Crick Institute, UK) for providing iCounts processed data of ENCODE eCLIP experiments. This research has received funding from AIRC under MFAG 2017 ID 20566 (to M.C.) and IG 2017 ID 20240 (to S.O.), FPRC 5xmille 2018 Ministero Salute, project "ADVANCE/A-Bi-C": Italian Ministry of Health, Ricerca Corrente 2021 (to M.C.), the Compagnia di San Paolo (to M.C.), joint Royal College of Surgeons of England/Cancer Research UK Clinician Scientist Fellowship in Surgery (C19198/A15339 to P.R.), The Urology Foundation and John Black Charitable Foundation (to P.R.), The Royal College of Surgeons of England (PO117626 to P.R.) Barts Charity (MGU0533 to P.R.), and Orchid Charity (to P.R.). We sincerely thank Giuseppe Basso (1948–2021) for his guidance,

teaching, and dedication to foster research programs aimed at the needs of patients.

AUTHOR CONTRIBUTIONS

Conceptualization, M.D.G., J.G.F., S.P., P.R., and M.C.; methodology, M.D.G., J.G.F., S.P., A.R., and M.C.; software, M.D.G., S.P., A.R., and M.C.; validation, M.D.G., J.G.F., and S.P.; formal analysis, M.D.G., S.P., and A.R.; investigation, M.D.G., J.G.F., S.P., A.R., L.C., F.G., C.P., F.A., R.A., and S.G.; resources, S.O., G.B., P.R., and M.C.; data curation, M.D.G., J.G.F., and S.P.; writing – original draft, M.D.G., J.G.F., S.P., A.R., P.R., and M.C.; writing – review & editing, M.D.G., J.G.F., P.R., and M.C.; visualization, M.D.G., J.G.F., P.R., and M.C.; supervision, P.R. and M.C.; project administration, P.R. and M.C.; funding acquisition, P.R. and M.C.

DECLARATION OF INTERESTS

The authors declare no competing interests.

Received: November 30, 2021
Revised: May 28, 2022
Accepted: September 1, 2022
Published: September 27, 2022

REFERENCES

Agirre, E., Oldfield, A.J., Bellora, N., Segelle, A., and Luco, R.F. (2021). Splicing-associated chromatin signatures: a combinatorial and position-dependent role for histone marks in splicing definition. Nat. Commun. *12*, 682.

Aran, D., Sirota, M., and Butte, A.J. (2015). Systematic pan-cancer analysis of tumour purity. Nat. Commun. *6*, 8971.

Aran, D., Hu, Z., and Butte, A.J. (2017). xCell: digitally portraying the tissue cellular heterogeneity landscape. Genome Biol. *18*, 220.

Baca, S.C., Takeda, D.Y., Seo, J.-H., Hwang, J., Ku, S.Y., Arafeh, R., Arnoff, T., Agarwal, S., Bell, C., O'Connor, E., et al. (2021). Reprogramming of the FOXA1 cistrome in treatment-emergent neuroendocrine prostate cancer. Nat. Commun. *12*, 1979.

Baek, D., and Green, P. (2005). Sequence conservation, relative isoform frequencies, and nonsense-mediated decay in evolutionarily conserved alternative splicing. Proc. Natl. Acad. Sci. USA *102*, 12813–12818.

Bahmad, H.F., Jalloul, M., Azar, J., Moubarak, M.M., Samad, T.A., Mukherji, D., Al-Sayegh, M., and Abou-Kheir, W. (2021). Tumor microenvironment in prostate cancer: toward identification of novel molecular biomarkers for diagnosis, prognosis, and therapy development. Front. Genet. *12*, 652747.

Barfeld, S.J., Urbanucci, A., Itkonen, H.M., Fazli, L., Hicks, J.L., Thiede, B., Rennie, P.S., Yegnasubramanian, S., DeMarzo, A.M., and Mills, I.G. (2017). c-Myc antagonises the transcriptional activity of the androgen receptor in prostate cancer affecting key gene networks. EBioMedicine *18*, 83–93.

Beltran, H., Prandi, D., Mosquera, J.M., Benelli, M., Puca, L., Cyrta, J., Marotz, C., Giannopoulou, E., Chakravarthi, B.V.S.K., Varambally, S., et al. (2016). Divergent clonal evolution of castration-resistant neuroendocrine prostate cancer. Nat. Med. *22*, 298–305.

Bonnal, S.C., López-Oreja, I., and Valcárcel, J. (2020). Roles and mechanisms of alternative splicing in cancer — implications for care. Nat. Rev. Clin. Oncol. *17*, 457–474.

Cerami, E., Gao, J., Dogrusoz, U., Gross, B.E., Sumer, S.O., Aksoy, B.A., Jacobsen, A., Byrne, C.J., Heuer, M.L., Larsson, E., et al. (2012). The cBio cancer genomics portal: an open platform for exploring multidimensional cancer genomics data. Cancer Discov. *2*, 401–404.

Cereda, M., Pozzoli, U., Rot, G., Juvan, P., Schweitzer, A., Clark, T., and Ule, J. (2014). RNAmotifs: prediction of multivalent RNA motifs that control alternative splicing. Genome Biol. *15*, R20.

Cereda, M., Gambardella, G., Benedetti, L., Iannelli, F., Patel, D., Basso, G., Guerra, R.F., Mourikis, T.P., Puccio, I., Sinha, S., et al. (2016). Patients with genetically heterogeneous synchronous colorectal cancer carry rare damaging germline mutations in immune-related genes. Nat. Commun. *7*, 12072.

Chen, Y., Chi, P., Rockowitz, S., Iaquinta, P.J., Shamu, T., Shukla, S., Gao, D., Sirota, I., Carver, B.S., Wongvipat, J., et al. (2013). ETS factors reprogram the androgen receptor cistrome and prime prostate tumorigenesis in response to PTEN loss. Nat. Med. *19*, 1023–1029.

Corces, M.R., Granja, J.M., Shams, S., Louie, B.H., Seoane, J.A., Zhou, W., Silva, T.C., Groeneveld, C., Wong, C.K., Cho, S.W., et al. (2018). The chromatin accessibility landscape of primary human cancers. Science *362*, eaav1898.

Das, S., Anczuków, O., Akerman, M., and Krainer, A.R. (2012). Oncogenic splicing factor SRSF1 is a critical transcriptional target of MYC. Cell Rep. *1*, 110–117.

de la Grange, P., Gratadou, L., Delord, M., Dutertre, M., and Auboeuf, D. (2010). Splicing factor and exon profiling across human tissues. Nucleic Acids Res. *38*, 2825–2838.

Denichenko, P., Mogilevsky, M., Cléry, A., Welte, T., Biran, J., Shimshon, O., Barnabas, G.D., Danan-Gotthold, M., Kumar, S., Yavin, E., et al. (2019). Specific inhibition of splicing factor activity by decoy RNA oligonucleotides. Nat. Commun. *10*, 1590.

Dobin, A., Davis, C.A., Schlesinger, F., Drenkow, J., Zaleski, C., Jha, S., Batut, P., Chaisson, M., and Gingeras, T.R. (2013). STAR: ultrafast universal RNA-seq aligner. Bioinformatics *29*, 15–21.

Fei, T., Li, W., Peng, J., Xiao, T., Chen, C.-H., Wu, A., Huang, J., Zang, C., Liu, X.S., and Brown, M. (2019). Deciphering essential cistromes using genome-wide CRISPR screens. Proc. Natl. Acad. Sci. USA *116*, 25186–25195.

Feng, H., Bao, S., Rahman, M.A., Weyn-Vanhentenryck, S.M., Khan, A., Wong, J., Shah, A., Flynn, E.D., Krainer, A.R., and Zhang, C. (2019). Modeling RNA-binding protein specificity in vivo by precisely registering protein-RNA cross-link sites. Mol. Cell *74*, 1189–1204.e6.

Frankish, A., Diekhans, M., Ferreira, A.M., Johnson, R., Jungreis, I., Loveland, J., Mudge, J.M., Sisu, C., Wright, J., Armstrong, J., et al. (2019). GENCODE reference annotation for the human and mouse genomes. Nucleic Acids Res. *47*, D766–D773.

Frankiw, L., Baltimore, D., and Li, G. (2019). Alternative mRNA splicing in cancer immunotherapy. Nat. Rev. Immunol. *19*, 675–687.

Grömping, U. (2006). Relative importance for linear regression in R: the package relaimpo. J. Stat. Softw. *17*. https://doi.org/10.18637/jss.v017.i01.

Hawksworth, D., Ravindranath, L., Chen, Y., Furusato, B., Sesterhenn, I.A., McLeod, D.G., Srivastava, S., and Petrovics, G. (2010). Overexpression of C-MYC oncogene in prostate cancer predicts biochemical recurrence. Prostate Cancer Prostatic Dis. *13*, 311–315.

Kahles, A., Lehmann, K.-V., Toussaint, N.C., Hüser, M., Stark, S.G., Sachsenberg, T., Stegle, O., Kohlbacher, O., Sander, C., Cancer Genome Atlas Research Network, et al. (2018). Comprehensive analysis of alternative splicing across tumors from 8, 705 patients. Cancer Cell *34*, 211–224.e6.

Kawamura, N., Nimura, K., Saga, K., Ishibashi, A., Kitamura, K., Nagano, H., Yoshikawa, Y., Ishida, K., Nonomura, N., Arisawa, M., et al. (2019). SF3B2-Mediated RNA splicing drives human prostate cancer progression. Cancer Res. *79*, 5204–5217.

Kent, W.J., Sugnet, C.W., Furey, T.S., Roskin, K.M., Pringle, T.H., Zahler, A.M., and Haussler, D. (2002). The human genome browser at UCSC. Genome Res. *12*, 996–1006.

Keren, H., Lev-Maor, G., and Ast, G. (2010). Alternative splicing and evolution: diversification, exon definition and function. Nat. Rev. Genet. *11*, 345–355.

König, J., Zarnack, K., Rot, G., Curk, T., Kayikci, M., Zupan, B., Turner, D.J., Luscombe, N.M., and Ule, J. (2010). iCLIP reveals the function of hnRNP particles in splicing at individual nucleotide resolution. Nat. Struct. Mol. Biol. *17*, 909–915.

Kron, K.J., Murison, A., Zhou, S., Huang, V., Yamaguchi, T.N., Shiah, Y.J., Fraser, M., van der Kwast, T., Boutros, P.C., Bristow, R.G., et al. (2017). TMPRSS2-ERG fusion co-opts master transcription factors and activates NOTCH signaling in primary prostate cancer. Nat. Genet. *49*, 1336–1345.

Kurosaki, T., Popp, M.W., and Maquat, L.E. (2019). Quality and quantity control of gene expression by nonsense-mediated mRNA decay. Nat. Rev. Mol. Cell Biol. *20*, 384.

Lareau, L.F., Inada, M., Green, R.E., Wengrod, J.C., and Brenner, S.E. (2007). Unproductive splicing of SR genes associated with highly conserved and ultraconserved DNA elements. Nature *446*, 926–929.

Lauria, A., Peirone, S., Giudice, M.D., Priante, F., Rajan, P., Caselle, M., Oliviero, S., and Cereda, M. (2020). Identification of altered biological processes in heterogeneous RNA-sequencing data by discretization of expression profiles. Nucleic Acids Res. *48*, 1730–1747.

Lausen, B., and Schumacher, M. (1992). Maximally selected rank statistics. Biometrics *48*, 73–85.

Lawrence, M., Huber, W., Pagès, H., Aboyoun, P., Carlson, M., Gentleman, R., Morgan, M.T., and Carey, V.J. (2013). Software for computing and annotating genomic ranges. PLoS Comput. Biol. *9*, e1003118.

Lee, S.C.-W., and Abdel-Wahab, O. (2016). Therapeutic targeting of splicing in cancer. Nat. Med. *22*, 976–986.

Li, J., and Tibshirani, R. (2013). Finding consistent patterns: a nonparametric approach for identifying differential expression in RNA-seq data. Stat. Methods Med. Res. *22*, 519–536.

Li, Y., Sahni, N., Pancsa, R., McGrail, D.J., Xu, J., Hua, X., Coulombe-Huntington, J., Ryan, M., Tychhon, B., Sudhakar, D., et al. (2017). Revealing the determinants of Widespread alternative splicing perturbation in cancer. Cell Rep. *21*, 798–812.

Liao, Y., Smyth, G.K., and Shi, W. (2014). featureCounts: an efficient general purpose program for assigning sequence reads to genomic features. Bioinformatics *30*, 923–930.

Lindeman, R.H. (1980). Introduction to Bivariate and Multivariate Analysis.

Love, M.I., Huber, W., and Anders, S. (2014). Moderated estimation of fold change and dispersion for RNA-seq data with DESeq2. Genome Biol. *15*, 550.

Lupien, M., Eeckhoute, J., Meyer, C.A., Wang, Q., Zhang, Y., Li, W., Carroll, J.S., Liu, X.S., and Brown, M. (2008). FoxA1 translates epigenetic signatures into enhancer-driven lineage-specific transcription. Cell *132*, 958–970.

Mazin, P.V., Khaitovich, P., Cardoso-Moreira, M., and Kaessmann, H. (2021). Alternative splicing during mammalian organ development. Nat. Genet. *53*, 925–934.

Nasif, S., Contu, L., and Mühlemann, O. (2018). Beyond quality control: the role of nonsense-mediated mRNA decay (NMD) in regulating gene expression. Semin. Cell Dev. Biol. *75*, 78–87.

Network Cancer (2015). The molecular taxonomy of primary prostate cancer. Cell *163*, 1011–1025.

Oki, S., Ohta, T., Shioi, G., Hatanaka, H., Ogasawara, O., Okuda, Y., Kawaji, H., Nakaki, R., Sese, J., and Meno, C. (2018). ChIP-atlas: a data-mining suite powered by full integration of public ChIP-seq data. EMBO Rep. *19*, e46255.

Parolia, A., Cieslik, M., Chu, S.-C., Xiao, L., Ouchi, T., Zhang, Y., Wang, X., Vats, P., Cao, X., Pitchiaya, S., et al. (2019). Distinct structural classes of activating FOXA1 alterations in advanced prostate cancer. Nature *571*, 413–418.

Paschalis, A., Sharp, A., Welti, J.C., Neeb, A., Raj, G.V., Luo, J., Plymate, S.R., and de Bono, J.S. (2018). Alternative splicing in prostate cancer. Nat. Rev. Clin. Oncol. *15*, 663–675.

Pereira, B., Billaud, M., and Almeida, R. (2017). RNA-binding proteins in cancer: old players and new actors. Trends Cancer *3*, 506–528.

Pervouchine, D., Popov, Y., Berry, A., Borsari, B., Frankish, A., and Guigó, R. (2019). Integrative transcriptomic analysis suggests new autoregulatory splicing events coupled with nonsense-mediated mRNA decay. Nucleic Acids Res. *47*, 5293–5306.

Phillips, J.W., Pan, Y., Tsai, B.L., Xie, Z., Demirdjian, L., Xiao, W., Yang, H.T., Zhang, Y., Lin, C.H., Cheng, D., et al. (2020). Pathway-guided analysis identifies Myc-dependent alternative pre-mRNA splicing in aggressive prostate cancers. Proc. Natl. Acad. Sci. USA *117*, 5269–5279.

Pomerantz, M.M., Li, F., Takeda, D.Y., Lenci, R., Chonkar, A., Chabot, M., Cejas, P., Vazquez, F., Cook, J., Shivdasani, R.A., et al. (2015). The androgen receptor cistrome is extensively reprogrammed in human prostate tumorigenesis. Nat. Genet. *47*, 1346–1351.

Qiu, X., Boufaied, N., Hallal, T., Feit, A., de Polo, A., Luoma, A.M., Larocque, J., Zadra, G., Xie, Y., Gu, S., et al. (2021). MYC drives aggressive prostate cancer by disrupting transcriptional pause release at androgen receptor targets. Nat. Commun. *13*, 2559.

Quinlan, A.R., and Hall, I.M. (2010). BEDTools: a flexible suite of utilities for comparing genomic features. Bioinformatics *26*, 841–842.

Rajan, P., Elliott, D.J., Robson, C.N., and Leung, H.Y. (2009). Alternative splicing and biological heterogeneity in prostate cancer. Nat. Rev. Urol. *6*, 454–460.

Ramanand, S.G., Chen, Y., Yuan, J., Daescu, K., Lambros, M.B., Houlahan, K.E., Carreira, S., Yuan, W., Baek, G., Sharp, A., et al. (2020). The landscape of RNA polymerase II–associated chromatin interactions in prostate cancer. J. Clin. Invest. *130*, 3987–4005.

Ray, D., Kazan, H., Cook, K.B., Weirauch, M.T., Najafabadi, H.S., Li, X., Gueroussov, S., Albu, M., Zheng, H., Yang, A., et al. (2013). A compendium of RNA-binding motifs for decoding gene regulation. Nature *499*, 172–177.

Rebello, R.J., Oing, C., Knudsen, K.E., Loeb, S., Johnson, D.C., Reiter, R.E., Gillessen, S., Van der Kwast, T., and Bristow, R.G. (2021). Prostate cancer. Nat. Rev. Dis. Primers *7*, 9.

Rizzo, F., Nizzardo, M., Vashisht, S., Molteni, E., Melzi, V., Taiana, M., Salani, S., Santonicola, P., Di Schiavi, E., Bucchia, M., et al. (2019). Key role of SMN/SYNCRIP and RNA-Motif 7 in spinal muscular atrophy: RNA-Seq and motif analysis of human motor neurons. Brain *142*, 276–294.

Robinson, M.D., McCarthy, D.J., and Smyth, G.K. (2010). edgeR: a Bioconductor package for differential expression analysis of digital gene expression data. Bioinformatics *26*, 139–140.

Robinson, D., Van Allen, E.M., Wu, Y.M., Schultz, N., Lonigro, R.J., Mosquera, J.M., Montgomery, B., Taplin, M.E., Pritchard, C.C., Attard, G., et al. (2015). Integrative clinical genomics of advanced prostate cancer. Cell *162*, 454–1228.

Saraiva-Agostinho, N., and Barbosa-Morais, N.L. (2019). psichomics: graphical application for alternative splicing quantification and analysis. Nucleic Acids Res. *47*, e7.

Saulnier, O., Guedri-Idjouadiene, K., Aynaud, M.-M., Chakraborty, A., Bruyr, J., Pineau, J., O'Grady, T., Mirabeau, O., Grossetête, S., Galvan, B., et al. (2021). ERG transcription factors have a splicing regulatory function involving RBFOX2 that is altered in the EWS-FLI1 oncogenic fusion. Nucleic Acids Res. *49*, 5038–5056.

Sebestyén, E., Singh, B., Miñana, B., Pagès, A., Mateo, F., Pujana, M.A., Valcárcel, J., and Eyras, E. (2016). Large-scale analysis of genome and transcriptome alterations in multiple tumors unveils novel cancer-relevant splicing networks. Genome Res. *26*, 732–744.

Seiler, M., Peng, S., Agrawal, A.A., Palacino, J., Teng, T., Zhu, P., Smith, P.G., Cancer Genome Atlas Research Network; Buonamici, S., and Yu, L. (2018). Somatic mutational landscape of splicing factor genes and their functional consequences across 33 cancer types. Cell Rep. *23*, 282–296.e4.

Shah, K., Gagliano, T., Garland, L., O'Hanlon, T., Bortolotti, D., Gentili, V., Rizzo, R., Giamas, G., and Dean, M. (2020). Androgen receptor signaling regulates the transcriptome of prostate cancer cells by modulating global alternative splicing. Oncogene *39*, 6172–6189.

Shi, Y., Chinnaiyan, A.M., and Jiang, H. (2015). rSeqNP: a non-parametric approach for detecting differential expression and splicing from RNA-Seq data. Bioinformatics *31*, 2222–2224.

Sterne-Weiler, T., Weatheritt, R.J., Best, A.J., Ha, K.C.H., and Blencowe, B.J. (2018). Efficient and accurate quantitative profiling of alternative splicing patterns of any complexity on a laptop. Mol. Cell *72*, 187–200.e6.

Subramanian, A., Tamayo, P., Mootha, V.K., Mukherjee, S., Ebert, B.L., Gillette, M.A., Paulovich, A., Pomeroy, S.L., Golub, T.R., Lander, E.S., et al. (2005). Gene set enrichment analysis: a knowledge-based approach for interpreting genome-wide expression profiles. Proc. Natl. Acad. Sci. USA *102*, 15545–15550.

Sutandy, F.X.R., Ebersberger, S., Huang, L., Busch, A., Bach, M., Kang, H.-S., Fallmann, J., Maticzka, D., Backofen, R., Stadler, P.F., et al. (2018). In vitro iCLIP-based modeling uncovers how the splicing factor U2AF2 relies on regulation by cofactors. Genome Res. *28*, 699–713.

Therneau, T.M., and Grambsch, P.M. (2000). Modeling Survival Data: Extending the Cox Model. Statistics for Biology and Health (Springer). https://doi.org/10.1007/978-1-4757-3294-8_3.

Thomas, J.D., Polaski, J.T., Feng, Q., De Neef, E.J., Hoppe, E.R., McSharry, M.V., Pangallo, J., Gabel, A.M., Belleville, A.E., Watson, J., et al. (2020). RNA isoform screens uncover the essentiality and tumor-suppressor activity of ultraconserved poison exons. Nat. Genet. *52*, 84–94.

Thompson, J.R., Marcelino, L.A., and Polz, M.F. (2002). Heteroduplexes in mixed-template amplifications: formation, consequence and elimination by “reconditioning PCR. Nucleic Acids Res. *30*, 2083–2088.

Van Nostrand, E.L., Pratt, G.A., Yee, B.A., Wheeler, E.C., Blue, S.M., Mueller, J., Park, S.S., Garcia, K.E., Gelboin-Burkhart, C., Nguyen, T.B., et al. (2020a). Principles of RNA processing from analysis of enhanced CLIP maps for 150 RNA binding proteins. Genome Biol. *21*, 90.

Van Nostrand, E.L., Freese, P., Pratt, G.A., Wang, X., Wei, X., Xiao, R., Blue, S.M., Chen, J.-Y., Cody, N.A.L., Dominguez, D., et al. (2020b). A large-scale binding and functional map of human RNA-binding proteins. Nature *583*, 711–719.

Venables, J.P., Klinck, R., Koh, C., Gervais-Bird, J., Bramard, A., Inkel, L., Durand, M., Couture, S., Froehlich, U., Lapointe, E., et al. (2009). Cancer-associated regulation of alternative splicing. Nat. Struct. Mol. Biol. *16*, 670–676.

Vorontsov, I.E., Kulakovskiy, I.V., and Makeev, V.J. (2013). Jaccard index based similarity measure to compare transcription factor binding site models. Algorithms Mol. Biol. *8*, 23.

Wang, Z.-L., Li, B., Luo, Y.-X., Lin, Q., Liu, S.-R., Zhang, X.-Q., Zhou, H., Yang, J.-H., and Qu, L.-H. (2018). Comprehensive genomic characterization of RNA-binding proteins across human cancers. Cell Rep. *22*, 286–298.

Woo, M.S., Ohta, Y., Rabinovitz, I., Stossel, T.P., and Blenis, J. (2004). Ribosomal S6 kinase (RSK) regulates phosphorylation of filamin A on an important regulatory site. Mol. Cell Biol. *24*, 3025–3035.

Xue, Y., Zhou, Y., Wu, T., Zhu, T., Ji, X., Kwon, Y.-S., Zhang, C., Yeo, G., Black, D.L., Sun, H., et al. (2009). Genome-wide analysis of PTB-RNA interactions reveals a strategy used by the general splicing repressor to modulate exon inclusion or skipping. Mol. Cell *36*, 996–1006.

Yu, G., Wang, L.-G., Han, Y., and He, Q.-Y. (2012). clusterProfiler: an R package for comparing biological themes among gene clusters. OMICS *16*, 284–287.

Zhang, D., Hu, Q., Liu, X., Ji, Y., Chao, H.-P., Liu, Y., Tracz, A., Kirk, J., Buonamici, S., Zhu, P., et al. (2020). Intron retention is a hallmark and spliceosome represents a therapeutic vulnerability in aggressive prostate cancer. Nat. Commun. *11*, 2089.

Zheng, L., Qian, B., Tian, D., Tang, T., Wan, S., Wang, L., Zhu, L., and Geng, X. (2015). FOXA1 positively regulates gene expression by changing gene methylation status in human breast cancer MCF-7 cells. Int. J. Clin. Exp. Pathol. *8*, 96–106.

Zhou, Y., Han, C., Wang, E., Lorch, A.H., Serafin, V., Cho, B.-K., Guttierrez Diaz, B.T., Calvo, J., Fang, C., Khodadadi-Jamayran, A., et al. (2020). Post-translational regulation of the exon skipping machinery controls aberrant splicing in leukemia. Cancer Discov. *10*, 1388–1409.

Zhu, A., Srivastava, A., Ibrahim, J.G., Patro, R., and Love, M.I. (2019). Nonparametric expression analysis using inferential replicate counts. Nucleic Acids Res. *47*, e105.

STAR★METHODS

KEY RESOURCES TABLE

REAGENT or RESOURCE	SOURCE	IDENTIFIER
**Antibodies**		
Rabbit monoclonal [EPR10881] anti-FOXA1	Abcam	Abcam Cat# ab23738; RRID:AB_2104842
Mouse monoclonal anti-Beta-Actin	Sigma	Sigma-Aldrich Cat# A1978; RRID:AB_476692
Mouse monoclonal [G122-434] anti-AR	BD Biosciences	BD Biosciences Cat# 554225; RRID:AB_395316
Mouse monoclonal [96] anti-SRSF1	Thermo Fisher Scientific	Thermo Fisher Scientific Cat# 32-4500; RRID:AB_2533079
Goat Anti-Mouse Immunoglobulins/HRP antibody	Agilent Technologies	Agilent Cat# P0447; RRID:AB_2617137
Goat Anti-Rabbit Immunoglobulins/HRP antibody	Agilent Technologies	Agilent Cat# P0448; RRID:AB_2617138
**Chemicals, peptides, and recombinant proteins**		
ViaFect	Promega	Cat# E4981
RNAiMax	Thermo Fisher Scientific	Cat# 13778-075
PVDF (polyvinylidene difluoride) membrane	Sigma	Cat# 000000003010040001
Bovine Serum Albumin (BSA)	Sigma	Cat# A9418
Luminata Crescendo Western HRP substrate	Thermo Fisher Scientific	Cat# 10776189
TRI Reagent	Invitrogen	Cat# AM9738
SYBR green master mix	NEB	Cat# M3003
Taq Polymerase	NEB	Cat# M0273
Deoxynucleotide (dNTP) Solution Mix	NEB	Cat# N0447
(3-(4,5-Dimethylthiazol-2-yl)-2, 5-Diphenyltetrazolium Bromide) (MTT)	Alfa Aesar	Cat# L11939.06
Dimethyl Sulfoxide (DMSO)	Thermo Fisher Scientific	Cat# 10213810
**Critical commercial assays**		
TruSeq total RNA	Illumina	Cat# 20020596
TruSeq stranded mRNA	Illumina	Cat# 20020594
Q5 Site-Directed Mutagenesis Kit	NEB	Cat# E0554S
Bicinchoninic acid (BCA) assay	Thermo Fisher Scientific	Cat# 10678484
RNA Clean and Concentrator	Zymo Research	Cat# R1013
Qubit RNA HS Assay Kit	Thermo Fisher Scientific	Cat# Q32852
RNA 6000 Nano kit	Agilent Technologies	Cat# 5067-1511
cDNA reverse transcription kit	Applied Biosystems	Cat# 4368814
QIAxcel DNA High Resolution Kit (1200)	QIAgen	Cat# 929002
ddPCR^{TM} Supermix for Probes (No dUTP)	Bio-Rad	Cat# #1863024
**Deposited data**		
PC3 and VCaP RNA-Seq	This Paper	GEO: GSE193127
Differential splicing results in PC3 and VCaP RNA-seq data	This Paper	Mendeley Data: https://doi.org/10.17632/gtyfsryffj.1
The Cancer Genome Atlas (TCGA) RNA-Seq	(Network Cancer, 2015)	TCGA Data Matrix portal (Level 3, https://tcga-data.nci.nih.gov/tcga/dataAccessMatrix.htm)
Metastatic castration-resistant PC, Stand Up 2 Cancer (SU2C) RNA-Seq	(Robinson et al., 2015)	cBioPortal.org
Neuroendocrine PC	(Beltran et al., 2016)	cBioPortal.org
Publically available ChIP-seq experiments	Gene Expression Omnibus (GEO)	See Table S1 for a list of accession numbers

(Continued on next page)

Continued		
REAGENT or RESOURCE	SOURCE	IDENTIFIER
RNA PolII ChIA-PET data	(Ramanand et al., 2020)	https://www.jci.org/articles/view/134260/sd/2
ATAC-seq data	(Corces et al., 2018)	https://gdc.cancer.gov/about-data/publications/ATACseq-AWG
Splicing data of primary PC	(Kahles et al., 2018)	https://gdc.cancer.gov/about-data/publications/PanCanAtlas-Splicing-2018
Experimental models: Cell lines		
Human: DU145	ATCC	ATCC Cat# HTB-81; RRID:CVCL_0105
Human: PC3	ATCC	ATCC Cat# CRL-7934; RRID:CVCL_0035
Human: LNCaP	ATCC	ATCC Cat# CRL-1740; RRID:CVCL_1379
Human: VCaP	ATCC, Yong-Jie Lu, Barts Cancer Institute, UK	RRID: CVCL_WZ27
Oligonucleotides		
siRNA	See Table S1	See Table S1
Primers	See Table S1	See Table S1
Recombinant DNA		
Plasmid: pcDNA3.1-VO	Professor Jason Carroll, Cancer Research UK Cambridge Institute, UK	N/A
Plasmid: pcDNA3.1-FOXA1	Professor Jason Carroll, Cancer Research UK Cambridge Institute, UK	N/A
Plasmid: pcDNA3-myc-Flna WT (FLNAΔex30)	Addgene: John Blenis, (Woo et al., 2004)	RRID: Addgene_8982
Plasmid: pcDNA3.1-FLNA+ex30	This study	N/A
Software and algorithms		
Image Studio Lite v.5.2	LI-COR	https://www.licor.com/bio/image-studio-lite/ RRID: SCR_013715
Quant Studio Design and Analysis Software v1.5.1	Thermo Fisher Scientific	https://www.thermofisher.com/uk/en/home/global/forms/life-science/quantstudio-3-5-software.html
QIAxcel Screen Gel v1.6.0.10	QIAgen	https://www.qiagen.com/us/products/instruments-and-automation/analytics-software/qiaxcel-screengel-software/
Plate Reader Omega v.5.11.R3	BMG Labtech	https://www.bmglabtech.com/microplate-reader-software/
ImageQuantTL	Amersham	https://www.cytivalifesciences.com/en/us/shop/molecular-biology/nucleic-acid-electrophoresis–blotting–and-detection/molecular-imaging-for-nucleic-acids/imagequant-tl-8-2-image-analysis-software-p-09518
R v.3.5.2	R Project for Statistical Computing	R Project for Statistical Computing, RRID:SCR_001905
RStudio v.1.3.1093	RStudio	RStudio, RRID:SCR_000432
STAR v.2.7.3a	(Dobin et al., 2013)	STAR, RRID:SCR_004463
featureCounts – Subread v.2.0.0	(Liao et al., 2014)	featureCounts, RRID:SCR_012919
R Bioconductor package – DESeq2 v.1.30.1	(Love et al., 2014)	DESeq2, RRID:SCR_015687
R Bioconductor package - edgeR v.3.32.1	(Robinson et al., 2010)	edgeR, RRID:SCR_012802
BEDTools v.2.29.2	(Quinlan and Hall, 2010)	BEDTools, RRID:SCR_006646

(Continued on next page)

Continued

REAGENT or RESOURCE	SOURCE	IDENTIFIER
R package – relaimpo v.2.2-5	(Grömping, 2006)	https://CRAN.R-project.org/package=relaimpo
R Bioconductor package – GenomicFeatures v.1.38.2	(Lawrence et al., 2013)	https://bioconductor.org/packages/GenomicFeatures
R Bioconductor package – GenomicRanges v.1.42.0	(Lawrence et al., 2013)	https://bioconductor.org/packages/GenomicRanges
R Bioconductor package – clusterProfiler v.3.18.1	(Yu et al., 2012)	clusterProfiler, RRID:SCR_016884
Whippet v.0.11	(Sterne-Weiler et al., 2018)	Whippet, RRID:SCR_018349
RNAmotifs	(Cereda et al., 2014)	https://github.com/matteocereda/RNAmotifs
MACRO-APE	(Vorontsov et al., 2013)	https://github.com/autosome-ru/macro-perfectos-ape
R package – survival v.3.2-11	Terry M. Therneau	https://CRAN.R-project.org/package=survival
Scripts and data analysis	This Paper	Mendeley Data: https://doi.org/10.17632/gtyfsryffj.1
Other		
Un-cropped western blot images	This Paper	Mendeley Data: https://doi.org/10.17632/gtyfsryffj.1
QIAxcel report files	This Paper	Mendeley Data: https://doi.org/10.17632/gtyfsryffj.1
Agarose gel images	This Paper	Mendeley Data: https://doi.org/10.17632/gtyfsryffj.1
Un-cropped colony assay wells	This Paper	Mendeley Data: https://doi.org/10.17632/gtyfsryffj.1

RESOURCE AVAILABILITY

Lead contact

Further information and requests for resources and reagents should be directed to and will be fulfilled by the lead contact, Prof Matteo Cereda (matteo.cereda1@unimi.it).

Materials availability

Reagents used in this study are publicly available or available from the lead contact upon request.

Data and code availability

RNA-Seq data have been deposited at Gene Expression Omnibus (GEO) and are publicly available as of the date of publication. Accession numbers are listed in the key resources table. Original Western blot images have been deposited at Mendeley and are publicly available as of the date of publication. The DOI is listed in the key resources table. This paper analyzes existing, publicly available data. These accession numbers for the datasets are listed in the key resources table.

All original code has been deposited at Mendeley and is publicly available as of the date of publication. DOIs are listed in the key resources table.

Any additional information required to reanalyze the data reported in this paper is available from the lead contact upon request.

EXPERIMENTAL MODEL AND SUBJECT DETAILS

Cell lines

DU145 (ATCC Cat# HTB-81; RRID:CVCL_0105), PC3 (ATCC Cat# CRL-7934; RRID:CVCL_0035), LNCaP (ATCC Cat# CRL-1740; RRID:CVCL_1379), and VCaP (ATCC, RRID: CVCL_WZ27) cells were obtained from ATCC and their identities were confirmed by Short Tandem Repeat (STR) profiling (DDC Medical). All cell lines were isolated from Male subjects. Cells were incubated at 37°C, 5% CO_2 in a humidified incubator. Cells were maintained at sub-confluency in RPMI-1640 medium (21875-034, Gibco) or DMEM (41966-029, Gibco) containing 2 mM L-glutamine, supplemented with 10% foetal calf serum (FCS) (Gibco), 100 units/mL penicillin and 100 µg/mL streptomycin (15140-122, Gibco) and regularly tested for the presence of mycoplasma.

METHOD DETAILS

RNA-seq patient datasets

RNA sequencing (RNA-seq) data were obtained from The Cancer Genome Atlas (TCGA) Data Matrix portal (Level 3, https://tcga-data.nci.nih.gov/tcga/dataAccessMatrix.htm) and cBioPortal (Beltran et al., 2016; Cerami et al., 2012; Chen et al., 2013) websites for 409 primary PCs, 118 mCRPCs and 15 NEPCs. The number of transcripts per million reads was measured starting from the scaled estimate expression values provided for 20,531 genes (Cereda et al., 2016). For the metastatic castration-resistant PC dataset, reads per kilobase of transcript per million mapped reads values were converted into transcripts per million. For each transcription factor, the distribution of expression levels across samples was measured. A transcription factor was considered as highly expressed if its transcripts per million value was $\geq 75^{th}$ percentile of its expression distribution across samples (Cereda et al., 2016) (Table S1).

Selection of splicing-related genes

A list of 128 genes in the Kyoto Encyclopedia of Genes and Genomes (KEGG) ‘spliceosome’ pathway was collected from MSigDb version 5 (Subramanian et al., 2005). An additional list of 66 RNA-binding proteins was obtained from the RNAcompete catalogue (Ray et al., 2013) and added to the 128 spliceosome genes. A final set of 148 genes with gene ontology terms related to splicing was retained for further analyses as splicing-related genes.

Multivariable covariance analysis

Relative contributions of expression, or inclusion, levels of multiple factors (*e.g.* genes, exons), namely regressors, to the correlation with a response variable (*e.g.* cumulative expression of splicing factors, FOXA1 expression) were measured using the following approach. Normalized expression, or inclusion levels, of regressors were normalized using a near-zero variance filter, Yeo-Johnson transformation, centering around their mean, and scaling by their standard deviation using the *preProcess* function in the R ‘caret’ package with parameters *method = c("center", "scale", "YeoJohnson", "nzv")*. A generalized linear regression model (GLM) was fitted to the response variable based on the normalized values of regressors using the *glm* function in the R ‘stats’ package. Relative importance of each regressor to the correlation measured by the model was calculated using the function *calc.relimp* in the R ‘relaimpo’ package (Grömping, 2006). This function divides the coefficient of determination R^2 into the contribution of each regressor using the averaging over orderings method (Lindeman, 1980). Confidence intervals were measured using a bootstrap procedure implemented in the function *boot.relimp*. For 1,000 iterations the full observation vectors were resampled and the regressor contributions were calculated.

Architectural features of TF transcriptional control in prostate cancer

A list of 40,495 and 27,580 RNA Pol II–associated enhancer regions, defined by Chromatin Interaction Analysis by Paired-End Tag sequencing (ChIA-PET) in VCaP and LNCaP cell lines, respectively, were obtained from Ramanand et al. (Ramanand et al., 2020). Of these, 31,282 and 17,134 enhancers were associated with at least one putative regulated gene for VCaP and LNCaP cells, respectively. Thus, a total of 115,855 and 41,921 enhancer-gene associations were retained for further analyses. Coordinates of 20,298 protein-coding genes were retrieved from GENCODE GRCh37 version 28 (Frankish et al., 2019). Promoter regions were defined as 2,000 base pairs upstream and downstream of the transcription start sites of each gene using the *promoter* function from the R ‘GenomicFeatures’ package v.1.38.2 (Lawrence et al., 2013) with parameters: upstream = 2,000 and downstream = 2,000 (Figure S1B).

To select regulatory regions that are related to sites of active transcription in PC, 112,124 DNA accessible elements that were defined as reproducible across Assay for Transposase-Accessible Chromatin sequencing (ATAC-seq) experiments of 26 primary untreated PC tumors were retrieved from the Genomic Data Commons (GDC) Portal (https://gdc.cancer.gov/about-data/publications/ATACseq-AWG) (Corces et al., 2018). Genomic positions of accessible elements were lifted over from hg38 to hg19 reference genome using *liftOver* version 366 (Kent et al., 2002). Only accessible elements in canonical chromosomes were retained. Promoter and enhancer regions were intersected with PC-specific accessible elements with the *intersectBed* command from BEDTools v.2.29.2 (Quinlan and Hall, 2010) using default parameters and only overlapping regions were retained. Candidate enhancer-gene interactions were retained if associated with the related promoter, and enhancer-gene associations in which the enhancer overlapped with the promoter of the same gene were discarded. Interactions smaller than 1 million base pairs were retained for further analyses. Overall, 14,013 promoters and 39,479 and 21,645 enhancer-gene associations for VCaP and LNCaP cells, respectively, were retained as PC-specific accessible elements.

To identify TF binding regions in LNCaP and VCaP cells, significant peak calls (*i.e.* p-value$\leq 10^{-5}$) of 22 chromatin immunoprecipitation sequencing (ChIP-seq) experiments were obtained from ChIP-Atlas (Oki et al., 2018) (Table S1). For each TF and cell line, peaks were positionally sorted and merged with *mergeBed* command from BEDTools v.2.29.2 toolset (Quinlan and Hall, 2010) using default parameters. TF binding regions were intersected with PC-specific accessible elements using the *intersectBed* command (Quinlan and Hall, 2010) with default parameters. Only overlapping regions were retained and considered as active TF binding sites.

To identify genes putatively regulated by each TF, active binding sites were intersected with promoter and enhancer regions using the *intersectBed* command from BEDTools v.2.29.2 toolset (Quinlan and Hall, 2010) with default parameters.

Over-Representation Analysis

The enrichments of genes of interest in specific gene sets (*i.e.* Over-Representation Analysis, ORA) were performed with the *enricher* function in the R package 'clusterProfileR' v.3.18.1 (Yu et al., 2012) using the 186 Kyoto Encyclopedia of Genes and Genomes (KEGG) canonical pathways downloaded with the *msigdbr* function of the R package 'msigdbr'. Enrichment tests with false discovery rate (FDR) $\leq$ 0.1 were considered as significant. Results of over-representation analyses performed in this study are reported in Table S2.

Differentially expressed splicing-related genes

Since SRGs are highly expressed in the cell (de la Grange et al., 2010; Sebestyén et al., 2016), canonical parametric methods for differential expression analysis may fail to detect statistically significant changes in the presence of large gene counts and subtle differences between cohorts (Li and Tibshirani, 2013). Recent studies have shown that nonparametric differential expression analysis approaches are more robust than parametric models to handle this scenario (Shi et al., 2015; Zhu et al., 2019). In this view, to identify FOXA1-regulated SRGs in PC, parametric and non-parametric analyses were performed. Firstly, differentially expressed SRGs were identified by comparing their transcripts per million read distributions between *FOXA1* highly expressing ($\geq 75^{th}$ percentile of expression distribution) and remaining samples with a two-tailed Kolmogorov-Smirnov test. p-values were corrected for multiple tests using the false discovery rate (FDR) by the Benjamini–Hochberg method. To estimate the empirical p-value (emp-pv) of each comparison, a Monte Carlo procedure was implemented. For 10,000 iterations, *FOXA1* highly expressing and remaining samples were randomly selected and, for each SRG, the transcripts per million read distributions were compared using a two-tailed Kolmogorov-Smirnov test. For each SRG, the emp-pv was measured as the proportion of tests with p-value smaller than the observed one over the total number of iterations. Concomitantly, canonical parametric differential expression analyses were performed between *FOXA1* highly expressing and remaining samples using the R packages 'DESeq2' and 'EdgeR' in parallel (Love et al., 2014; Robinson et al., 2010) for primary tumors, for which raw sequencing counts were available. Briefly, read counts of 20,531 genes of each sample were used as input for both DESeq2 and EdgeR. Genes with read count equal to zero across all samples were removed. SRGs with FDR $\leq$ 0.01, emp-pv $\leq$ 0.01, DESeq2 or EdgeR absolute $\log_2$ Fold Change (FC) $\geq$ 0.2 and adjusted p-value $\leq$ 0.01 were considered as differentially expressed in *FOXA1* highly expressing samples as compared to remaining samples. For the SU2C dataset, SRGs with FDR $\leq$ 0.01, emp-pv $\leq$ 0.01, and an absolute $\log_2$(FC) of median transcripts per million $\geq$ 0.2 were considered as altered (Figures S2A–S2C and Table S3).

Cell transfections

Transfections with plasmid DNA and siRNA duplexes (Table S1) were performed as detailed in the figure legends using ViaFect (E4981, Promega) and RNAiMax (13778-075, Thermo Fisher Scientific), respectively, according to the manufacturers' instructions.

Antibodies, plasmids, and oligonucleotides

pcDNA3.1 FOXA1 was provided by Jason Carroll (Cancer Research UK Cambridge Institute). pcDNA3-myc-Flna WT, which encodes *FLNA*Δex30 without exon 30, was a gift from John Blenis (Addgene plasmid # 8982 ; http://n2t.net/addgene:8982 ; RRID:Addgene_8982) (Woo et al., 2004). pcDNA3-myc-Flna+ex30FLNA was generated by mutagenesis using the Q5 Site-Directed Mutagenesis Kit (NEB:E0554S) according to manufacturer's instructions using primers designed in the NEBaseChanger tool (https://nebasechanger.neb.com, Table S1). Correct incorporation of exon 30 was confirmed by Sanger Sequencing (Source Bioscience) and PCR. The following antibodies were used: anti-FOXA1 (ab23738, Abcam), anti-actin (A1978, Sigma-Aldrich), anti-AR (554225, BD Biosciences:), anti-SRSF1 (32-4500, Thermo Fisher Scientific), anti-mouse IgG HRP-linked (P044701-2, Dako), and anti-rabbit IgG HRP-linked (P044801-2, Dako). Sequences used to generate siRNA duplexes are as previously described (Zheng et al., 2015) or commercially-designed (ON-TARGETPlus, Dharmacon Horizon Discovery) and are listed in Table S1. Sequences used to generate oligonucleotide primers for qRT-PCR were designed by entering the Ensembl (http://www.ensembl.org) Transcript ID representing the principal isoform for each gene into the National Center for Biotechnology Information (NCBI) Primer-BLAST tool (https://www.ncbi.nlm.nih.gov/tools/primer-blast) and commercially synthesised (Integrated DNA Technologies). Primer sequences are listed in Table S5. Primers used for endpoint PCR splicing assay were designed in exons flanking *FLNA* exon 30 using http://bioinfo.ut.ee/primer3-0.4.0/ and commercially synthesised (Integrated DNA Technologies). Primers and probes for ddPCR were designed using https://www.primer3plus.com, based on Bio-Rad recommended guidelines at https://www.bio-rad.com/webroot/web/pdf/lsr/literature/Bulletin_6407.pdf and commercially synthesised (Bio-Rad). Probes used for digital droplet PCR were designed to specifically recognize either the FLNA+ex30 (FAM, spanning exons 30–31) or FLNAΔex30 (HEX, spanning exons 29–31), with primers in exons flanking the targeted exon (forward primer in exon 29 and reverse primer in exon 31). All primer and probe sequences are reported in Table S1.

SDS-PAGE and Western blotting

Whole cell lysate protein samples were obtained by lysis of cells in RIPA (Radio-Immunoprecipitation Assay) buffer for 30 minutes at 4°C followed by lysate clearing by centrifugation. Protein concentration was calculated using the bicinchoninic acid (BCA) assay (10678484, Thermo Fisher Scientific) method and samples adjusted to equal concentrations of total protein. Samples were denatured in a 2-Mercapto-ethanol-based SDS sample buffer. Proteins were then separated by SDS-PAGE on 12% w/v Tris gels, transferred onto PVDF (polyvinylidene difluoride) membrane (000000003010040001, Sigma-Aldrich) using the wet transfer method,

blocked in 5% milk in TBST (Tris-Buffered Saline and Polysorbate 20) for 1 hour at room temperature and then placed in primary antibodies diluted in 5% BSA (Bovine Serum Albumin) in TBST overnight at 4°C. Membranes were washed and incubated with relevant HRP-conjugated secondary antibodies for 1 hour at room temperature. For signal detection, membranes were washed and incubated for 3 minutes each in Luminata Crescendo Western HRP substrate (10776189, Thermo Fisher Scientific) before bands were visualised on the Amersham Imager 600 chemidoc system (29-0834-61, GE Healthcare). Antibody concentrations were as follows: anti-FOXA1 (1:1000), anti-actin (1:100,000), anti-AR (1:1000), anti-SRSF1 (1:500); HRP-linked secondaries (1:5000). Where indicated, densitometric assessments of protein bands were performed using Image Studio Lite v.5.2 (LI-COR), and signal intensities used to calculate relative normalised fold-change (FC) in protein expression (Table S3).

Generation of RNA-seq libraries

Cells were lysed in Tri Reagent Solution (AM9738, Invitrogen) and RNA extracted by phase separation using 1-bromo-3-chloropropane. To exclude genomic contamination, total RNA was treated with DNAse I and cleared with RNA Clean and Concentrator (R1013, Zymo Research). RNAs were quantified using the Qubit 4 Fluorometer (Q33238, Thermo Fisher Scientific). RNA quality was determined using the RNA 6000 Nano kit (5067-1511, Agilent Technologies) on the 2100 Bioanalyzer Instrument (G2939BA, Agilent Technologies). RNA samples with an RNA integrity number >7 were selected for library preparation. RNA-seq libraries for VCaP and PC3 were generated from 1 μg of RNA using the TruSeq total RNA (RS-122-2001, Illumina) and TruSeq stranded mRNA (20020594, Illumina) Library Prep kits, respectively, according to manufacturer's recommendations. VCaP libraries were sequenced on the NextSeq500 (Illumina) in a paired-end manner with a read length of 75 nucleotides (nt). PC3 libraries were sequenced on the NovaSeq6000 (Illumina) in 100nt-long paired-end read modality.

Gene expression analyses of RNA-seq data

Raw sequencing reads were aligned to the human genome reference GENCODE GRCh37 version 28 (Frankish et al., 2019) using STAR (v. 2.7.3a) (Dobin et al., 2013) in two-pass mode (–peOverlapNbasesMin = 40 and –peOverlapMMp = 0.8). Read counts, at the gene level, were estimated using featureCounts (Subread v. 2.0.0) (Liao et al., 2014) with -p, -B and -s 2 parameters. Fragment counts were finally normalized as transcripts per million reads. Hierarchical clustering and principal component analyses of gene expression normalized data showed that samples were appropriately separated upon silencing conditions (Figures S2F and S2G). The R package 'DESeq2' was used to quantify differential expression (Love et al., 2014) between FOXA1 siRNA-treated and control samples to match the contribution of high *FOXA1* expression in primary PCs. Genes with an adjusted p-value<0.1 were considered as differentially expressed (Table S3).

Quantitative reverse transcription PCR

Total RNA was isolated from cells and reverse transcribed to cDNA using a high capacity cDNA reverse transcription kit (4368814, Applied Biosystems). Reactions were performed using 20ng of cDNA per condition combined with forward and reverse primers (Table S1), and SYBR green master mix (NEB: M3003) in a 10ul reaction volume. Assays were performed in the QuantStudio 5 Real-Time PCR system (A34322, Thermo Fisher Scientific) measuring binding of SYBR green to DNA, with ROX as a passive dye. Reaction conditions were as follows: 2 minutes at 50°C, 10 minutes at 95°C, and 40 cycles of 15 seconds at 95°C and 1 minute at 60°C. Cycle threshold (CT) values were calculated using QuantStudio Design and Analysis Software v1.5.1 (Thermo Fisher Scientific). Relative gene expression was determined by the $2^{-\Delta\Delta CT}$ method using the geometric mean expression of two validated endogenous control genes (*ACTB* and *B2M*) to ensure the reliability and reproducibility of observed effects (Table S3).

Alternative splicing analysis of primary PC

The publicly available catalogue of alternative splicing (AS) events was obtained from the GDC portal (https://gdc.cancer.gov/about-data/publications/PanCanAtlas-Splicing-2018) for 384 TCGA primary PC samples. This atlas included five categories of AS events: Cassette Exon (CE), Alternative 3' (A3) and 5' (A5), Intron Retention (IR) and Mutually Exclusive exons (MEX). The percent spliced in (psi or Ψ) value was used as a measure of splicing event inclusion in the mature mRNA (Venables et al., 2009). AS events with (*i*) available information in more than 75% of the samples (Li et al., 2017), (*ii*) mean (μ) Ψ ranging from 0.01 and 0.99 (*i.e.* not constitutively excluded or included, respectively), and (*iii*) in genes with less than 500 events were retained for further analysis. For each selected AS event, missing values were replaced by the mean of the corresponding Ψ distribution across samples (Li et al., 2017).

For each AS event the mean (μ) and standard deviation (s.d. or σ) of Ψ levels in *FOXA1* highly expressing and remaining samples were calculated (Figure S5A). The difference in the μ and σ of the Ψ levels (*i.e.* $\Delta\mu(\Psi)$ and $\Delta\sigma(\Psi)$) between the two groups was then measured. To identify AS events associated with *FOXA1* high expression, events with negligible changes in $\Delta\mu(\Psi)$ and $\Delta\sigma(\Psi)$ were discarded based on the quantile distributions of $\Delta\mu(\Psi)$ and $\Delta\sigma(\Psi)$. In particular, an AS event was retained either (*i*) if $\Delta\mu(\Psi)$ was lower or greater than the 15th or the 85th percentile of $\Delta\mu(\Psi)$ distribution, respectively, or (*ii*) if $\Delta\sigma(\Psi)$ was lower or greater than the 20th or the 80th percentile of $\Delta\sigma(\Psi)$ distribution, respectively. To select AS events that were significantly differentially included between *FOXA1* highly expressing and remaining samples, two non-parametric statistical tests were performed. For each AS event, $\Delta\mu(\Psi)$s between *FOXA1* highly expressing and remaining tumors were tested using a two-tailed Wilcoxon Rank Sum test, whereas $\Delta\sigma(\Psi)$s were compared using a two-tailed Fligner-Killeen test (Saraiva-Agostinho and Barbosa-Morais, 2019). p-values were corrected for multiple testing using the Benjamini–Hochberg procedure. To calculate the emp-pv of each comparison, sample labels were shuffled

1,000 times and at each iteration the two tests were performed. To account for the sample size difference between the *FOXA1* highly expressing and remaining groups, the latter was randomly down-sampled to reach the size of the former for 1,000 times. At each iteration, tests were performed. The success rate (SR) was then computed as the proportion of significant results (p-value<0.05) over the total number of comparisons. AS events with an FDR<0.05, emp-pv<0.05 and SR>0.7 for at least one test were considered as significantly differentially included between *FOXA1* highly expressing and remaining samples and named as FOXA1-regulated AS events. AS events with non-statistically significant changes were considered not differentially spliced by FOXA1 and termed as FOXA1-unregulated AS events (Table S4).

Cumulative distributions of the number of FOXA1-regulated AS events with positive and negative splicing changes (*i.e.* $\Delta\mu(\Psi)$ and $\Delta\sigma(\Psi)$) were calculated starting from the mean inclusion level of 0.5 (*i.e.* mixed isoform population) to the boundaries of 0 and 1 (*i.e.* dominant isoform population). Monte Carlo simulations (1,000 iterations) were used to measure the empirical cumulative distribution of the number of exons with inclusion changes. For each iteration, the direction of the inclusion change (i.e. positive or negative) of FOXA1-regulated AS events was randomly assigned and the number of exons with positive and negative changes at each mean inclusion levels were annotated. At the end of all iterations, the cumulative distribution of the average expected number of AS events with splicing changes, as well as its confidence intervals, were calculated.

Alternative splicing analysis of cell lines

AS events were identified using Whippet v0.11 (Sterne-Weiler et al., 2018) on AR^+ VCaP and AR^- PC3 RNA-seq data. The GENCODE GRCh37 version 28 (Frankish et al., 2019) was employed as reference. The event index reference was generated using –suppress-low-tsl and –bam parameters to allow the identification of unannotated splice-sites and exons from each alignment BAM file. Core exons, alternative acceptor splice sites, alternative donor splice sites, retained introns, alternative first exons and alternative last exons identified by Whippet were retained for further analyses as matching the corresponding AS event classes (*i.e.* CE, A3, A5 and IR) defined for primary tumors. AS events with a Whippet confidence interval width ≥ 0.2 in at least one sample were filtered out from the analysis (Sterne-Weiler et al., 2018). AS events with splicing complexity higher than K0, probability$\geq$0.9 and $|\Delta\mu(\Psi)|$ >0.05 were considered as differentially spliced and termed as FOXA1-regulated AS events (Van Nostrand et al., 2020b). For each set, not-significant AS events were retained as controls and termed FOXA1-unregulated AS events.

Nonsense-mediated decay determinant exons

Genomic positions of a previously defined list of 15,518 nonsense-mediated decay (NMD) determinant cassette exons were retrieved and stratified into premature termination codon (PTC) introducing (PTC-introducing) and preventing (PTC-preventing) ones accordingly to the definition of poison and essential events, respectively (Pervouchine et al., 2019). Coordinates of these events were intersected with those of primary PC cassette exon events using *intersectBed* command from BEDTools v2.29.0 toolset (Quinlan and Hall, 2010) with default parameters and cassette exons were annotated accordingly (Table S4).

For RNA-seq data of VCaP and PC3 cell lines, sensitivity to NMD for transcripts harboring cassette exon events was measured using the *predictNMD* function in the R 'notNMD' package. Events with a difference of NMD probability between transcripts, including and excluding the exon below the 15th percentile, were defined as putative PTC-preventing exons, while events with a difference of NMD probability above the 85th percentile were defined as putative PTC-introducing exons, for a total of 16,880 putative NMD-determinant cassette exons (see key resources table for deposited data).

Splicing-associated chromatin signatures

Genomic coordinates of AS events in primary PC and exons marked by splicing-associated chromatin signatures (SACS) (Agirre et al., 2021) were intersected using the *findOverlaps* function of the R 'GenomicRanges' package (Lawrence et al., 2013) and cassette exons with a minimum reciprocal overlap of 90% were considered as marked by chromatin signatures (Table S4). Enrichment of SACS-marked cassette exons in the FOXA1-regulated set with respect to controls was assessed with a two-tailed Fisher's exact test.

Splicing code analysis

RNAmotifs (Cereda et al., 2014) was used to identify *cis*-acting multivalent RNA motifs of 4nt length (*i.e.* tetramers), among 512 degenerate and non-degenerate motifs, that occurred in a specific AS region more often in cassette exons of interest compared to 3,266 FOXA1-unregulated exons with $\mu_{PC}(\Psi)$>0.9 or $\mu_{PC}(\Psi)$<0.1 defined as controls. The tool was run considering three enrichment regions (R): (*i*) R_1 [-205:-5] nucleotides of intronic sequence upstream of the 3′ splice site; (*ii*) R_2 corresponding to the entire exonic sequence (or up to 200 nt from both splice sites in case of exon longer than 400 nt); and (*iii*) R_3 [10:210] nucleotides of intronic sequence downstream of the 5′ splice site. RNAmotifs empirical p-values were calculated using 10,000 bootstrap iterations. Tetramers with RNAmotifs Fisher's p-values $\leq$ 0.05 (or the 1st percentile of the p-value distribution in case of highly significant results) and empirical p-values $\leq$ 0.0005 were considered as enriched and retained for further analysis (Table S5). For enriched tetramers, RNAmotifs was run performing a position-specific enrichment analysis at exon/intron junctions of alternative CEs and flanking exons extending 1,000 and 50 nucleotides into introns and exons to generate the corresponding RNA splicing map.

To select *trans*-acting SRGs that were most likely to bind the enriched tetramers, a list of 466 11-nt long position weight matrices (PWMs) derived from HepG2 eCLIP data for 62 SRGs was collected from the mCross database (Feng et al., 2019). For each enriched

tetramer, a PWM was computed on tetramer occurrences at regulated exons extending both tetramer sides of two nucleotides. Similarities between tetramer and mCross PWMs were calculated using the MACRO-APE tool (Vorontsov et al., 2013) with parameters –position J,direct with J = −3,-2,-1,0 to allow up to four different alignments to the most informative seven core positions of the mCross PWM (Feng et al., 2019). For each tetramer and SRG pair, the highest similarity amongst the four alignments (ω) was retained (Table S5). In case of multiple mCross PWMs for the same SRG, the different similarity values were averaged. Hence, the similarity value Ω was measured as follows:

$$\forall \; SRG \;\; and \;\; tetramer : \Omega = \frac{\sum_{i=1}^{N_{PWM}} \omega_i}{N_{PWM}}$$

where ω_i is the similarity value between the tetramer and the ith mCross PWM o and is the total number of mCross PWMs of a SRG. Ω was named “sequence similarity score”.

Next, the similarity between profiles of the RNAmotifs maps of each enriched tetramer and those of eCLIP-based RNA splicing maps of the 62 SRGs was assessed. Firstly, cross-linking sites, as iCounts peak instances, from eCLIP experiments in HepG2 cells for each SRG were collected (König et al., 2010). Then, for each tetramer, eCLIP-based splicing maps of all SRGs were generated around exons with tetramer instances (*i.e.* extending 1,000 and 50 nucleotides into introns and exons). At each position, and for each SRG, a cross-linking enrichment score was computed by performing a Fisher’s exact test comparing the proportion of FOXA1-regulated and constitutive exons having at least one iCounts peak:

$$CES = -2\log(p)$$

where p is the p-value of the Fisher’s exact test.

The similarity between the RNAmotifs and eCLIP-based RNA splicing maps was then evaluated by calculating the Bhattacharyya coefficient (BC) (Rizzo et al., 2019) as follows:

$$BC(q,t) = \sum_{i=1}^{n} \sqrt{q_i t_i}$$

where q_i is the RNAmotifs enrichment score of the tetramer at position i on the map and t_i is the cross-linking enrichment score of the SRG at the same position i, and n is the length of the maps.

Finally, for each tetramer and SRG a global Matching Score was computed as the product of the sequence similarity score Ω and the map similarity given by the Bhattacharya coefficient:

$$\forall \; SRG \;\; and \;\; tetramer : \; Matching \; Score = \Omega \cdot BC$$

SRGs with Matching Score $\geq 75^{th}$ percentile of its distribution were considered as significantly associated with the corresponding tetramer (Figure S5E).

Sequence logos were plotted with the *ggseqlogo* function of the R ‘ggseqlogo’ package.

Survival analysis

Clinical data for 332 primary PC patients were obtained from the TCGA Data Matrix portal (Level 3, https://tcga-data.nci.nih.gov/tcga/dataAccessMatrix.htm). Disease-free survival was defined as the time between primary treatment and the diagnosis of disease progression, as defined by biochemical or clinical recurrence, or the end of follow-up. PTC-introducing and PTC-preventing FOXA1-regulated cassette exons were divided into inhibited and enhanced events according to their $\Delta\mu(\Psi)$ sign upon *FOXA1* high expression, resulting into four groups (*i.e.* inhibited PTC-introducing, enhanced PTC-introducing, inhibited PTC-preventing and enhanced PTC-preventing FOXA1-regulated cassette exons). As previously proposed (Thomas et al., 2020), for each group and each patient, the following S statistic was computed:

$$S = n_{25} \div n_{75}$$

where n_{25} and n_{75} are the number of events with $\Psi \leq 25^{th}$ and $\geq 75^{th}$ percentiles, respectively, of their inclusion distribution across patients.

For each group of FOXA1-regulated exons, patients were stratified into high and low expressors based on the 25th and 75th percentile of the S statistics distribution, respectively. Exploiting this stratification, survival analysis was performed by fitting a univariate Cox proportional hazards model with log-rank test (Therneau and Grambsch, 2000) using the *coxph* function in the R ‘survival’ package.

Similarly, to assess the contribution of each NMD-determinant FOXA1-regulated cassette exon on disease-free survival, patients were stratified according to the 25th and 75th percentiles of the Ψ level distribution of each event and survival analysis was performed as described above. Log-rank test p-values were corrected for multiple testing with the Benjamini–Hochberg procedure. FOXA1-regulated cassette exons with FDR<0.05 were selected as the strongest survival-associated candidates (Table S4).

The optimal prognostic cutpoint of *FLNA* exon 30 Ψ inclusion level in primary PCs was identified using the *surv_cutpoint* function from the R ‘survminer’ package (Lauria et al., 2020; Lausen and Schumacher, 1992). All Kaplan-Meier curves were generated using the *survfit* and *ggsurvplot* functions of the R ‘survival’ package.

Splicing assays

Cells were lysed in TRI Reagent (AM9738, Invitrogen) and RNA extracted by phase separation using 1-bromo-3-chloropropane. RNA was DNAse treated to remove contaminating genomic DNA and transfected plasmid DNA. cDNA was generated from RNA using a high capacity cDNA reverse transcription kit (4368814, Applied Biosystems).

For endpoint PCR splicing assay, primers flanking the variable exon 30 within *FLNA* (Table S1) were combined with cDNA, dNTPs and Taq Polymerase (NEB, M0273) in standard reaction buffer. PCR reactions were performed in a ProFlex thermocycler (4484075 Applied Biosystems) with 30 cycles of amplification, to determine endogenous exon 30 inclusion, and a 53°C annealing temperature. An additional reconditioning PCR for 3 cycles of amplification was undertaken using 2ul of the first PCR product (Thompson et al., 2002). PCR products were detected and quantified using the QIAxcel DNA High Resolution Kit (1200) (929002, QIAGEN) with the QIAxcel Advanced System capillary electrophoresis device (9002123, QIAGEN). The Ψ value was used as a measure of exon 30 expression (Venables et al., 2009) (Table S6).

Digital droplet PCR (ddPCR) was performed using the QX200 Droplet Digital PCR System (1864001, Bio-Rad). Droplets were generated using the QX200 Droplet Generator (1864002, Bio-Rad) in a total volume of 20μL containing cDNA corresponding to 20 ng of input RNA, 900nM/250nM final concentration of *FLNA* exon 30 primers/probe (Table S1), and 10μL of 2X ddPCR Supermix for Probes (No dUTP) (1863024, Bio-Rad). PCR reactions were executed according to the manufacturer's instructions as follows: enzyme activation at 95°C for 10 min (1 cycle), denaturation at 94°C for 30s followed by annealing/extension at 55°C for 1 min (40 cycles), enzyme deactivation at 98°C for 10 min (1 cycle), and hold at 4°C. After PCR completion, droplets were processed with the QX200 Droplet Reader (1864003, Bio-Rad) and analyzed using QuantaSoft software (1864011, Bio-Rad). Total events in each sample replicate were quantitated using the mean copy number per μl (Table S6).

Cell viability and colony formation assays

Cell growth assays were performed using (3-(4,5-Dimethylthiazol-2-yl)-2,5-Diphenyltetrazolium Bromide) (MTT) (L11939.06, Alfa Aesar) according to the manufacturer's instructions. Briefly, 2000 PC3 cells were seeded into each well of a 96-well plate and grown to ~20–30% confluence prior to transfection with 100ng of pcDNA3.1-VO, pcDNA3-myc-Flna+ex30FLNA or pcDNA3-myc-Flna using Viafect (E4981, Promega). After 72 hours, MTT was added to each well to a final concentration of 0.67 mg/mL and incubated at 37°C, 5% CO_2 in a humidified incubator for 2 h. Subsequently, MTT reagent was removed, 100μL dimethyl sulfoxide (DMSO) (10213810, Thermo Fisher Scientific) was added to each well and agitated at room temperature for 15 mins. Absorbance was measured at 560nm and 630nm using the SpectraMax Plus384 microplate reader (Molecular Devices), and normalized by subtracting the 630nm value from the 560nm value, and percentage viability was calculated as: the treatment absorbance divided by the DMSO control absorbance. All data were normalized to a vector only control (Table S6).

For colony formation assays, 200,000 PC3 cells were seeded in each well of a six well plate. Cells were transfected with 2ug of pcDNA3.1-VO, pcDNA3-myc-Flna+ex30FLNA or pcDNA3-myc-Flna using Viafect (E4981, Promega). After 48 hours, cells were trypsinized and counted, and 300 cells per condition were seeded into 6 well plates (three technical replicates per condition). Cells were then grown for eight days to allow the formation of visible colonies. Media was removed, cells were washed 3x in PBS and then colonies were fixed using 100% methanol. Methanol was removed and crystal violet solution (0.05% w/v in H_2O) was added to the plates (C0775, Sigma-Aldrich). After 40 minutes, excess crystal violet was removed and plates were washed with H_2O. Plates were imaged on the Amersham Imager 600 chemidoc system (29-0834-61, GE Healthcare). Images were analysed using ImageQuantTL (GE Healthcare) to accurately count the number of colonies in each condition. Crystal violet stain was dissolved by addition of 1mL 2% Triton X-100 to each well and agitation for 4 hours. Three x 200ul from each well was transferred to a clear bottom 96 well plate and absorbance measured at 405nm and 560nm using the SpectraMax Plus384 microplate reader (Molecular Devices), and normalised by subtracting the 405nm value from the 560nm value (Table S6).

For both functional assays, *FLNA* exon 30 expression was confirmed by endpoint PCR splicing assays as described above using primers flanking the variable exon 30 within *FLNA* (Table S1) with 25 cycles of amplification. PCR products were resolved through 3% agarose gel in TBE (Tris-Borate-EDTA) containing GelRed DNA dye (41003, Biotium), imaged using the G-Box (Syngene), and analysed using Image Studio Lite v.5.2 (LiCoR).

Assessment of tumor purity constraints

The PC tumour microenvironment contains multiple cell types including benign basal and luminal epithelial cells, stromal cells, and infiltrating immune cells (Bahmad et al., 2021). This cellular intratumoral heterogeneity may bias analysis of bulk sequencing data (Aran et al., 2015). To assess this issue, tumor purity estimates for primary PC samples were retrieved from Aran et al. (Aran et al., 2015) (Figure S6A). Samples was stratified the cohort into "high purity" (i.e. purity ≥90%) and "low purity" (i.e. purity <90%) tumors (Figure S6B). Multivariate covariance analysis of cumulative SRG and TF expression, assessment of differentially expressed SRGs, and evaluation of alternatively splicing events were performed for both cohorts as described above (Figures S6C–S6G).

Orthogonally, batch-corrected expression data (*i.e.* TPMs) of 349 primary PCs and 107 benign prostate tissues were deconvoluted using the xCell algorithm (Aran et al., 2017) into cell-type scores that recapitulate the enrichment of distinct cell types. The resulting infiltrate-specific scores were used to investigate the possible contribution of immune, stromal, or benign epithelial cells to PC transcriptomes (Figures S6H–S6K).

Finally, to assess whether infiltration levels higher than those observed in primary PCs could affect the identification of TFs regulating SRG expression, a Monte Carlo simulation was implemented using batch-corrected normalized TPMs for 195 high purity (i.e. purity $\geq$90%) primary PCs and 107 benign prostate samples. Artificial gene expression profiles were generated by merging complementary fractions of cancer and benign transcriptomes to simulate ten increasing levels of tumor purity *p* (ranging from 10% to 100%). For each *p*, the expression (TPM) of genes *g* in a tumor sample *i* was calculated as follows:

$$\forall p : g_i = \left(g_i \times \frac{p}{100}\right) + \left(g_{benign} \times \frac{(1 - p)}{100}\right)$$

where g_{benign} is the TPM value of *g* in a randomly selected benign prostate transcriptome. This procedure was repeated for 100 times randomly selecting benign prostate samples. For each iteration, multivariable covariance analysis of cumulative SRG and TF expression was performed as describe above. For each purity level, the mean and standard deviation of the contribution of each TF to the coefficient of determination (R^2) of the model were computed across the 100 iterations. Additionally, for each iteration and purity level, differentially expressed SRGs upon FOXA1 high expression were identified as described above. For each SRG, a success rate (SR) was defined as the number of times the gene was differentially expressed across the 100 iterations (Figures S6L and S6M).

QUANTIFICATION AND STATISTICAL ANALYSIS

All statistical details including the statistical tests used, p-value indications, number of experiments and dispersion and precision measures can be found in the figures, figure legends or in the results. Graphical data of *in vitro* experiments represent the mean $\pm$ standard error of the mean (SEM) of independent experiments and the two-tailed independent sample T-test was employed to identify differences between groups with p-value < 0.05 taken to indicate statistical significance. The two-tailed Wilcoxon Rank Sum test was used to compare distributions and the two-tailed Fisher's exact test was used to compare proportions across conditions. All statistical tests were performed using the R software (v.3.5.2).

Cell Reports, Volume *40*

Supplemental information

FOXA1 regulates alternative
splicing in prostate cancer

Marco Del Giudice, John G. Foster, Serena Peirone, Alberto Rissone, Livia Caizzi, Federica Gaudino, Caterina Parlato, Francesca Anselmi, Rebecca Arkell, Simonetta Guarrera, Salvatore Oliviero, Giuseppe Basso, Prabhakar Rajan, and Matteo Cereda

Figure S1. Transcriptional regulation of SRGs in PC, related to figure 1.

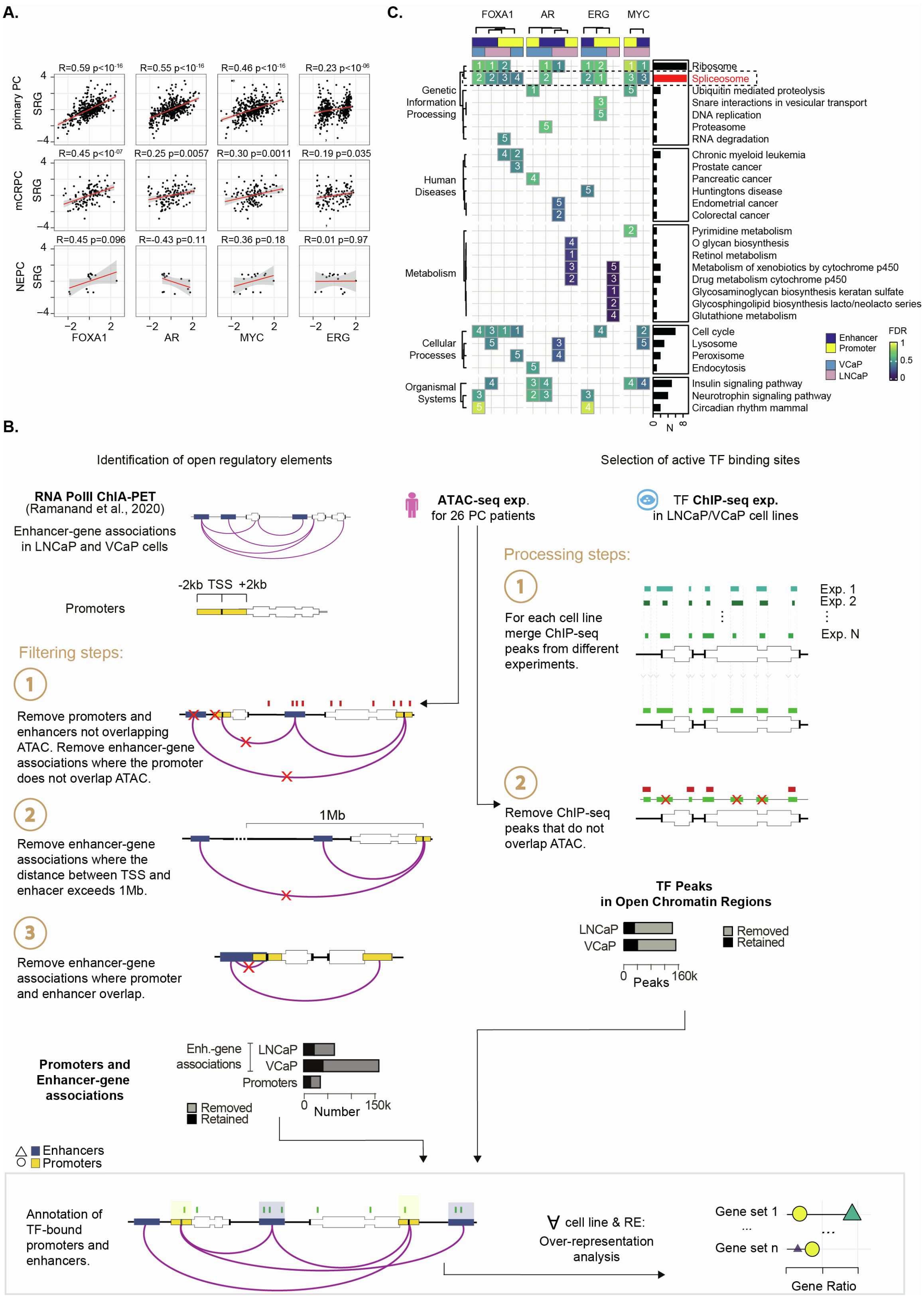

Figure S1. (A) Scatter plots between the scaled expression of each TF and the cumulative expression of SRGs in primary PCs (top), mCRPCs, (middle), and NEPCs (bottom). Pearson's correlation and corresponding p-value are shown for each plot. (B) Schematic representation of the pipeline used to identify active TF binding sites and their over-

representation in gene sets. (C) Over representation analysis performed on genes with active TF binding sites in KEGG pathways. Top five most significant gene sets are shown for each condition. Cell color indicates the statistical significance (FDR) and numbers indicate the rank of the gene set. Bar plots on the right show the number (N) of conditions in which the gene set is significantly enriched.

Figure S2. SRG differential expression analyses, related to figure 1.

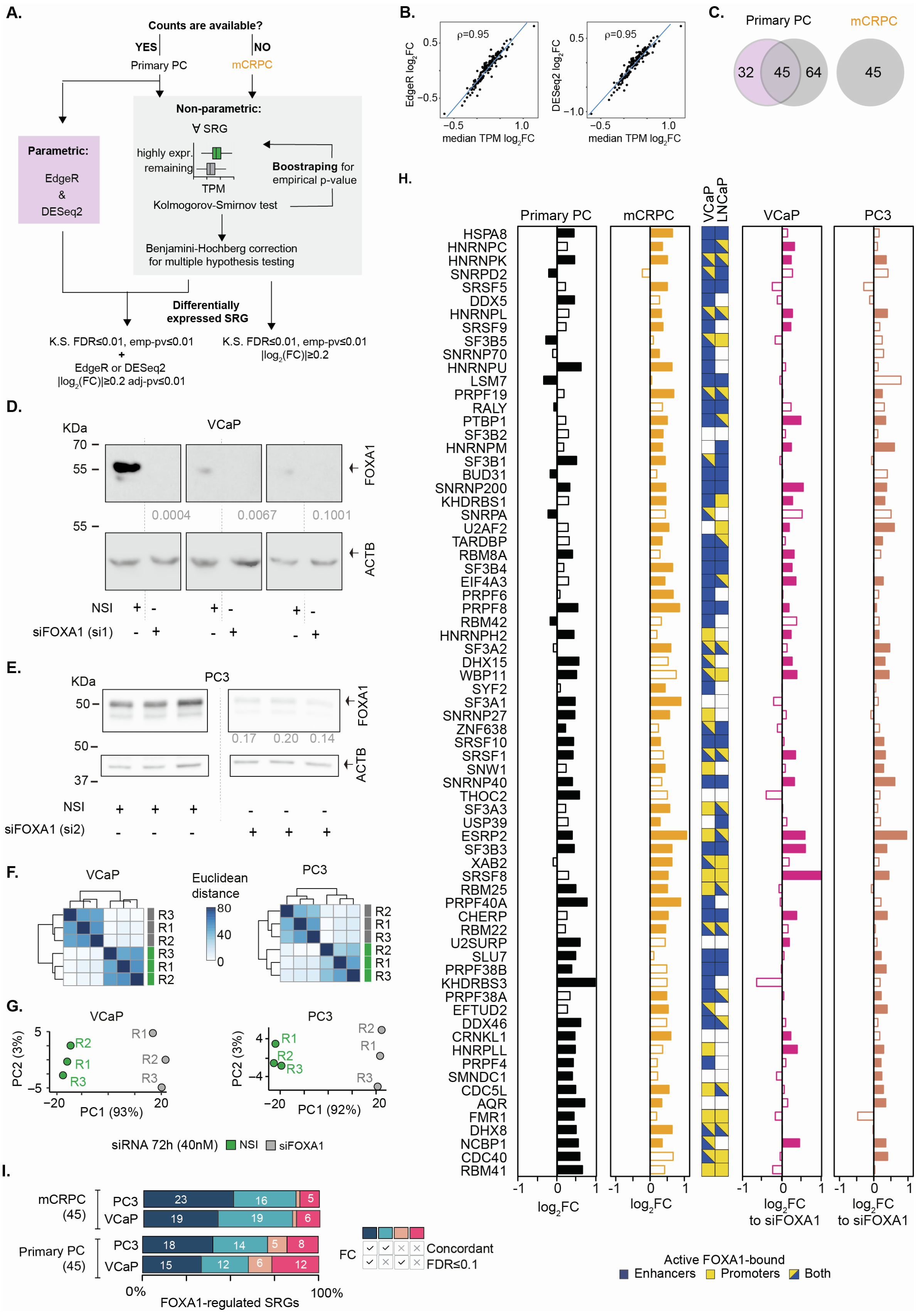

Figure S2. (A) Schematic representation of the pipeline used to detect SRGs that are differentially expressed (DE) in primary PCs and mCRPCs upon high *FOXA1* expression. (B) Scatter plots showing the correlation between median log_2 Fold Change (FC) and FC measured by EdgeR (right) or DESeq2 (left) for the SRGs set. The value of Pearson's correlation coefficient (r) is reported. (C) Venn diagrams showing the number of SRGs identified as DE by the parametric (pink) and non-parametric (grey) approaches. (D,E) Representative Western blotting images of whole cell lysates from (D) VCaP and (E) PC3 cells used for RNA-seq analysis upon depletion of FOXA1 with one siRNA duplex (si1 or si2, 40nM, or 20nM, respectively for 72 hours) using antibodies to FOXA1 and ACTB. ACTB-normalised protein expression compared to control, calculated by densitometric band quantitation, are shown below the upper blot image. (F) Hierarchically clustered heatmaps of Euclidean distance between expression values for VCaP and PC3. (G) Scatter plot of the first two components of principal component analysis for VCaP and PC3 datasets. Percentage of variance explained by each component is reported on each axis. (H) FC in TPMs of 71 FOXA1-regulated SRGs between FOXA1 highly expressing and remaining primary PCs (black) and mCRPCs (orange). Filled bars indicate significant expression changes. Central heatmap annotation indicates the presence of active FOXA1 binding sites on SRG promoters and/or cognate enhancers. Bar plots on the right indicate FC in expression levels upon FOXA1 depletion (*i.e.* NSI versus siFOXA1) of each SRG in VCaP (magenta) and PC3 (brown) cells. Filled bars indicate significant expression changes. (I) Bar plots showing the fractions of FOXA1-regulated SRGs in primary PCs and mCRPCs that changed in expression upon FOXA1 depletion in VCaP and PC3 cells. Absolute number of FOXA1-regulated SRGs are reported. In total, 23 and 35 SRGs were concordantly regulated by FOXA1 in both cell lines to a similar magnitude to primary PCs and mCRPCs. Consistent with the metastatic origin of the cell lines, a higher number of differentially expressed SRGs in mCRPCs were concordantly up-regulated in the cell lines than the SRGs that were DE in primary PC. On average, 76% of the FOXA1-regulated SRGs in primary PCs and/or mCRPCs were concordantly regulated by FOXA1 in the two cell lines

Figure S3. Transcriptional architecture of HNRNPK, HNRNPL, SRSF1, related to figure 1.

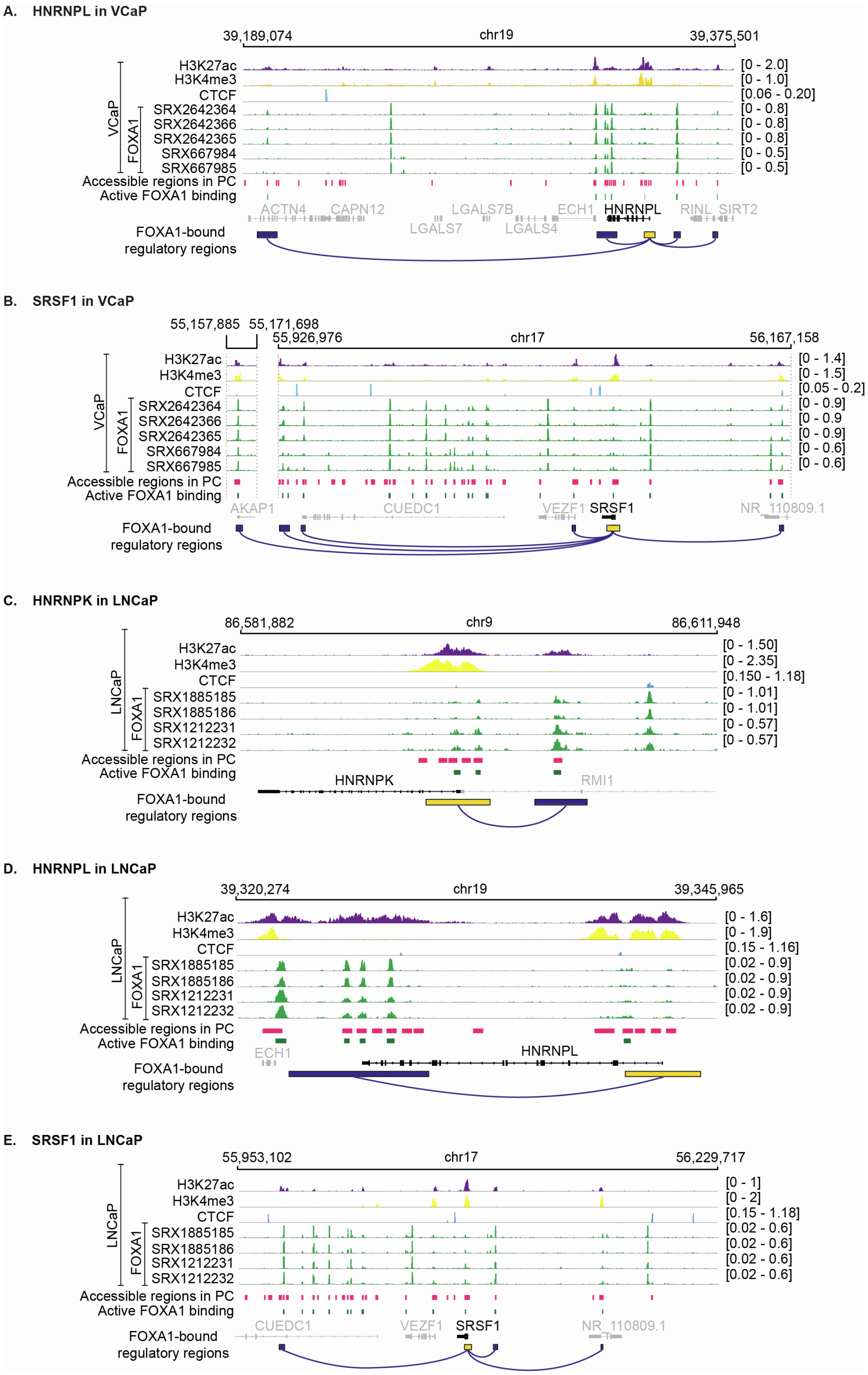

Figure S3. For each panel, ChIP-seq density read tracks of H3K27ac, H3K4me3, CTCF (2 overlayed experiments for VCaP) and FOXA1 (5 and 4 experiments for VCaP and LNCaP, respectively) are shown with recurrent accessible regions of primary PC from ATAC-seq experiments, active FOXA1 binding sites, and RNA PolII ChIA-PET-derived FOXA1-bound promoters (yellow) and cognate enhancers (blue).

Figure S4. ***In vitro*** **validations in PC cell lines, related to figures 1, 5 and 6.**

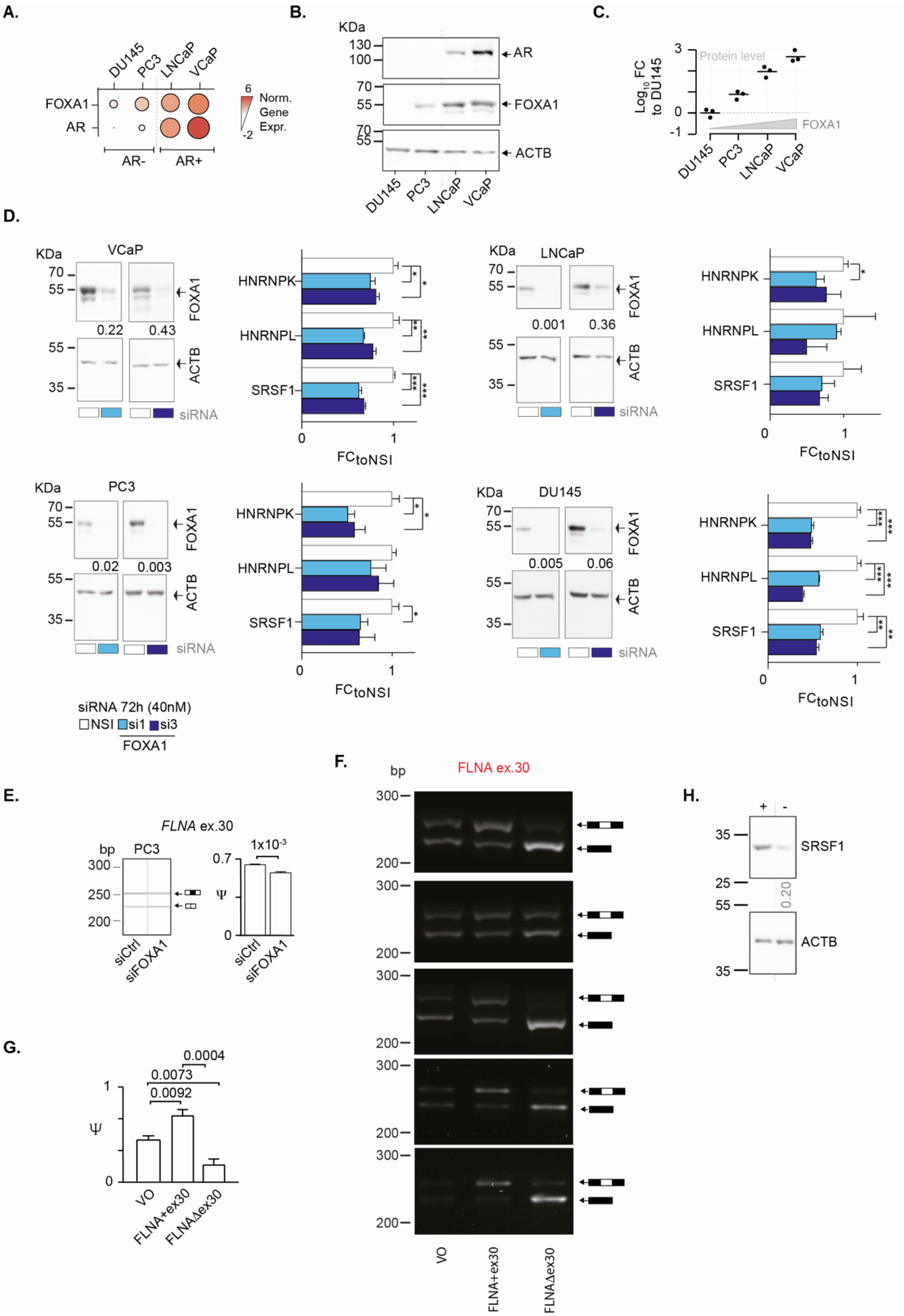

Figure S4. (A) *FOXA1* and *AR* normalized gene expression levels in four PC cell lines as measured in Cancer Cell Line Encyclopedia RNA-Seq data. (B) Representative Western blotting images of whole cell lysates from PC cell lines using

antibodies to AR, FOXA1 and ACTB. (C) Densitometric band quantitation results showing the mean log_{10} relative normalized FC in FOXA1 protein expression relative to DU145 cells (three independent experiments). Consistent with Cancer Cell Line Encyclopedia RNA-Seq data, we identified the highest and lowest levels of FOXA1 protein expression in the VCaP cells and DU145 cells, respectively. (D) Representative Western blotting images of whole cell lysates from PC cell lines (left panel) upon FOXA1 depletion with the two siRNA duplexes (si1 or si3, 40nM for 72 hours) using antibodies to FOXA1 and ACTB. We employed one siRNA duplex used for RNA-seq plus a further independent duplex to reduce artifactual hits. ACTB-normalised protein expression compared to control, calculated by densitometric band quantitation, is shown below the upper blot images. Bar plots (right panel) depict the mean (± standard error) expression changes of candidate SRGs measured by qRT-PCR upon FOXA1 depletion from biological triplicate samples. Stars *, **,*** depict p-values ≤0.05, 0.01 and 0.001, respectively. (E) *FLNA* exon 30 inclusion changes in PC3 cells were measured by endpoint PCR splicing assays upon FOXA1 depletion with one siRNA duplex (si1, 40nM for 72 hours). Two-tailed T-test was used to compare conditions. (F-G) Total (endogenous and exogenous) *FLNA* exon 30 expression in PC3 cells was measured by endpoint PCR assays following transfection with 2 μg of plasmid DNA vector encoding FLNA with or without exon 30 (*i.e.* FLNA+ex30 or FLNAΔex30, respectively, or vector only (VO) control). (F) Agarose gel electrophoresis images show two bands representing *FLNA* transcripts including or excluding exon 30 which were quantified to determine Ψ. (G) Two-tailed T-test was used to compare the five biological replicates. (H) Representative Western blotting images of whole cell lysates from PC3 cells upon SRSF1 depletion with one siRNA duplex (40nM for 72 hours) using antibodies to SRSF1 and ACTB. ACTB-normalised protein expression compared to control (NSI), calculated by densitometric band quantitation, is shown below the SRSF1 blot image.

Figure S5. Analyses of FOXA1-mediated AS regulation, related to figures 2-5.

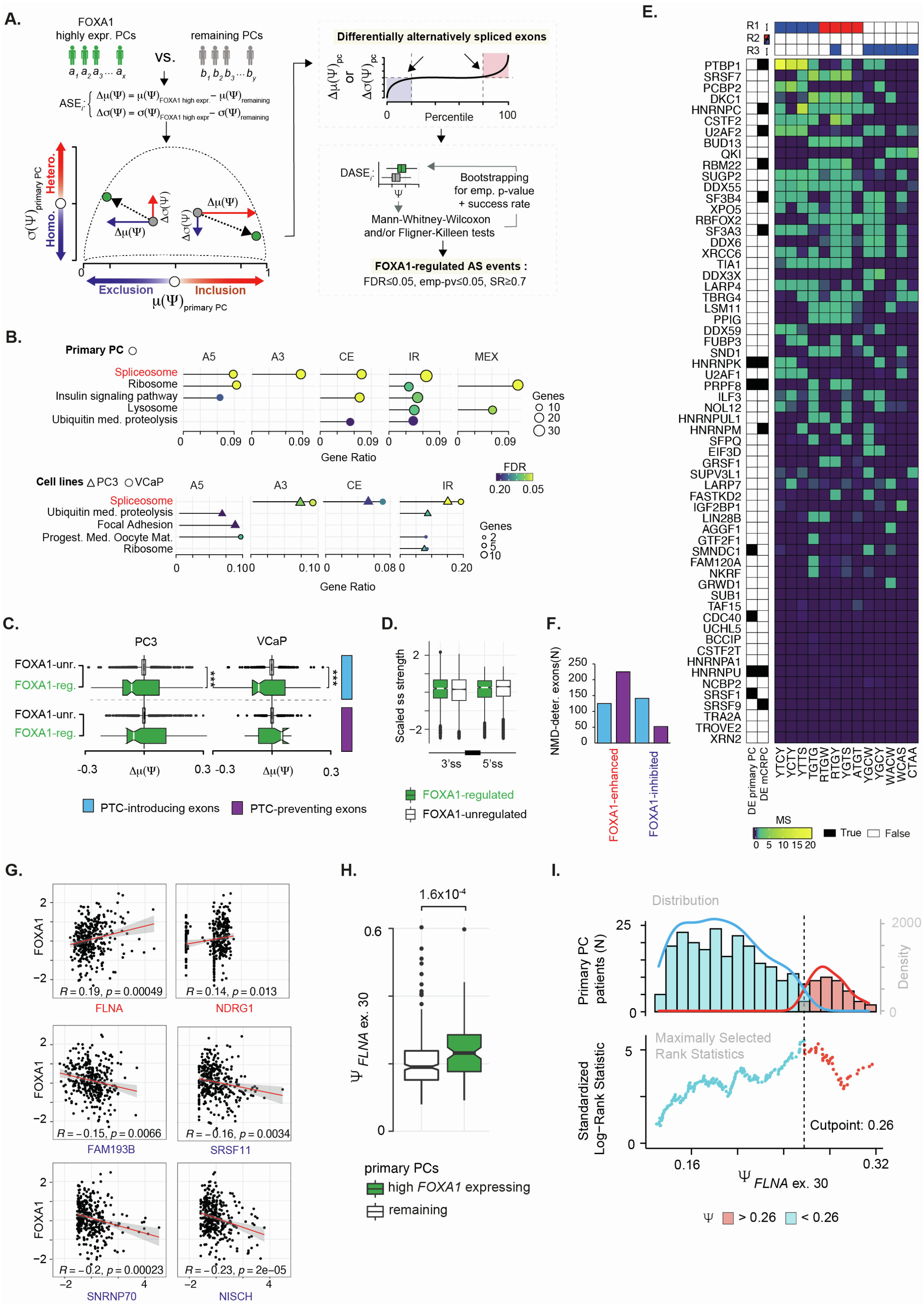

Figure S5. (A) Schematic representation of the pipeline used to identify FOXA1-regulated events in primary PC. Alternatively spliced exons (ASEs) are defined by mean (μ) and standard deviation (σ) inclusion (Ψ) changes (Δ) between

high FOXA1 expression tumors and remaining ones. Trajectory (represented as the arrow) of each ASEs is defined by $\Delta\mu(\Psi)$ and $\Delta\sigma(\Psi)$ changes (Δ) toward high FOXA1 expression. (B) Over representation analysis performed on genes harbouring FOXA1-regulated AS events, stratified by event type, in primary PCs and PC cell lines. Shape size and gene ratio indicate the number and the fraction of selected genes in each gene set, respectively. Color key represents the significance of the enrichment. The top five gene sets with FDR<0.25 are shown. CE, Cassette Exon; A3, Alternative 3' splice site; A5, Alternative 5' splice site; IR, Intron Retention; MEX, Mutually Exclusive exons. (C) Distribution of mean inclusion changes of NMD-determinant FOXA1-regulated and -unregulated exons in VCaP (upper panel) and PC3 cells (lower panel). Stars indicate statistical significance of a two-tailed Wilcoxon Rank Sum test (***, p-value$<10^{-3}$). (D) Box plots showing the scaled MaxEntScan strength of the 3' and 5' splice sites in FOXA1-regulated (green) and -unregulated (white) CEs. (E) Heatmap showing the association between enriched multivalent RNA motifs and cognate SRGs in terms of maximum Matching Score (MS). Top annotation heatmap shows indicates the regions at exon/intron junctions where motifs were enriched at inhibited (blue) or enhanced (red) exons. Left annotation indicates whether SRGs are DE in primary PCs or mCRPCs. (F) Number of NMD-determinant exons that are inhibited (blue) or enhanced (red) by FOXA1. (G) Scatter plots between scaled inclusion level of each of the six harmful NMD-determinant exons and scaled expression of *FOXA1* in primary PC. Pearson's correlation and corresponding p-value are shown for each plot. (H) Distributions of *FLNA* exon 30 inclusion levels (Ψ) across primary PC samples stratified on high *FOXA1* expression. The significance of a two-tailed Wilcoxon Rank Sum test comparing the two groups is shown. (I) Distribution of the standardized Log-Rank statistic as a function of the cutpoint on *FLNA* exon 30 Ψ (bottom panel). Top panel shows the distribution of the number of primary PC patients as a function of *FLNA* exon 30 Ψ. Patients are stratified according to the cutpoint. Density distributions of the two groups are superimposed.

Figure S6. FOXA1 regulates AS regardless of tumor purity constraints, related to figures 1 and 2.

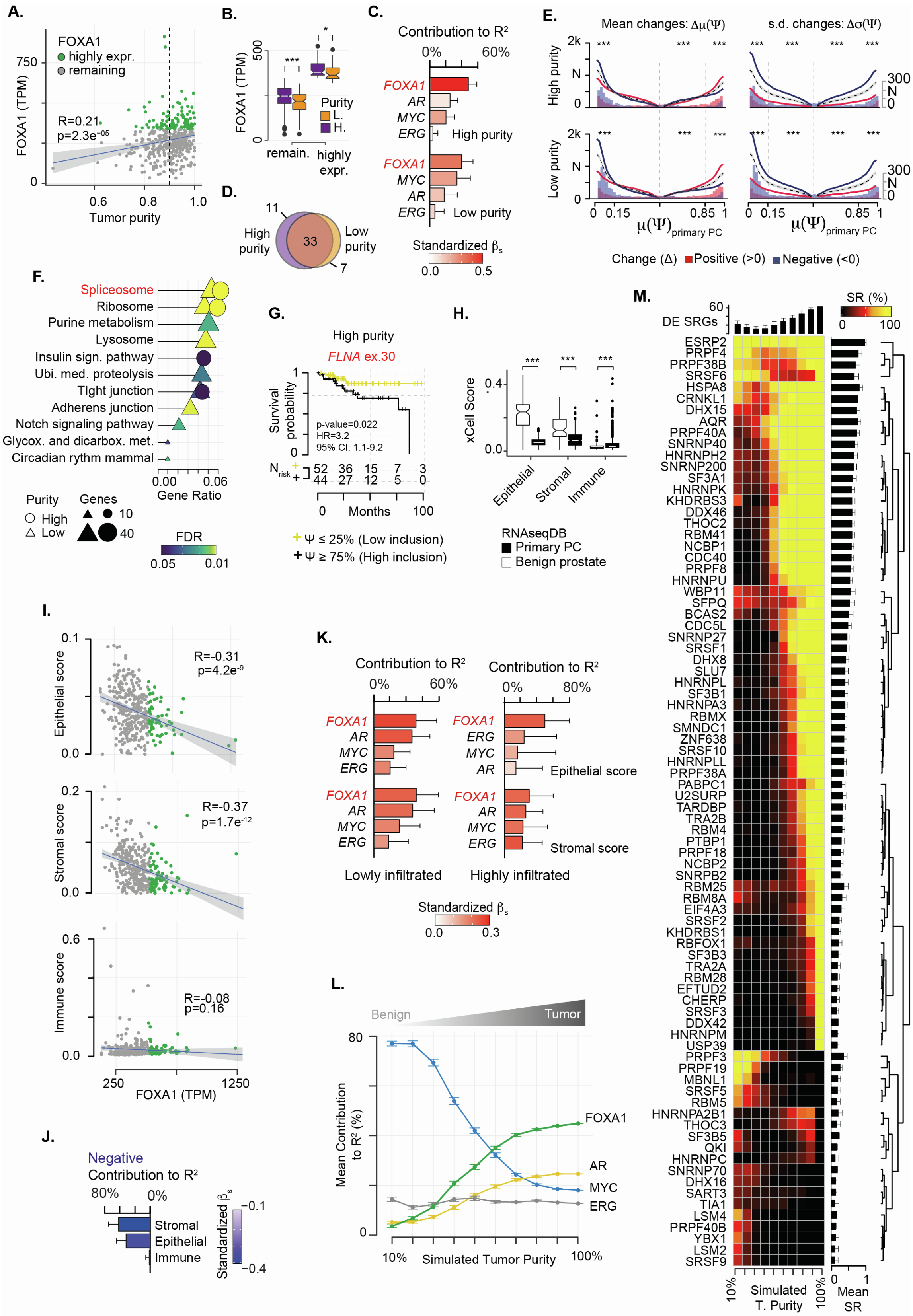

Figure S6. (A) Scatter plot of *FOXA1* expression (TPM) and tumor purity for primary PCs. Dot color indicates sample stratification according to high *FOXA1* expression (i.e. ≥75th percentile of expression distribution). Pearson's correlation coefficient and p-value are shown. 90% of samples show less than 20% of contamination (median purity = 91%) (B) Boxplots represent *FOXA1* expression levels in primary PCs with high FOXA1 expression and remaining ones stratified into "high purity" (i.e. purity ≥ 90%) and "low purity" (i.e. purity < 90%) samples. High purity groups exhibit a greater overall expression of FOXA1 than the low purity groups. This is consistent with a greater infiltration of low purity samples with normal non-cancerous cells, which express FOXA1 at lower levels than cancer cells (Parolia et al. 2019). (C) Results of multivariable covariance analysis between the cumulative expression of SRGs and the expression of TFs in primary PCs for the high purity and low purity samples. Color key indicates the standardized β coefficients of the model. FOXA1 is the strongest contributor to SRG expression regardless of purity constraints. In the high purity tumors, FOXA1 contributes to SRG expression significantly more than the second-ranked TF (two-sided Z-test p-value=0.01). (D) Venn diagram depicts 40 and 44 differentially expressed (DE) SRGs by FOXA1 in the "high" and "low purity" samples, respectively. 73% of DE SRGs were still differentially expressed by FOXA1 in both "high" and "low purity" samples (E) The cumulative distribution of the number (N) of exons with either positive (red) or negative (blue) changes are reported ranging from $\mu(\Psi)_{\text{primary PC}}$ of 0.5 (*i.e.* mixed isoforms) to the boundaries of 0 and 1 (*i.e.* dominant isoforms) model for the high purity and low purity samples. Dashed lines represent the expected mean cumulative distribution of events with inclusion changes generated by 1,000 Monte Carlo simulations. Grey area represents confidence intervals (5%-95%). Histograms of the number (N) of exons with positive (red) and negative (blue) changes are superimposed on the x-axis, respectively. Stars indicate the significance of two tailed Exact Binomial test comparing the abundances of exons with positive and negative changes against a null hypothesis of equal probability (*i.e.* 0.5) in four groups of inclusion levels (***, p-value<10^{-3}). FOXA1-mediated AS calibration towards dominant isoforms is confirmed in both cohorts. (F) Enrichment of genes affected by FOXA1-regulated AS events in the high purity (circles) and low purity (triangles) group of samples. Shape size and gene ratio indicate the number and the fraction of selected genes in each pathway, respectively. Shape size ranges from 10 to 40. Color key represents the statistical significance of the enrichment. Only significantly enriched pathways (FDR<0.1) are shown. The enrichment of AS events in spliceosomal genes is confirmed in both cohorts. (G) Kaplan-Meier plot of disease-free survival for primary PC patients in the "high purity" group with low and high inclusion of *FLNA* exon 30. The number of patients at risk (N_{risk}) is tabulated at each time point on the x-axis. Univariate hazard ratio with 95% confidence intervals (CI) and two-tailed log-rank test p-value are shown. (H) Benign epithelial, stromal and immune xCell score distribution in primary PC and benign prostate tissue samples (***, Wilcoxon Rank Sum test p-value<10^{-3}). As expected, stromal and epithelial scores are enriched in benign tissues compared to tumors, whereas immune score is greater in primary PCs relative to benign tissues (I) Scatter plots between *FOXA1* expression and epithelial (top panel), stromal (middle panel) and immune (bottom panel) xCell scores in primary PC. Pearson's correlation coefficients and p-values are shown. Stromal and epithelial scores negatively correlate with *FOXA1* expression, indicating a lower infiltration in samples with high *FOXA1* expression. FOXA1 expression is not affected by the infiltration rate of immune cells. These infiltrate-specific scores are low in tumors (*i.e.* median score = 0.044 (stromal), 0.036 (epithelial), and 0.014 (immune)), in line with the average high purity of these primary PCs. (J) Results of multivariable covariance analysis between *FOXA1* expression and the epithelial, stromal and immune xCell score. Color key indicates the standardized β coefficients of the model. (K) Results of multivariable covariance analysis between the cumulative expression of SRGs and the expression of TFs in primary PCs stratified into "highly" and "lowly infiltrated" according to high values of epithelial and stromal xCell score (*i.e.* ≥75th percentile of each distribution). Color key indicates the standardized β coefficients of the model. FOXA1 is the strongest contributor to SRG expression regardless of the level of stromal and benign epithelial cell infiltration. (L) Mean relative contribution of each TF the cumulative expression of SRGs measured by multivariable covariance analysis in primary PCs at different levels of simulated tumor purity. Error bars indicate the standard error of the mean contribution across 100 Monte Carlo simulations. Increasing tumor purity (*i.e.* reducing normal cell infiltrates) potentiates the contribution of FOXA1 to SRG expression. (M) Heatmap showing, for each SRG, the success rate (SR, *i.e.* percentage of success across 100 Monte Carlo simulations) of being differentially expressed upon high *FOXA1* expression in the different simulated purity levels. Bar plots at the top indicate the average number (N) of differentially expressed SRGs for each purity level across simulations. Bar plots on the right indicate the average SR for each SRG across simulations. For both bar plots, error bars indicate the standard deviation. The number of SRGs that were DE by FOXA1 increases with tumor purity.
